# The effectiveness of adapted psychological interventions for people from ethnic minority groups: A systematic review and conceptual typology

**DOI:** 10.1016/j.cpr.2021.102063

**Published:** 2021-08

**Authors:** Laura-Louise Arundell, Phoebe Barnett, Joshua E.J. Buckman, Rob Saunders, Stephen Pilling

**Affiliations:** a Centre for Outcomes Research and Effectiveness, Research Department of Clinical, Educational and Health Psychology, University College London, London, UK; b National Collaborating Centre for Mental Health, Royal College of Psychiatrists, London, UK; c iCope, Camden and Islington Psychological Therapies Services, Camden & Islington NHS Foundation Trust, St Pancras Hospital, London, UK

**Keywords:** Systematic review, Ethnic minorities, Racial minorities, Refugee, Mental health, Psychological interventions

## Abstract

This review assessed the efficacy of adapted psychological interventions for Black and minority ethnic (BME) groups. A conceptual typology was developed based on adaptations reported in the literature, drawing on the common factors model, competence frameworks and distinctions between types of cultural adaptations. These distinctions were used to explore the efficacy of different adaptations in improving symptoms of a range of mental health problems for minority groups. Bibliographic searches of MEDLINE, Embase, PsycINFO, HMIC, ASSIA, CENTRAL, CDSR and CINAHL spanned the period from 1965 to December 2020. Adaptations to interventions were categorised: i) treatment specific: therapist-related, ii) treatment-specific: content-related and iii) organisation-specific. Meta-analyses of RCTs found a significant effect on symptom reduction when adapted interventions were compared to non-adapted active treatments (*K* = 30, Hedge's *g* = -0.43 [95% CI: -0.61, -0.25], *p* < .001). Studies often incorporated multiple adaptations, limiting the exploration of the comparative effectiveness of different adaptation types, although inclusion of organisation-specific adaptations may be associated with greater benefits. Future research, practitioner training and treatment and service development pertaining to adapted care for minority groups may benefit from adopting the conceptual typology described.

## Highlights

• Ethnic minority groups benefit from adapted psychological interventions
• Identifying the most efficacious adaptation types is challenging
• Services might consider organisation-specific adaptations
• Consistency is needed in the definitions of cultural adaptations

## 1 Introduction

Substantial inequalities have been identified in mental health care, with people who belong to Black and other minority ethnic (BME) groups experiencing sub-optimal treatment for their mental health problems (Halvorsrud, Nazroo, Otis, Brown Hajdukova, & Bhui, 2018; Lawlor, Johnson, Cole, & Howard, 2012; Singh, Islam, & Brown, 2013). BME people may experience challenges in both access to and experience of mental health treatment (Barnett et al., 2019; Bhui et al., 2015; Pilkington, Msetfi, & Watson, 2012). Problems include: a lack of information for service users about the availability of, and routes into treatment (Bogenschutz, 2014; Dowrick, Chew-Graham, & Lovell, 2013; Memon et al., 2015); a lack of involvement in treatment decision-making with clinicians, and problems with the appropriateness of treatment offered (Benish & Wampold, 2011; Sekhon, Cartwright, & Francis, 2017). Other problems such as stigma within BME communities (Franks, Gawn, & Bowden, 2007; Knifton, 2012) and financial barriers to care are also reported (Kim, Vonneilich, Lüdecke, & von dem Knesebeck, 2017; Mojtabai, 2005). These issues are often magnified for people who are forcibly displaced such as refugees and asylum seekers, as they may have additional difficulties accessing appropriate care due to language barriers, a lack of medical insurance or problems in meeting requirements for registration with healthcare services; all of which are frequently associated with complex needs (Byrow, Pajak, Specker, & Nickerson, 2020; Franks et al., 2007).

It has been suggested that some of the poorer outcomes and engagement observed for ethnic minority groups are a consequence of the interventions typically provided, which have been based on Western concepts of mental disorder (Fernando, 2010), and do not necessarily reflect appropriate cultural perceptions of mental health (Codjoe, Byrne, Lister, McGuire, & Valmaggia, 2013) or broader cultural and social diversity (Gopalkrishnan, 2018). This is problematic given the role that culture plays in shaping beliefs, including the ways in which mental health is understood (Fernando, 2010).

There are an increasing number of studies which seek to either test culture-based adaptations to existing interventions or develop novel interventions for specific minority, ‘under-served’, or ‘heard-to-reach’ communities (Arundell et al., 2020; Clarke et al., 2013). A number of systematic reviews have explored the effectiveness of culturally adapted interventions, suggesting some benefits of culturally adapted care (Bhui et al., 2015; Chowdhary et al., 2014; Escobar & Gorey, 2018; Huey & Tilley, 2018). However, there is a lack of clarity about the definition or the efficacy of specific adaptations (Healey et al., 2017). Existing reviews have rarely distinguished between the types of adaptations made. The lack of agreed definitions of what constitutes a cultural adaptation (Barrera, Castro, Strycker, & Toobert, 2013; Bernal, Jiménez-Chafey, & Domenech Rodríguez, 2009; Tseng, 1999) has also made it difficult to assess the degree to which an intervention has been culturally adapted. This has limited the identification of the most efficacious interventions and limited services’ capacity to improve the outcomes of culturally adapted interventions for BME populations. The lack of a consensus for describing the best methods for adapting interventions, as well as the gaps in knowledge as to the degree to which adaptation types might influence outcomes, suggests a new approach to assessing the effectiveness of cultural adaptions may be of benefit.

This paper seeks to address the lack of clarity by developing a typology drawing on a broad range of studies of adaptations to psychological interventions for BME groups. The typology is intended to be broad in scope taking into account the common and specific factors associated with the effectiveness of interventions (Frank, 1971; Grencavage & Norcross, 1990; Wampold, 2015); personnel, cultural and linguistic factors in the delivery of the interventions (Barrera et al., 2013; Bernal et al., 2009; Healey et al., 2017) and wider organisational factors such the location of services or pathways into care (Moffat, Sass, Mckenzie, & Bhui, 2009; Thornicroft, Deb, & Henderson, 2016; Volpe, Mihai, Jordanova, & Sartorius, 2015). Construction of the typology drew upon the work on common factors and that of Roth and Pilling (2018) on the development of competence frameworks for psychological interventions. Roth and Pilling used an ‘architecture’ of common and specific therapeutic competences, and higher order organisational competences to develop a framework for the development and implementation of a national training program in psychological therapies in the UK (Clark, 2011). The typology aims to support a fuller understanding of the use and effectiveness of the various approaches to cultural adaptation, and thereby improve the delivery and outcomes of psychological interventions for people from ethnic minority groups.

Further, the typology aims to provide a clearer conceptualisation of which adaptations are effective. A better categorisation of adaptation types which takes into account the multiple components of treatment could support the improved provision of adapted care. In order to do this, there is a requirement to consider not only the content of psychological interventions (including those factors common across treatments) but also other factors impacting the provision of mental health interventions, such as service design and delivery considerations. Adaptations in line with these considerations may be informed by cultural knowledge and its interface with existing service structures. Understanding the particular impact and importance of different adaptation types could inform treatment developers and providers about the optimal approaches to adapting interventions to better support people from minority and marginalised groups.

This review also explores the effectiveness of different psychological adaptations, using the typology to structure a meta-analytic exploration of randomized controlled trials (RCTs) which have assessed the efficacy of the adapted psychological interventions. The typology is used to explore and understand adaptations to the aspects of treatment that are: common across interventions, such as establishing a good therapeutic relationship or ensuring intervention content is suited to the person; specific aspects of the intervention, such as the person providing it, the insertion of culturally-congruent terms, the language in which the intervention is provided or specific adaptations of therapeutic technique; and organisational or service level factors, such as the treatment location or the route through which treatment is accessed.

Given that poorer outcomes for ethnic minority groups are thought to in-part be a consequence of the lack of cultural suitability of interventions typically provided, the exploration of the impact of adapted interventions that are aimed at certain groups was also considered important to explore in this paper; to see what might be beneficial for whom. Whilst grouping people in terms of ethnicity has significant limitations, not least due to the complexity of what constitutes both individual and group identity (Fernando, 2010), the ways in which cultural and ethnic identity intersect (including in the establishment of one's values, perceptions, behaviours and beliefs) (Dogra, 2010) supports the exploration of the impact of adaptations on people of shared ethnicity. A better understanding of the effectiveness of adapted interventions for different groups of people who may be more likely to have shared cultural values, could help with identifying and implementing suitably adapted interventions or pathways into care for people who identify as belonging to these groups.

In summary the review sought to address the following questions:

1) What types of adaptations have been implemented?

2) Are the types of culturally adapted interventions differentially effective?

3) What effects of culturally adapted interventions are found across different ethnic groups?

## 2 Methods

This systematic review and meta-analysis was registered on PROSPERO (ID: CRD42019127610) and reported in accordance with PRISMA guidelines (Moher, Liberati, Tetzlaff, Altman, & Group, 2009). The review adhered to the registered protocol with the exception of the following deviation: a decision was made to also include adapted interventions that were self-administered or self-help interventions (Note, there is good evidence for the effectiveness of self-help interventions (Garrido et al., 2019; Matcham et al., 2014) which have been used to try and improve access to psychological therapies for ‘hard to reach’ groups).

In developing the typology and to address Research Question 1: ‘What types of adaptations have been implemented?’, studies with a range of designs were used (including non-controlled pre-post studies) to gain a comprehensive understanding of reported adaptations across the literature. Research Question 2: ‘Are the types of culturally adapted interventions differentially effective?’, and Research Question 3: ‘What effects of culturally adapted interventions are found across different ethnic groups?’, were addressed only using evidence from randomized controlled trials (RCTs) which assessed the efficacy of the adapted psychological interventions at the end of treatment.

### 2.1 Search strategy

The following bibliographic databases were systematically searched: MEDLINE, Embase, PsycINFO, HMIC (via Ovid), ASSIA (via Pro Quest), Cochrane Central Register of Controlled Trials (CENTRAL), CDSR (via Wiley) and CINAHL. Search dates were 1965-11th December 2020. A search of the reference lists of identified systematic reviews was included to identify other studies with potential for inclusion. The full search strategy is available in **Appendix A**.

### 2.2 Inclusion criteria

#### 2.2.1 Participants

• Adults, 18+ years old
- Studies inclusive of participants under 18 years old were included only if more than 50% of participants were 18 or above or the focus of the study was on adult mental health.
• Black, ethnic minority, migrant, refugee or asylum seeker communities, and people referred to as ‘minorities’ or defined as belonging to an identified racial or ethnic ‘minority group’
- Terms were informed by the race and ethnicity descriptors used by the UK 2011 Census from the Office of National Statistics (Office for National Statistics, 2011) and the United States Census Bureau (United States Census Bureau, 2020). Both were used to inform terms due to the variation in how these labels are understood and applied to refer to different ethnic groups between continents (for example, the term ‘Asian’ can have different connotations and uses in the USA compared to the UK).
• People experiencing symptoms of, or diagnosed with mental health conditions or problems, excluding:
- people with autism spectrum disorders, attention deficit hyperactivity disorders and people with (and interventions aimed to treat) organic disorders, dementia or cognitive decline as a result of acquired cognitive or neurological impairment
- people receiving psychological treatment primarily for non-mental health disorders, including those described by the authors as ‘stress’ (studies where stress was measured were included if the primary target condition was a diagnosable mental health disorder).

#### 2.2.2 Interventions

• Any psychological intervention delivered as treatment for a mental health problem that was intentionally adapted, changed or modified to better support people from BME communities, excluding:
- non-evidence based or alternative therapies (evidence-based interventions were taken to include those supported by one of the recognised registries of interventions, including the American Psychological Association catalogue of interventions; National Registry of Evidence Based Practice or the National Institute for Health and Care Excellence); novel interventions (i.e., no clear adaptation of an evidence-based treatment);
- adapted interventions which combined psychological and pharmacological treatments (studies were not excluded if pharmacological treatment was already underway or if it constituted ‘treatment as usual’);
- interventions which aimed to treat organic disorders, dementia or cognitive decline as a result of a neurological condition.

Details on the types of adaptations including how these were classified these, are provided in the ‘Development of the typology’ section of this review.

#### 2.2.3 Comparator

• Active controls, including non-adapted or standard treatment, or waitlist/no intervention controls. Due to discrepancies in reporting between studies, those which defined the control group as receiving ‘treatment as usual’ were checked and categorised as ‘active’ or ‘waitlist/no intervention’ on the basis of information provided in the study about whether the control group received any intervention.

#### 2.2.4 Outcomes

• Treatment effectiveness; the primary outcome is the effect of the intervention on symptom severity as measured using appropriate clinical outcome measures post-treatment.

#### 2.2.5 Study designs

• RCTs (including pilot studies), quasi-experimental and observational studies reporting post treatment outcomes.
- As outlined above, a range of study designs were used to develop the typology and address Research Question 1; only the RCTs were used to address Research Questions 2 and 3.

### 2.3 Screening

All studies were screened at title/abstract-level by L-LA according to the pre-determined inclusion criteria. Remaining studies were filtered at full-text. PB reviewed 10% of references at each stage resulting in 95% agreement. Conflicts were resolved by consensus in meetings with SP. Screening was undertaken using the Rayyan application (Ouzzani, Hammady, Fedorowicz, & Elmagarmid, 2016).

### 2.4 Data extraction

Data extraction tables were prepared using Microsoft Excel to extract the following data: study design; ethnic group descriptor; name of intervention; information about the adaptation(s) as reported and methods used; information about the original intervention to which adaptations were made; type of control (active [non-adapted or other treatment] or waitlist/inactive [no treatment or delayed treatment]); primary mental health condition targeted (target condition for each study represents the condition, or symptoms of the condition which the study authors reported as the primary mental health outcome measure); primary outcome measure used; primary outcome measure scores (at end of treatment); duration of treatment; exact length of follow-up (as applicable); studies’ methodological characteristics to inform the assessment of quality, including risk of bias. Where sufficient data was not provided in published articles or supplementary material, study authors were contacted. Data was extracted by L-LA and 10% of extractions were validated by PB for accuracy, with consistent results.

### 2.5 Development of the typology

#### 2.5.1 Types of adaptations

Data extracted included information on adaptations as they were reported by study authors (as detailed above). This was regardless of whether adaptations were reported explicitly (e.g., the intervention was referred to as a ‘culturally adapted treatment’ by the authors), including where authors used an existing adaptations framework to guide them, or if modifications and adaptations were apparent but were described or presented with little detail or supporting information. A distinction was drawn between adaptations focused on changes to the way in which interventions are provided, which are referred to here as ‘treatment-specific’ cultural adaptations, and the systems that support the service-level provision and delivery of the interventions*,* referred to here as ‘organisation-specific’ cultural adaptations. Supplementary detail of the development of the typology and its evolution, including previous definitions and iterations is given in **Appendix B**.

##### 2.5.1.1 Treatment-specific cultural adaptations

To further characterise treatment-specific cultural adaptations, approaches which have attempted to identify factors thought to underpin the efficacy of psychological interventions were drawn upon. In this area, there is a strong emphasis placed on common factors which are considered to be present across all types of psychological interventions (Drisko, 2004; Frank, 1971; Grencavage & Norcross, 1990; Luborsky, Singer, & Luborsky, 1975; Wampold, 2015). The key common factors are:

1) Establishing a good therapeutic relationship, the quality of which is regarded as being crucial to the success of psychotherapy, (Benish and Wampold, 2011). The therapeutic alliance is central to this relationship and is underpinned by trust and respect (Wampold, 2015; Wampold & Budge, 2012), conveys empathy, provides a supportive environment (Amole et al., 2017; DeRubeis, Brotman, & Gibbons, 2005) and establishes reasonable expectations and collaborative agreement of goals (Drisko, 2004; Lambert, 1992). Adaptations in this area focus on changes to support the development of the therapeutic relationship, for example using a pre-intervention discussion to establish rapport with a patient or taking an approach to communication that is based on cultural values held to be important for a particular community to which the patient identifies as belonging.
2) Ensuring acceptability and suitability of treatment content, independent of the treatment being delivered. This is integral to treatment efficacy in the common factors model, where cultural adaptation is emphasised (Benish et al., 2011; Lambert, 1992). This is the common factor most frequently focused on in research on improving care for minority groups (Antoniades, Mazza, & Brijnath, 2014; Degnan et al., 2018; Pineros-Leano, Liechty, & Piedra, 2017; Van Loon, Van Schaik, Dekker, & Beekman, 2013). Other components to achieve acceptability include psychoeducation and preparing the patient for psychological therapy by socialising them to the process of treatment and to the treatment model (DeRubeis et al., 2005; Drisko, 2004; Lambert, 1992).

While both areas (1) and (2) involve making adaptations specific to treatment, (1) is focussed on the therapist and therapist skills, whilst (2) focuses on the content of the intervention(s). In addition, other specific adaptation types were identified that could be classified as being therapist-related (such as use of a bilingual provider or ethnic matching of therapist to patient), and others which could be classified as content-related (including cultural modifications to materials, resources use of terms) as well as using translated materials or incorporating faith/religious beliefs into treatment).

##### 2.5.1.2 Organisation-specific cultural adaptations

Organisation-specific cultural adaptations encompass service design and delivery, which are informed by cultural knowledge and its interface with existing service structures. Such adaptations can include changes to the time or length of the intervention, to the place it is provided, putting measures in place so that treatment can be accessed more easily (for example by out-reach work with BME communities to develop more effective pathways into care), or changing the form used to provide treatment (for example, providing interventions remotely or in a group setting).

This conceptual typology of treatment-specific cultural adaptations and organisation-specific cultural adaptations was used as the basis for the quantitative analyses which follow.

### 2.6 Quality assessment

RCTs were included in the meta-analyses and methodological quality was assessed using the Cochrane Risk of Bias tool (Higgins et al., 2011), which is specific to RCTs. Studies were considered to be of low, unclear, or high risk of bias depending on judgements of selection, performance, detection, attrition, and reporting bias (**Appendix C**).

### 2.7 Data analysis

Data from all included studies were extracted and used to develop the typology, to examine the different adaptation types identified and answer Research Question 1. Study characteristics and data extracted about adaptations reported in each study are included in **Appendices C-E**. Analyses were conducted from eligible RCTs to answer Research Questions 2 and 3 on the effectiveness of adapted interventions. Meta-analyses were performed in R using the ‘metafor’ package (Viechtbauer, 2010). Standardised mean difference (SMD) effect size estimations were calculated using raw mean scores and standard deviations. The metafor package automatically corrects for the positive bias of SMD, producing Hedge's *g* (Hedges, 1981). Hedges *g* allows for comparison of outcomes across studies which have used different outcome measures by pooling variances and standardising outcomes (Lipsey & Wilson, 2001). Random-effects models were used. Heterogeneity was calculated using the *I*^2^ statistic, and interpreted using the following tentative classifications: 0% - 40%: unimportant, 30%-60%: moderate, 50% -90%: substantial and 75% - 100%: considerable (Higgins & Green, 2011). Sub-group analyses assessed the effectiveness of interventions grouped on the basis of different adaptations, target population and control condition type (active, or waitlist/no intervention). Heterogeneity between sub-groups was tested using Cochran's Q. Meta-regressions were used to explore the degree to which different factors (including adaptation type and mental health problem) influenced effects. Analyses of the following extracted outcomes was not included due to limits of available data: relapse rates; treatment attrition; hospital/treatment admissions; wellbeing/quality of life measures.

## 3 Results

**Fig. 1:**
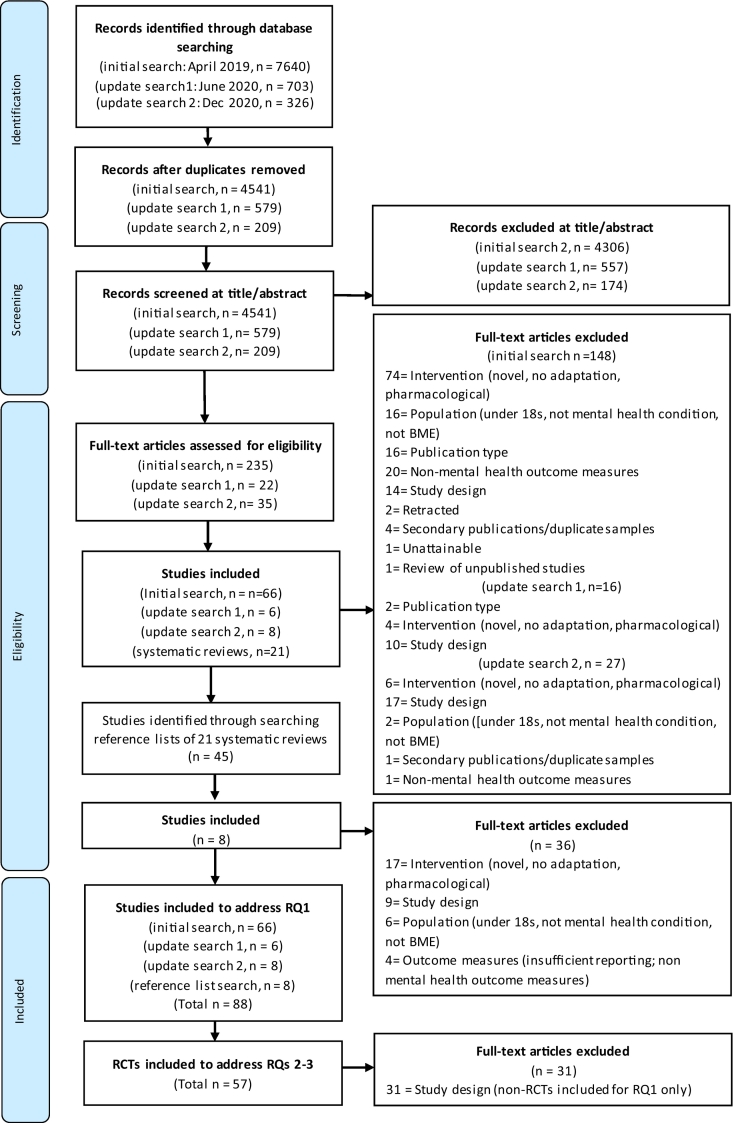
PRISMA flow diagram of study selection.

Eighty-eight studies met inclusion criteria and were used in the development of the typology and to address Research Question 1. Of these studies, 67 were RCTs (including pilots), 15 were pre-post designs and 2 were cohort studies. The remaining 3 studies included 2 non-randomized quasi-experimental studies and a randomized trial using a convenience sample. Of the RCTs, 57 provided post-treatment outcome measures with complete and relevant data, making them eligible to be included in meta-analyses to address Research Questions 2 and 3. A PRISMA flow diagram of study selection is presented in Fig. 1, while further details on search results and study flow are provided in **Appendix F.** For study characteristics of all 88 studies see **Appendix, Table C.1.**

### 3.1 Research question 1: types of adaptations implemented

#### 3.1.1 Adaptation types

**Fig. 2:**
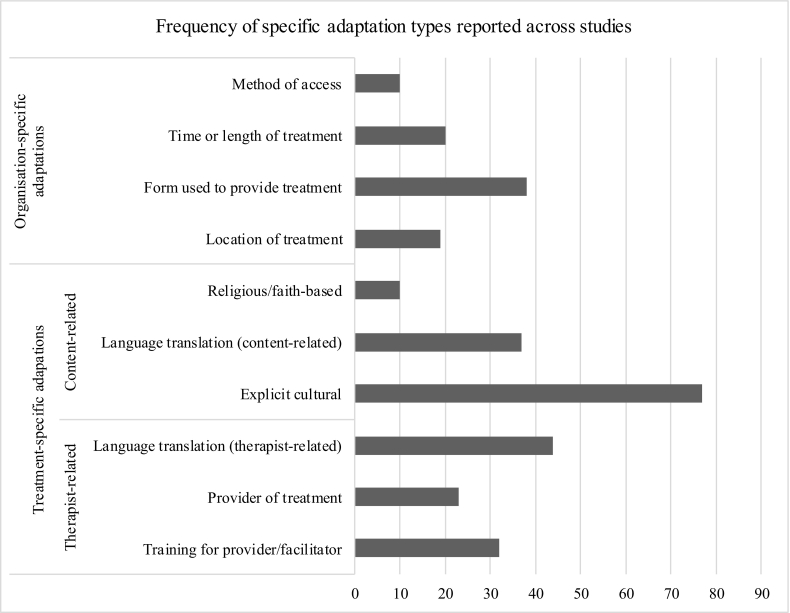
Frequency of specific adaptations made across all studies (total studies: K = 88).

The conceptual typology developed (presented in Table 1) was used to describe the types and frequencies of different adaptations to interventions for BME groups using all 88 included studies. Each study was categorised according to the adaptations it reported and the overarching adaptation area was recorded for each study. These are labelled as ‘treatment-specific’ (including therapist-related and content-related adaptations) and ‘organisation-specific’ (further details of the evolution of these terms is provided in **Appendix B)**. Also recorded were further differentiations of adaptions, including those involving common factors (pertaining to the therapeutic relationship and acceptability and suitability) and where studies reported specific adaptations at the treatment or organisational level. Most studies (80%) included more than one adaptation across multiple areas. Fig. 2 shows the frequency of specific adaptations reported.

**Table 1:** Conceptual typology of adaptations.

**Treatment-specific adaptations -***Adaptations to intervention provided*					
	**Common Factors**	**Specific adaptations**			
**Therapist-related adaptations** Focus on person(s) delivering intervention	**Therapeutic relationship** Alliance and Empathy; Agreement of treatment goals; Expectations of treatment; Patient feedback	**Training for therapist/provider/facilitator** Training for professional; Training for layperson	**Language translation** Use of interpreter or same language/bilingual provider	**Provider of treatment** Ethnic matching (between patient and provider); Lay person or paraprofessional; Community or religious leader	
**Content-related adaptations** Focus on content of the intervention	**Acceptability and suitability** Treatment structure; Provision of education components; Provision of components to support preparation of the patient	**Language translation** Translated materials/resources	**Religious/faith-based adaptations** Modified materials or resources; Use of religious texts, doctrine or guidance; Involvement of religious figure		
	**Explicit ‘cultural’ adaptation of intervention content**⁎ Culturally modified materials/resources; Culturally-sensitive or congruent terms; Emphasis on cultural norms/expectations; Theoretical stance culturally informed				
**Organisation-specific interventions -***Adaptations at organisation/service level to provide intervention*					
		**Specific adaptations**			
		**Location of treatment** Care at home; Care in the community; Care in non-healthcare setting	**Form used to provide treatment** Face to face; Telephone; Digital; Group treatment	**Time or length of intervention** Variation in intervention or session length; Time of day	**Method of access** Rapid or accelerated access; Access route (e.g., via alternative to standard route)

⁎ Explicitly reported cultural adaptations were considered in the typology with regard to both their inclusion in the common factors model and as a specific type of adaptation.

More detail on the typology of adaptations, including examples of adaptations from select studies is provided below. Further details on the adaptations reported for all studies are provided in **Appendix E**.

##### 3.1.1.1 Treatment-specific adaptations

Regarding treatment-specific adaptations, 53 (60%) of the 88 studies made therapist-related adaptations and 82 (93%) made content-related adaptations.

###### 3.1.1.1.1 Therapist-related adaptations

**Training for therapist/provider/facilitator:** Thirty-two studies (36%) reported adaptations that involved the specific provision of training for whomever was providing the intervention. Examples include where training was provided to professionals (e.g., Kananian, Soltani, Hinton, & Stangier, 2020; Miranda, Azocar, Organista, Dwyer, & Areane, 2003) or to lay/community members (e.g., Bonilla-Escobar et al., 2018; Leiler, Wasteson, Holmberg, & Bjärtå, 2020) for the purpose of providing better care for the target population group.

**Language translation-therapist:** Forty-four studies (50%) reported intentional provision of a same-language or bilingual provider, or use of an interpreter in the intervention delivery as an adaptation to the treatment (e.g., Kanter et al., 2015; Miranda et al., 2003; So et al., 2015).

**Provider of treatment:** Twenty-two studies (25%) reported changes made to the provider or facilitator, including where ethnic matching was applied (i.e., the person providing the treatment identified as the same or similar ethnicity to the person receiving the treatment (e.g., Alegria et al., 2014; Comas-Díaz, 1981; Cooper et al., 2013). Provider adaptations also involved having a para-professional, lay person or community member (i.e., a non-professional) leading or taking a key role in the delivery of the intervention (e.g., de Graaff et al., 2020; Hovey, Hurtado, & Seligman, 2014; Leiler et al., 2020).

**Therapeutic relationship (common factors element):** Forty-two studies (48%) reported adaptations made to impact the therapeutic relationship, including those intended influence empathy or improve therapeutic alliance between the patient and person providing the intervention, (e.g., Bedoya et al., 2014), making specific considerations to the development of the therapeutic relationship based on previous work with demographically similar groups of patients (e.g., Beeber et al., 2010) or using a treatment provider who shared a similar ex*peri*ence to the person(s) receiving treatment, e.g. experience of displacement, (Bonilla-Escobar et al., 2018). Studies that reported adaptations made to support agreement of treatment goals between both parties and efforts made to set expectations for the intervention for the group receiving treatment were also included in this category (e.g., Bolton et al., 2003) as were occasions where structured patient feedback on the intervention was collected (e.g., Gallagher-Thompson et al., 2007; Hwang et al., 2015).

###### 3.1.1.1.2 Content-related adaptations

**Explicit cultural adaptations (both a common factors element and specific adaptation type):** Seventy-seven studies (88%) referred to cultural adaptations explicitly, including where they reported use of culturally-modified materials or resources, for example adapting vignettes with scenarios reflecting local life of the target group to improve relatability (e.g., Ryan, Maurer, Lengua, Duran, & Ornelas, 2018) incorporation of culturally congruent terms/language (e.g. Grote et al., 2009; Lovell et al., 2014) including use of metaphors, or where intentional emphasis was placed on the cultural norms, practices or expectations of the target population (e.g., Gonyea, Lopez, & Velasquez, 2016; Knaevelsrud, Brand, Lange, Ruwaard, & Wagner, 2015). Explicit cultural adaptations also involved those where the authors reported that the theoretical stance of the intervention was culturally informed including use of existing theoretical models or adaptations frameworks (e.g., Bedoya et al., 2014; de Graaff et al., 2020; Hinton et al., 2005; Kananian et al., 2020; Koch, Ehring, & Liedl, 2020; Pan, Huey, & Hernandez, 2011). Explicitly reported cultural adaptations were considered in the typology with regard to both their inclusion in the common factors model and as a specific type of adaptation (explored in the analyses later).

**Language translation – content-related:** The provision of treatment materials and resources translated into another language (e.g., Collado, Calderon, MacPherson, & Lejuez, 2016; Dahne et al., 2019; Lindegaard et al., 2020) was reported for 37 studies (43%).

**Religious/faith-based adaptations:** There were 10 studies (11%) where religious, faith-based or spiritual beliefs were incorporated into the intervention including where faith-based modifications were made to treatment materials or resources, where a religious person (e.g., an Imam or Pastor) was involved or where religious texts or doctrine were factored into the intervention (e.g., Razali, Aminah, & Khan, 2002; Rosmarin, Pargament, Pirutinsky, & Mahoney, 2010; Ward & Brown, 2015).

**Acceptability and suitability (common factors element):** Eighty-one studies (92%) reported adaptations made to improve how acceptable and suitable interventions would be for the target population. These included making adaptations to treatment structure, such as flexibility in presentation of content or addition of treatment modules (e.g., Kohn, Oden, Muñoz, Robinson, & Leavitt, 2002; Naeem et al., 2015; Neuner et al., 2008). In addition, some studies included the provision of an education component to the intervention as an adaptation (e.g., Kayrouz et al., 2015; Kruse, Joksimovic, Cavka, Woller, & Schmitz, 2009). The inclusion of components to support patient preparedness for treatment were also reported in some studies, especially where the target population might be unfamiliar with the treatment approach and concepts (e.g., Kaltman, de Mendoza, Serrano, & Gonzales, 2016; Kayrouz et al., 2015).

##### 3.1.1.2 Organisation-specific adaptations

Of the 88 studies, 54 (61%) reported adaptations that were organisation-specific.

**Location of treatment:** Nineteen studies (22%) reported changes to where an intervention would ordinarily take place, including providing the intervention at a person's home, in the community or other, non-healthcare settings (e.g., Leiler et al., 2020; Ryan et al., 2018; Scogin et al., 2007).

**Form used to provide treatment:** Thirty-eight studies (43%) reported changes to the form in which treatment was provided including where an intervention was provided digitally instead of face-to-face (or vice versa) or over the telephone (e.g., Alegria et al., 2014; Dahne et al., 2019). Some studies also adapted treatment to be group-based rather than provided on a one-to-one basis (e.g., Bolton et al., 2003; Comas-Díaz, 1981; Ward & Brown, 2015).

**Time or length of intervention:** Twenty studies (23%) reported making intentional variations in the length of the overall intervention programme (i.e., reducing or increasing the number of sessions), to the length individual sessions, or changing the time of day that an intervention was provided (e.g., Drozdek, Kamperman, Bolwerk, Tol, & Kleber, 2012; Grote et al., 2009; Meffert et al., 2014).

**Method of access:** There were 10 studies (11%) that reported adaptations to the means of access including the provision of unconventional access methods, or rapid or accelerated access to an intervention, or modified the access route (e.g., the referral route) (e.g., Meffert et al., 2014; Piedra & Byoun, 2011; Ryan et al., 2018).

Characteristics of each of the primary studies are provided in **Appendix, Table C.1** including the adapted intervention, target population and condition. **Appendix, Table E.1** details the adaptations reported for each.

#### 3.1.2 BME populations

Studies included a broad range of BME populations which were categorised into 8 groups, this included a group for studies that focussed on religious minorities. (see **Appendix**, **Table F.1**) Studies most frequently targeted Latinx people (*K* = 24) followed by East Asians (*K* = 21), refugees or asylum seekers (*K* = 19) and Black or mixed-race groups (*K* = 13).

#### 3.1.3 Mental health problems and interventions

Studies covered a range of mental health problems which were grouped as set out in Table 2**.** Several studies tested transdiagnostic interventions aimed to achieve benefits regardless of the underlying condition/diagnosis, such that in several cases, a range of outcome measures were taken covering symptoms of different mental health conditions or problems. Five studies reported more than one primary mental health outcome measure (de Graaff et al., 2020; Gonyea et al., 2016; Hendriks et al., 2020; Kaltman et al., 2016; Kayrouz et al., 2015). Overall, the most frequently assessed condition was depression (*K* = 51, 58%) and the most frequently adapted interventions were those based on cognitive behavioral therapy (CBT). See **Appendix, Table C.1** for information on interventions/treatment types upon which adaptations were made.

**Table 2:** Mental health problems by group.

Disorder label	Disorders/symptoms reported in studies and included in label
Depression	Depression; major depressive disorder; peri/post-natal depression; depressive symptoms
Anxiety disorders	Anxiety; anxiety symptoms; generalised anxiety disorder (GAD); panic disorder; panic symptoms; panic attack; phobia
Post-traumatic stress disorder (PTSD)	PTSD; PTSD symptoms; trauma; trauma symptoms
Psychosis	Psychosis; first-episode psychosis; schizophrenia; psychotic symptoms (including positive symptoms; negative symptoms)
Eating disorder	Binge eating disorder
Mental health problem NOS	Mental health problem(s) NOS; first-episode unspecified mental health problem; general mental health; psychological distress

NOS = not otherwise specified, unspecified mental health problems or assessed using general mental health or functioning measures.

### 3.2 Research question 2: effectiveness of adaptations

#### 3.2.1 Meta-analysis: symptom severity

**Fig. 3:**
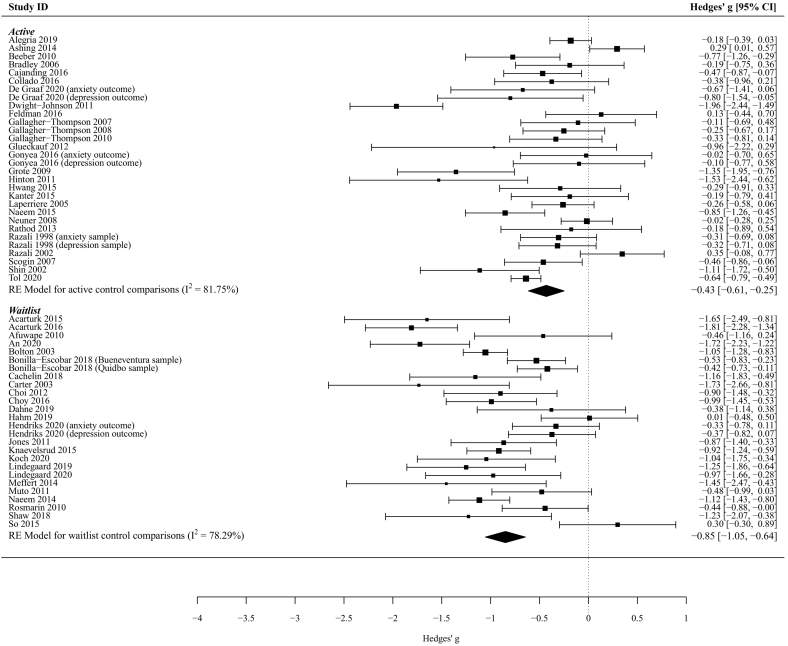
Forest plot demonstrating combined Hedge's g effect size for adapted interventions compared to active and waitlist controls.

Fifty-seven RCTs were included in the meta-analysis for which a medium effect size on symptom severity was found (*K* = 57 (62 comparisons), Hedge's *g* = -0.78 [95% CI: -0.97 to -0.60], *p* < .001). Heterogeneity was considerable: *I*^2^ = 89.95%. Six of the studies were observed to have extremely large effect sizes (Chien, Leung, & Sk Chu, 2012; Habib, Dawood, Kingdon, & Naeem, 2015; Hinton et al., 2004; Hinton et al., 2005; Kananian et al., 2020; Kruse et al., 2009) compared to other similar studies (i.e. greater than 2) and so the authors made a decision to remove these studies from the analysis, leaving 51 RCTs. This resulted in a medium effect size in favour of adapted interventions in reducing symptom severity when compared to controls across all target conditions and adaptation types (*K* *=* 51 (56 comparisons), Hedge's *g =* -0.63 [95% CI: -0.77, -0.48], *p* < .001). Heterogeneity decreased but remained substantial: *I*^2^ = 83.38% (Table 3, Fig. 3). There were few instances where adapted interventions were not associated with beneficial clinical outcomes.

**Table 3:** Sub-group analyses by control type (K = 51 [56 comparisons]).

Control type	K (Number of comparisons)	Hedge's g (95% CI)	*p*-value	*I*^*2*^
Waitlist/no intervention	21 (26)	-0.85 (-1.05, -0.64)	<0.001	78.29%
Active	30 (33)	-0.43 (-0.61, -0.25)	<0.001	81.75%

In sub-group analyses (Table 3) a large effect size was found for studies which compared adapted interventions to waitlist/no intervention controls (*K* = 21 (26 comparisons), Hedge's *g* = -0.85 [95% CI: -1.05, -0.64], *p* < .001) compared to those that used an active control group (*K* = 30 (33 comparisons), Hedge's *g* = -0.43 [95% CI: -0.61, -0.25], *p* < .001). The result of Cochran's Q for sub-group differences was significant (*p* = .003; *I*^*2*^ = 88.71%).

#### 3.2.2 Adaptation types

##### 3.2.2.1 Therapist-related, content-related and organisation-specific areas

The majority of the RCTs included in the analysis to address Research Questions 2 and 3 (*K* = 51) made adaptations to interventions covering more than one of the following areas: therapist-related, content-related or organisation-specific adaptations (Table 1; Table 4). Details of adaptations made in each study are provided in the **Appendix, Table E.1**.

**Table 4:** The number of RCTs in the main analysis (K = 51) that made adaptations to different areas.

Overarching adaptation area		
Treatment-specific: therapist-related (*K*)	Treatment-specific: content-related (*K*)	Organisation-specific (*K*)
32	47	32
**Adaptations made within overarching areas**		
In 3/3 areas (*K*)	In 2/3 areas (*K*)	In 1/3 areas (*K*)
19	22	10
**Common factors within the overarching areas**		
Therapist-related: therapeutic relationship (*K*)	Content-related: acceptability and appropriateness (*K*)	Including cultural adaptations (*K*) 43
21	48	Excluding cultural adaptations (*K*)
		5

Analyses indicate that adaptations made to any of the overarching areas appear to be effective in reducing symptom severity (Table 5). However, many of the studies included adaptations made to more than one overarching area, making ascertaining whether adaptations to certain areas are more effective than others difficult.

**Table 5:** Sub-group analyses by control type (waitlist/no intervention and active) for overarching adaptation areas (all mental health problems, all populations).

Overarching adaptation area	Control type	K (Number of comparisons)	Hedge's g (95% CI)	p-value	*I*^2^
Treatment-specific: therapist-related	Waitlist/no intervention	15 (17)	-0.80 (-1.09, -0.51)	<0.001	84.50%
	Active	17 (18)	-0.41 (-0.67, -0.15)	0.002	87.05%
Treatment-specific: content-related	Waitlist/no intervention	22 (23)	-0.90 (-1.13, -0.66)	<0.001	79.63%
	Active	25 (28)	-0.44 (-0.63, -0.25)	<0.001	81.82%
Organisation-specific	Waitlist/no intervention	17 (19)	-0.98 (-1.21, -0.74)	<0.001	74.05%
	Active	15 (17)	-0.52 (-0.78, -0.28)	<0.001	82.23%

To explore the potential importance of organisation-specific adaptations (i.e., those at the organisational or service level), studies that reported organisation-specific adaptations were compared to those that did not, using sub-group analysis. Thirty-two studies included organisation-specific adaptations. The effect size for these studies was in the medium range (*K* = 32 (36 comparisons), Hedge's *g* = -0.76, [95% CI: -0.94, -0.58], *p* ≤0.001)) and the difference between studies with organisation-specific adaptations and those without, was significant (*p* = .01; *I*^*2*^ = 84.72%). For the 19 studies without organisational adaptations, there was an effect, but this was small (*K* = 19 (20 comparisons); Hedge's *g* = -0.39 [95% CI: -0.61, -0.17], *p* = .001). Interventions which incorporated organisational adaptations were associated with significantly larger effect sizes than those without. In contrast, significant differences were not observed when comparisons were made between studies that incorporated content-related adaptations and those that did not (*p* = .06, *I*^2^ = 70.27%), or between studies that incorporated therapist-related adaptations and those that did not (*p* =. 68, *I*^2^ = 0.00%).

##### 3.2.2.2 Specific adaptation types

The most frequently reported specific adaptation type among RCTs was explicit cultural adaptation to treatment content, with 47 out of 51 (92%) studies making explicit reference to incorporating cultural adaptations (see Table 1). This was followed by reports of language translation adaptations, with 18 studies (35%) making content-related language translation adaptations and 25 (49%) making therapist-related language translation adaptations. Adaptations to the form used to provide treatment (an organisation-specific adaptation) were reported in 20 studies (39%). Other specific adaptations reported included training for the provider/facilitator (16 studies; 31%), changing the provider/facilitator (14 studies; 27%), the location of treatment (12 studies; 24%), time or length of treatment (9 studies; 18%), religious/faith-based adaptations (7 studies; 14%) and changes to the method of access (5 studies; 10%).

Large effect sizes were observed where studies included an organisation-specific adaptation to the time or length of the intervention (*K* = 9 (10 comparisons), Hedge's *g* = -1.13 [95% CI: -1.56, -0.70], *p* < .001; *I*^*2*^ = 80.74%) and this was the case both when interventions were compared to waitlist/no intervention and active controls (Table 6). Other organisation-specific adaptations (form used to provide treatment; location of treatment; method of access) also produced significantly favourable effects over controls.

**Table 6:** Meta- analyses for most frequently occurring specific adaptation types (all disorders, all BME groups).

Specific adaptation type	Control type	K (Number of comparisons)	Hedge's g (95% CI)	p-value	*I*^2^
**Treatment-specific: therapist related**					
Language translation	Waitlist/no intervention	9 (10)	-0.82 (-1.25, -0.40)	<0.001	87.71%
	Active	16 (18)	-0.47 (-0.73, -0.20)	<0.001	85.37%
Training for provider/facilitator	Waitlist/no intervention	6 (8)	-0.59 (-0.78, -0.40)	<0.001	35.72%
	Active	10 (11)	-0.29 (-0.52, -0.06)	0.015	77.27%
Provider of treatment	Waitlist/no intervention	8 (9)	-0.76 (-1.08, -0.44)	<0.001	76.20%
	Active	6 (7)	-0.74 (-1.20, -0.29)	<0.001	90.80%
**Treatment-specific: content related**					
Explicit cultural	Waitlist/no intervention	20 (21)	-0.91 (-1.15, -0.67)	<0.001	79.63%
	Active	23 (26)	-0.44 (-0.64, -0.23)	<0.001	80.47%
Language translation	Waitlist/no intervention	10 (10)	-0.69 (-1.03, -0.34)	<0.001	71.18%
	Active	8 (8)	-0.63 (-1.06, -0.19)	0.005	88.00%
Religious/faith-based	Waitlist/no intervention	4 (5)	-0.67 (-0.98, -0.34)	<0.001	71.42%
	Active	3 (4)	-0.29 (-0.76, 0.19)	0.238	81.83%
**Organisation-specific**					
Form used to provide treatment	Waitlist/no intervention	10 (10)	-1.11 (-1.31, -0.91)	<0.001	24.32%
	Active	10 (10)	-0.45 (-0.83, -0.07)	0.019	92.28%
Location of treatment	Waitlist/no intervention	4 (5)	-0.80 (-1.11, -0.48)	<0.001	73.20%
	Active	8 (9)	-0.58 (-1.02, -0.14)	0.010	87.70%
Time/length of treatment	Waitlist/no intervention	6 (7)	-1.09 (-1.58, -0.61)	<0.001	76.80%
	Active	3 (3)	-1.18 (-2.20, -0.16)	0.023	90.16%
Method of access	Waitlist/no intervention	2 (2)	-0.88 (-1.65, -0.11)	0.026	63.93%
	Active	3 (3)	-0.93 (-1.96, -0.09)	0.074	94.99%

All treatment-specific: therapist-related adaptations produced small to medium effects, as did treatment-specific: content-related adaptations. The results of the analyses are provided in Table 6. As with the overarching areas, ascertaining which specific adaptations may be more effective is difficult given that most of the studies incorporated a number of different adaptations.

##### 3.2.2.3 Common factors

###### 3.2.2.3.1 Therapeutic relationship.

Twenty-one of the RCTs reported adaptations intended to address the therapeutic relationship between participants and the person providing the intervention (see Table 1). A significant medium effect size was observed for these adaptations compared to controls (*K* = 21 (25 comparisons); Hedge's *g* = -0.58 [95% CI: -0.73, -0.42], *p* ≤0.001); a significant, small effect size was observed for studies comparing adapted interventions with active controls but there was considerable heterogeneity (Table 7).

**Table 7:** Meta-analyses for common factors adaptations (all mental health problems, all populations).

Common Factors	Control type	K (Number of comparisons)	Hedge's g (95% CI)	p-value	*I*^2^
**Therapist-related**					
Therapeutic relationship	Waitlist/no intervention	8 (9)	-0.89 (-1.23, -0.54)	<0.001	85.46%
	Active	13 (16)	-0.45 (-0.76, -0.13)	0.005	85.37%
**Content-related**					
Acceptability and suitability	Waitlist/no intervention	(25)	-0.83 (-1.04, -0.63)	<0.001	78.90%
	Active	(28)	-0.43 (-0.62, -0.23)	<0.001	83.26%
Acceptability and suitability- explicit cultural adaptations	All	43 (47)	-0.65 (-0.81, -0.48)	<0.001	83.45%
Acceptability and suitability – no explicit cultural adaptations	All	5 (6)	-0.41 (-0.64, -0.19)	0.001	66.38%

The difference between studies which made therapeutic relationship adaptations and those that did not was assessed using sub-group analysis. As above, there was a medium effect size for those that made therapeutic relationship adaptations. The effect size was also medium for studies that did not report therapeutic relationship adaptations (*K* = 30 (31 comparisons); Hedge's *g* = -0.63 [95% CI: -0.81, -0.46] *p* < .001; *I*^2^ = 76.72%) and there was no significant difference between these sub-groups (*p* = .897; *I*^2^ = 0.00%).

####### 3.2.2.3.1.1 Sensitivity analysis

There were 9 studies of self-help or self-administered interventions for which therapeutic relationship adaptations were not possible or appropriate (Cachelin et al., 2018; Choi et al., 2012; Dahne et al., 2019; Gallagher-Thompson et al., 2010; Lindegaard et al., 2020; Muto, Hayes, & Jeffcoat, 2011; Naeem et al., 2014; Rosmarin et al., 2010; Tol et al., 2020). A sensitivity analysis was run removing these 9 studies and found that the effect size was reduced but remained medium for studies without any therapeutic relationship adaptations (*K* = 21 (22 comparisons); Hedge's *g* = 0.61 [95% CI: -0.85, -0.37], *p* < .001) and the difference between sub-groups remained non-significant (*p* = .987; *I*^*2*^ = 0.00%).

A further sub-group analysis of the 9 studies reporting adapted self-help interventions produced a significant medium effect size (*K* = 9; Hedge's *g* = 0.71 [95% CI: -0.92, -0.50], *p* < .001; *I*^*2*^ = 51.27%). Seven of the studies compared adapted self-help interventions to waitlist/no intervention controls (*K* = 7; Hedge's *g* = -0.79 [95% CI: -1.06, -0.53], *p* < .001; *I*^*2*^ = 43.16) and only 2 made comparisons with active controls (*K* = 2; Hedge's *g* *=* -0.57 [95% CI: -0.82, -0.32], *p* < .001; *I*^*2*^ = 31.42). While none of these studies included therapeutic relationship adaptations, all included adaptations in line with acceptability and suitability, of which 7 (78%) were explicit cultural adaptations.

###### 3.2.2.3.2 Acceptability and suitability

The majority of RCTs (*K* = 48, 94%) reported adaptations made in line with acceptability and suitability common factors (see Table 1). Acceptability and suitability adaptations showed beneficial effects compared to controls (Table 7**;** *K* = 48 (53 comparisons); Hedge's *g* = -0.62 [95% CI: -0.77, -0.47], *p* < .001) yielding a medium effect size. A significant, small effect size was observed for studies comparing adapted interventions with active controls but there was considerable heterogeneity (Table 7).

The majority of studies reported explicit cultural adaptations to intervention content (*K* = 43). Only 5 studies were of interventions which reported adaptations to acceptability and suitability common factors (such as modifications to treatment structure, provision of education or preparation of the patient) where there was not an explicit cultural adaptation to content reported (Afuwape et al., 2010; Bonilla-Escobar et al., 2018; Dahne et al., 2019; Neuner et al., 2008; Tol et al., 2020). When analysed using sub-group analysis, a small effect size was observed for these studies (*K* = 5 (6 comparisons); Hedge's *g* = -0.41 [95% CI: -0.64, -0.19]; *p* < .001) (Table 7). There was no significant difference between these studies and those which did include explicit cultural common factors adaptations (*p* = .104, *I*^2^ = 61.99%).

#### 3.2.3 Adapted CBT interventions for depression and anxiety disorders

A number of studies looked at interventions that were adapted versions of, or included adaptations to interventions based on cognitive behavioral therapy (CBT). Given that CBT is frequently used in the treatment of common mental health disorders (CMHDs: anxiety and depression), studies of adapted CBT-based interventions for anxiety and depression were examined in a sub-group analysis.

**Fig. 4:**
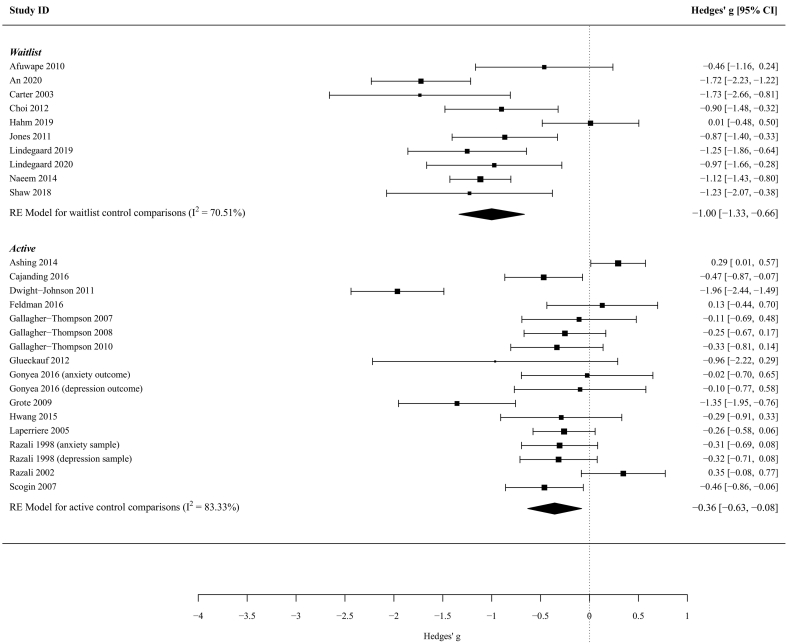
Forest plot of the subsample of studies which adapted CBT interventions for depression and anxiety symptoms.

Almost half of the studies (*K* = 25, 49%) looked at the effectiveness of adapted CBT interventions on anxiety or depression outcomes. A small but significant effect size was observed for studies which compared adapted CBT interventions to active controls, but there was considerable heterogeneity (Table 8; Fig. 4).

**Table 8:** Sub-group analyses of studies of adapted CBT-based interventions on anxiety and depression outcomes.

Control type	K (Number of comparisons)	Hedge's g (95% CI)	p-value	*I*^2^
Waitlist/no intervention	10 (10)	-1.00 (-1.33, -067)	<0.001	70.51%
Active	15 (17)	-0.36 (-0.63, -0.08)	0.012	83.33%

#### 3.2.4 Meta-regressions

Meta-regressions were performed using all RCTs (*K* = 51) to explore heterogeneity and examine the degree to which different factors may be associated with effects (including target mental health problem/symptoms, control type, overarching adaptation area, specific adaptation type, common factors adaptations and risk of bias). Control type: waitlist/no intervention, was significantly associated with effect size (β = -0.41, 95% CI: -0.67, -0.16, *p* = .002). PTSD was the only mental health problem found to be significantly associated with increased effectiveness (β = -0.64, 95% CI: -1.24, -0.05, *p* = .033) and remained as such when controlling for control type (β = -0.56, 95% CI: -0.1.11, -0.00, *p* = .049). Organisation-specific adaptation was the only overarching adaptation area significantly associated with increased effectiveness (β = -0.37, 95% CI: -0.65, -0.09, *p* = .009). Further details of meta-regressions are in **Appendix F**.

Meta-regressions were also performed using the subsample of studies of adapted CBT interventions for depression and anxiety detailed above (*K* = 27*)*. Heterogeneity and the degree to which overarching adaptation area might be associated with effects, was explored. Inclusion of organisation-specific adaptations was significantly associated with increased effectiveness in this subsample (*p* *=* .024), as was control type: waitlist/no intervention (*p* *=* .004). Neither treatment specific: therapist-related or treatment-specific: content-related adaptations were significantly associated with increased effectiveness (Table 9). Inclusion of organisation-specific adaptations remained significantly associated with intervention effect even when controlling for control type (Table 10).

**Table 9:** Single predictor meta-regressions – control type and therapist-related, content-related and organisation-specific level adaptions of CBT-based interventions for anxiety and depression symptoms.

K	Variable	Coefficient	SE	p-value	95% CI	R^2^
27	Treatment specific: therapist-related ^a^	-0.1034	0.2548	0.685	-1.03, -0.27	0.00%
27	Treatment specific: content related ^b^	0.0710	0.5508	0.897	-1.01, 1.15	0.00%
27	Organisation-specific ^c^	-0.5416	0.2407	0.024	-1.01, -0.07	10.99%
27	Waitlist/no intervention ^d^	-0.6476	0.2264	0.004	-1.09, -0.20	29.02%

^a^ reference category = no therapist-related adaptation; ^b^ reference category = no content-related adaptation; ^c^ reference category = no organisation-specific adaptation; ^d^ reference category = active control.

**Table 10:** Organisation-specific adaptations meta-regressions -CBT-based interventions for anxiety and depression symptoms.

K	Model	Variable	Coefficient	SE	p-value	95% CI	R^2^
27	1	Organisation-specific ^a^	-0.4823	0.2037	0.018	-0.88, -0.08	42.42
	2	Waitlist/no intervention ^b^	-0.6033	0.2107	0.004	-1.02, -0.19	

^a^ reference category = no organisation-specific adaptation; ^b^ reference category = active control.

The results of meta-regressions exploring other factors in this subsample of studies are provided in **Appendix F**.

### 3.3 Research question 3: effects across different BME groups

Included RCTs most frequently targeted East Asian (*K* *=* 14) or Latinx communities (*K* = 12) followed by refugees/asylum seekers (*K* = 10). Six studies targeted people from one of the BME groups who also belonged to religious minority or refugees/asylum seekers groups; **Table E.1** in the **Appendix** provides detail about the adaptations made to interventions in each included study, by target population. Information about how population groups were defined is provided in the **Appendix, Table F.1.** Efficacy of adapted interventions for different target population groups was assessed via sub-group analysis (Table 11).

**Table 11:** Meta-analyses of adapted interventions for different target population sub-groups.

Target population	K (Number of comparisons)	Hedge's g (95% CI)	p-value	*I*^2^
East Asian	14 (15)	-0.43 (-0.71, -0.16)	0.002	78.55%
Latinx	12 (13)	-0.48 (-0.84, -0.11)	0.011	86.92%
Refugees or asylum seekers	10 (11)	-0.99 (-1.35, -0.64)	<0.001	81.98%
Black or mixed race	8 (10)	-0.70 (-0.95, -0.46)	<0.001	62.59%
NOS/mixed groups/’immigrants/migrants’	5 (5)	-0.62 (-1.04, -0.19)	0.004	71.14%
Religious minority	3 (4)	-0.18 (-0.52, -0.16)	0.291	64.14%
Middle Eastern	3 (3)	-1.02 (-1.29, -0.74)	<0.001	0.00%
South Asian	2 (2)	-1.02 (-1.27, -0.77)	<0.001	2.05%

‘NOS’ = not otherwise specified.

Large effect sizes were observed for studies of Middle Eastern groups (*K* = 3; Hedge's *g* = -1.02 [95% CI: -1.29, -0.74], *p* < .001) and no heterogeneity was observed (*I*^*2*^ = 0.00%), and for 2 studies of South Asians (K = *2*; Hedge's *g* = -1.02 [95% CI: -1.27, -0.77], *p* < .001) with minimal heterogeneity (*I*^*2*^ = 2.05%). A large effect size was also observed for studies targeting refugees or asylum seekers (*K* = 10; Hedge's *g* = -0.99 [95% CI: -1.35, -0.64], *p* < .001) but there was considerable heterogeneity (*I*^*2*^ = 81.98%). The 3 studies of religious minorities did not produce significant benefits. Further details on the sub-group analyses performed by population group are included in **Appendix F**.

## 4 Discussion

The typology was developed based on a broad range of studies of cultural adaptations to psychological interventions, drawing on studies of common factors shared across psychological therapies (Wampold, 2015), the approach used to develop competence frameworks for psychological interventions (Roth & Pilling, 2018), existing cultural adaptation approaches (Barrera et al., 2013; Bernal et al., 2009; Tseng, 1999), and service-level design and delivery factors. This typology allowed for the categorisation of adaptations as treatment-specific (therapist-related and content-related) and organisation-specific, helped to identify the frequency of adaptation types, and informed the analyses undertaken in this review.

It was common for studies to report adaptations made across multiple areas (in 80% of cases). Most studies made adaptations to the treatment or intervention itself: either content-related adaptations or therapist-related adaptations, and many incorporated adaptations that focused on the therapeutic relationship. Almost 90% of studies reporting modifications or changes to better support people from minority groups made explicit reference to cultural adaptations to content and a number also made additional, specific adaptations to or for the therapist, such as providing training for the person providing treatment or changing the provider or facilitator. Approximately 60% of studies reported adaptations at the organisational level including adapting the form used to provide interventions, the time or length of the intervention, the location of treatment provision, and the method of access. From the meta-analyses of the RCTs it was evident that adapted interventions are associated with greater symptom improvements for BME groups post-treatment, compared to non-adapted interventions. Effects were greater when adapted interventions were compared to waitlist/no intervention controls but there were also significant effects of adapted interventions compared to other active treatments. This suggests that adapted interventions can provide additional benefit over and above non-adapted interventions. These benefits were also seen in self-help interventions.

Culturally-adapted interventions have consistently been found to have benefits over non-adapted interventions (e.g. Chowdhary et al., 2014; Griner & Smith, 2006) and this is likely due to the fact that adaptations allow for the provision of evidence-based treatments known to be effective, whilst ensuring that they are accessible and acceptable to people with differing cultural needs, and that there is an opportunity for development of the trust and respect that underpins the therapeutic alliance (Wampold, 2015; Wampold & Budge, 2012). The findings of this review align with those from other research into adapted care and emphasise the importance of cultural adaptation of interventions and the necessity that services carefully consider the systematic modification of treatments to ensure their compatibility with different cultural values, patterns, behaviours and beliefs. Cultural adaptations appear to be essential in ensuring that interventions can be accessed, received and implemented universally. This is of particular importance as communities continue to diversify and as services commit to ensuring equality of healthcare in multicultural societies.

Most studies in the current review included adaptations across a number of areas of the typology, so it was not possible to assess the differential benefits of adaptation types. However, comparisons between interventions that did or did not include certain adaptations were possible. Interventions that included organisation-specific adaptations were found to be more efficacious than interventions that did not in incorporate these types of adaptions. This highlights the potential value of organisation-specific adaptations and their potential benefits for BME groups, and suggests that even when other adaptation types are incorporated, there may be an additive benefit to including culturally-informed, organisation-specific adaptations when making modifications to treatment for minority groups. The common factors model does not give much consideration to the impact of external environmental factors including those at the organisational level, on intervention efficacy, although these factors are considered in some competence frameworks. Some authors have made reference to the ‘healing setting’ in the context of therapist-related common factors rather than at the organisational level (Amole et al., 2017; DeRubeis et al., 2005), which fits with the notion that the therapeutic relationship is considered vital to treatment outcomes (Stamoulos et al., 2016). Organisational factors might be especially important in improving outcomes from adaptations made to meet the needs of minority groups who often struggle to access appropriate services (Guha, 2019). In light of the findings here, it may be the case that a range of different adaptations could impact the therapeutic relationship and be beneficial, but that services seeking to improve outcomes for BME groups might pay particular attention to adapting organisation-specific elements of treatment.

Specifically, those seeking to maximise intervention efficacy might consider including organisation-specific adaptations such as changing the time or length of the intervention - adaptations found in this review to yield important effects. Making variations to the length of the overall intervention programme by reducing or increasing the number of sessions, modifying the time or length of individual sessions, or changing the time of day a treatment is offered could have considerable benefits for some patients. When considering pathways to care and accessibility, there may be little use in having available an intervention that incorporates treatment-specific adaptations that ensure it is acceptable in terms of therapeutic delivery and content, if the treatment cannot be accessed or if there are barriers to attendance. Whilst in this review, only method of access cultural adaptations were identified, there is evidence from large clinical cohorts suggesting that reducing the duration of time between referral and starting treatment is associated with improved outcomes from psychological therapies (Clark et al., 2018). It is possible that more timely access to treatment may be particularly important for those groups of people who are least likely to access care, such is the case for some minority groups in a number of settings. Services might explore these ideas with communities when seeking to develop and improve care provision, to determine what would be suitable to meet local need. Involving patients and their families as well as community leaders (Arundell et al., 2020; Lwembe, Green, Chigwende, Ojwang, & Dennis, 2017) in the development of improved access to services, might increase the effectiveness of such interventions. Implementation of organisation-specific adaptations requires careful planning at the service level, but appears worthwhile if such adaptations can lead to benefits for patients.

Whilst including organisation-specific adaptations appears to be particularly effective, many studies emphasised gaining cultural understanding and using that to develop both therapist-related and content-related adaptations. It would seem appropriate that as well as making adaptations at the organisational level, the principles underpinning the development and implementation of cultural adaptations, such as a commitment to cultural awareness and understanding the needs of different communities, should be part of any psychological therapist training programme (Council of National Psychological Associations & for the Advancement of Ethnic Minority Interests, 2003; Department of Health, 2009). Future studies should consider culturally-informed organisational, and service-level issues alongside therapy-related and content-related adaptations (Castro, Barrera, & Martinez, 2004; Pineros-Leano et al., 2017). A failure to consider the impact of organisational and service issues may result in missed opportunities to improve care.

Adapted interventions were effective across all minority groups assessed, except religious minorities although numbers here were small and the results should be treated with caution. There were encouraging results for Middle Eastern and South Asian people, although again this was based on a limited number of studies. Given the concerns that have been raised about the care received by refugees and asylum seekers (Satinsky, Fuhr, Woodward, Sondorp, & Roberts, 2019; Vostanis, 2014), it is particularly reassuring that interventions for these groups appear to be effective. The most frequent minority groups for which interventions were adapted were East Asian, and Latinx people, with fewer studies for South Asians, religious minorities, or people from the Middle East. A more robust assessment of adapted interventions for particular subgroups would require greater consistency of reporting on the nature of the adaptations, and crucially how they were developed. Future studies might use the typology in this paper, and an implementation framework that should clearly describe the interventions and the processes by which they were designed (May et al., 2007; Moullin, Sabater-Hernández, Fernandez-Llimos, & Benrimoj, 2015).

Adapted CBT interventions for depression and anxiety symptoms were efficacious in reducing symptom severity. However, few of the active control conditions provided a direct comparison of an adapted CBT intervention to its non-adapted original; a commonly reported issue with adapted interventions research (Alvidrez et al., 2019). Further comparisons of adapted and non-adapted CBT interventions should be undertaken, along with evaluations of the impact of organisation-specific adaptations on CBT outcomes. More broadly the impact of adaptations on a different ethnic groups and the potential benefit of different treatment type should also be considered.

The extensive and varied nature of the adaptations explored in this review suggest that the typology could be of considerable value in furthering understanding of the benefits associated with particular adaptations, both treatment- and organisation-focused.

## 5 Strengths and limitations

This review has a number of strengths. Inclusion criteria were broad, encompassing a range of mental health problems, interventions, adaptation types and minority populations. Studies covered a broad geographical range and were not excluded on the basis of language. By using a range of study designs to develop typology, a fuller picture of the adaptations literature was made possible. The typology of adaptations is novel and was informative for guiding the meta-analyses of intervention outcomes. The analyses provided evidence of a number of potentially informative effects, including the observation that organisation-specific adaptations were associated with improved clinical outcomes which may be additive to the impact of other types of adaptations. Although evidence for the efficacy of cultural adaptations to psychological interventions for minority groups has been previously established (Chowdhary et al., 2014; Escobar & Gorey, 2018; Hall, Ibaraki, Huang, Marti, & Stice, 2016; Van Loon et al., 2013), the present review outlined a novel way of investigating adaptations, highlighting some of the challenges with existing categorisations.

However, the reliability and validity of the conceptual typology has not been independently assessed or evaluated. Further work could lead to a more refined typology which should be informed not only by treatment developers and providers but also by the people in receipt of the services. The typology intended to provide a clearer and more useable approach to understanding adaptations, yet the concise nature of it might be considered an oversimplification of culturally-appropriate care. A further limitation is that although the typology was broad in scope and categorisation of adaptations incorporated common factors, it is possible that some adaptations may have been missed.

In addition, the review relied on definitions of ethnicity and participant background given by the included studies, with some minority groups collapsed together to investigate effects at the group level. This may have overlooked a number of potentially important distinctions between cultures, experiences and beliefs, giving rise to the potential for ecological bias. This, along with the challenges in developing the typology referred to above could have contributed to the considerable heterogeneity observed in some meta-analyses. The absence from the review of some ethnic groups may also limit the generalisability of the present findings. In particular, there were few studies of ‘indigenous’ or ‘native’ communities indicating either a paucity of studies of culturally adapted psychological interventions for such populations, or a limit to the search strategy used in this review. In addition, despite the intention to do so (as outlined in the review protocol), differences between different types of psychological intervention received (e.g., cognitive-behavior therapy versus interpersonal psychotherapy) were not able to be explored, due to a lack of available data in the eligible studies.

## 6 Conclusions

Cultural adaptations made to psychological interventions for BME groups with mental health problems appear to be efficacious relative to non-adapted or waitlist/no intervention comparators. The typology developed to assess adaptations in terms of therapist-related, content-related and organisation-specific types, may help to inform future adaptations to psychological interventions and their evaluation. One implication of the review is that in addition to a focus on therapy-related and content-related aspects of an intervention, studies should also consider culturally-informed organisational, and service-level issues and that a failure to do so may result in missed opportunities to improve care.

The typology could be used to further discussions around cultural competence training for practitioners, particularly given that its construction drew upon existing work on competence frameworks for psychological interventions which had been developed to support psychological therapy training programmes. Studies often incorporated adaptations across multiple domains and this perhaps contributed to the fact that the review was not able to find evidence for the superiority of any one adaptation type over another, although organisation-specific adaptations may be associated with additional benefits. The incorporation of a range of adaptation types is commonplace across adapted interventions studies and is perhaps indicative of the complex nature of the task. The typology provides a possible means to address this complexity. It applies the distinction between cultural adaptations to provision and content of interventions and culturally-informed adaptations to other organisational factors reflecting the interface between cultural knowledge and existing service structures. Treatment developers and evaluators, service providers, and service users seeking to improve interventions and treatment outcomes for BME groups might use the typology as a tool to assess the degree to which adaptation types are effective for specific minority groups. This may contribute to efforts to improve treatment effectiveness and to aid decision-making about how to identify and apply adaptations to psychological interventions to better meet the mental health needs of BME populations.

This review identified not only differences in the definitions of culturally adapted care, but that studies themselves varied greatly in the provision of detail about how interventions were adapted. This review and the typology that was developed, offers a method by which this limitation can be addressed. Further treatment development and research is needed to improve outcomes for BME groups who suffer a disproportionate burden of mental health problems; the adoption and further development of the conceptual typology may contribute to this endeavour.

## Funding

This research did not receive any specific grant from funding agencies in the public, commercial, or not-for-profit sectors and was developed as part of the corresponding author's doctoral thesis in Clinical, Educational and Health Psychology at University College London (UCL), UK. Co-authors S Pilling, R Saunders and JEJ Buckman are supported by the 10.13039/501100000272National Institute for Health Research (NIHR) University College London Hospitals Biomedical Research Centre and, JEJ Buckman is supported by the 10.13039/100010269Wellcome Trust (Grant Code 201292/Z/16/Z). S Pilling is also the recipient of a grant from the Royal College of Psychiatrists: Psychological Processes and Clinical Effectiveness (2017 to 2022) with R Saunders. None of these funders had any role in the study design, collection, analysis or interpretation of the data, writing the manuscript, or the decision to submit the paper for publication.

## Contributors

Laura-Louise Arundell (L-LA), Professor Stephen Pilling (SP) and Dr. Rob Saunders (RS) conceptualised the study. L-LA wrote the protocol, conducted the bibliographic database searches, performed the screening exercises and study selection processes, extracted data, ran the analyses and wrote the original draft. Phoebe Barnett (PB) supported with screening exercises, study selection and data extraction. PB also supported with data visualisation and performed a review and edit of the draft. SP provided supervision, contributed to the research methodology and performed a review and edit of the draft. RS contributed to the methodology, data analysis and performed a review and edit of the draft. Dr. Joshua EJ Buckman (JEJB) contributed to the methodology and performed a review and edit of the draft. All authors have approved this manuscript.

## Declaration of Competing Interest

None declared.
