# Supplementary material for: The effectiveness of adapted psychological interventions for people from ethnic minority groups: A systematic review and conceptual typology
Source: Clin Psychol Rev. 2021 Aug;88:102063. doi: 10.1016/j.cpr.2021.102063 (PMC8591374; doi:10.1016/j.cpr.2021.102063)
Supplement: Supplementary material [file mmc1.docx]

# Appendix A: Search strategy

## **Original search: April 2019**

| **Date of search: 06.04.19** | |
| --- | --- |
| **Database (via Ovid)** | **Records retrieved** |
| Ovid MEDLINE(R) and Epub Ahead of Print, In-Process & Other Non-Indexed Citations and Daily 1946 to April 05, 2019 | 1258 |
| Embase Classic+Embase 1947 to 2019 April 05 | 1301 |
| PsycINFO 1806 to April Week 1 2019 | 441 |
| HMIC Health Management Information Consortium 1979 to January 2019 | 3 |

**MEDLINE; EMBASE; PSYCHINFO, HMIC (via Ovid)**

1. randomized controlled trial.pt.

2. controlled clinical trial.pt.

3. randomized.ab.

4. placebo.ab.

5. clinical trials as topic.sh.

6. randomly.ab.

7. trial.ti.

8. 1 or 2 or 3 or 4 or 5 or 6 or 7

9. ((systematic adj review*) or meta analy* or metaanaly*).tw.

10. meta-analysis as topic/ or Meta-Analysis.pt.

11. 9 or 10

12. Epidemiologic studies/ or exp case control studies/ or exp cohort studies/ or Cross-sectional studies/

13. Case control.tw.

14. (cohort adj (study or studies)).tw.

15. Cohort analy$.tw.

16. (observational adj (study or studies)).tw.

17. 12 or 13 or 14 or 15 or 16

18. 8 or 11 or 17

19. comment/ or editorial/ or letter/

20. 18 not 19

21. exp Ethnic Groups/

22. exp Minority Groups/

23. ("BME" or "BAME" or "black asian and minority ethnic" or "black and minority ethnic" or "ethnic minorit*" or "asian minorit*").tw.

24. Asian Continental Ancestry Group/ or African Continental Ancestry Group/ or European Continental Ancestry Group/ or African Americans/ or Asian Americans/ or Hispanic Americans/

25. gypsies/

26. Roma/

27. (traveller$1 or Gypsies or Gypsy or Gipsy or Gipsies or Romany or Romanies or Romani or Romanis or Rromani or Rromanis or Roma).ti,ab.

28. "Transients and Migrants"/

29. "Emigration and Immigration"/

30. "Emigrants and Immigrants"/

31. refugees/

32. (immigrant$ or migrant$ or asylum or refugee$ or undocumented).ti,ab.

33. (displaced and (people or person$1)).ti,ab.

34. (born adj2 overseas).ti,ab.

35. exp Psychotherapy/

36. ("mental health care" or "mental healthcare" or "mental health intervention*" or "mental health treatment" or "mental health support" or "psychological therap*" or "psychological intervention" or "psychology intervention" or "psychological treatment" or "psychology support" or "psychological support").tw.

37. 21 or 22 or 23 or 24 or 25 or 26 or 27 or 28 or 29 or 30 or 31 or 32 or 33 or 34

38. 35 or 36

39. 20 and 37 and 38

40. limit 39 to (humans and yr="1965 -Current")

| **Date of search: 07.04.19** | |
| --- | --- |
| **Database (via ProQuest)** | **Records retrieved** |
| Assia 1 January 1965 to 6 April 2019 | 1083 |

**ASSIA (via Proquest)**

(MAINSUBJECT.EXACT.EXPLODE("Minority groups") OR MAINSUBJECT.EXACT.EXPLODE("Ethnic groups") OR TI,AB("Central Asian people") OR TI,AB("Asian Australian people") OR TI,AB("South East Asian American people") OR TI,AB("Asian American people") OR TI,AB("South East Asian people") OR TI,AB("East Asian people") OR TI,AB("Asian communities") OR TI,AB("Ugandan Asian people") OR TI,AB("Asian people") OR TI,AB("Asian-Pacific American people") OR TI,AB("South Asian people") OR TI,AB("Australasian people") OR TI,AB("Eurasian people") OR TI,AB("South Asian communities") OR TI,AB("Black Asian American people") OR TI,AB("Gypsies") OR TI,AB("Immigrants") OR TI,AB("Migrants") OR TI,AB("Emigrants") OR TI,AB("Refugees") OR TI,AB("Asylum") OR TI,AB("Immigration") OR ti,ab("BAME" OR "BME" OR "black asian and minority ethnic" OR "black and minority ethnic" OR ethnic minorit* OR asian minorit* OR minorit* OR travel?er*1 OR Gypsies OR Gypsy OR Gipsies OR Romany OR Romani OR Romanis OR Rromanis OR Roma OR immigrant*1 OR migrant*1 OR refugee*1 OR undocumented OR displaced people OR displaced person*1 OR born overseas)) AND (MAINSUBJECT.EXACT.EXPLODE("Psychotherapy") OR ti,ab(psychotherapy OR cognitive behavio?ral therapy OR "CBT" OR mental health care OR mental health OR mental healthcare OR mental health intervention OR mental health treatment OR mental health support OR psychological therap* OR psychological intervention OR psychology intervention OR psychological treatment OR psychology support OR psychological support)) AND (TI,AB("Meta-analysis") OR TI,AB("Cohort analysis") OR TI,AB("Cross-sectional studies") OR TI,AB("Observational research") OR TI,AB("Randomized controlled trials") OR TI,AB("Systematic reviews") OR ti,ab(systematic NEAR/4 review*) OR meta analy* OR metaanaly* OR randomized controlled trial OR controlled clinical trial OR randomized OR placebo OR randomly OR trial OR Case control OR (cohort NEAR/4 (study OR studies)) OR Cohort analy OR (Follow up NEAR/4 (study OR studies)) OR (observational NEAR/4 (study OR studies))) AND PEER(yes) AND pd(19650101-20190406)

| **Date of search: 07.04.19** | |
| --- | --- |
| **Database (via EBSCO)** | **Records retrieved** |
| CINAHL | 721 |

**CINAHL**

| **#** | **Query** | **Limiters/Expanders** | **Last Run Via** | **Results** |
| --- | --- | --- | --- | --- |
| S1 | MH (MH "Minority Groups+") OR (MH "Ethnic Groups+") | Search modes - Boolean/Phrase | Interface - EBSCOhost Research Databases  Search Screen - Advanced Search  Database - CINAHL Plus | 133,338 |
| S2 | MW ethnic minorities OR MW ( black, asian and minority ethnic (bame) ) OR MW ( bme or black minority ethnic or ethnic or african or caribbean ) OR MW asian american OR MW ( african americans or black americans or blacks ) OR MW ( hispanic or latino or latina or mexican or central american or south american or hispanics or latin ) OR MW ( gypsy or roma or traveller ) OR MW ( migrants or immigrants or asylum seekers or refugees ) OR MW ( emigration or immigration or migration ) OR MW emigrants OR MW displaced persons | Search modes - Boolean/Phrase | Interface - EBSCOhost Research Databases  Search Screen - Advanced Search  Database - CINAHL Plus | 109,594 |
| S3 | (MH psychotherapy+) | Search modes - Boolean/Phrase | Interface - EBSCOhost Research Databases  Search Screen - Advanced Search  Database - CINAHL Plus | 163,340 |
| S4 | MW "psychotherapy" OR "cognitive behavio#ral therapy" OR "CBT" OR "mental health care" OR "mental health" OR "mental healthcare" OR "mental health intervention" OR "mental health treatment" OR "mental health support" OR "psychological therap?" OR "psychological intervention" OR "psychology intervention" OR “psychological treatment" OR "psychology support" OR "psychological support" | Search modes - Boolean/Phrase | Interface - EBSCOhost Research Databases  Search Screen - Advanced Search  Database - CINAHL Plus | 149,052 |
| S5 | MW (randomized controlled trials or rtc or randomised control trials ) OR MW ( meta-analysis or systematic review ) OR MW cohort design study OR MW observational research | Search modes - Boolean/Phrase | Interface - EBSCOhost Research Databases  Search Screen - Advanced Search  Database - CINAHL Plus | 163,891 |
| S6 | MW “systematic review*” OR “meta analy*” OR “metaanaly*” OR “randomized controlled trial” OR “controlled clinical trial” OR “randomized” OR “placebo” OR “randomly” OR “trial” OR “Case control“ OR “cohort stud*” OR “Cohort analy*” OR “Follow up stud*” OR “observational stud*” | Search modes - Boolean/Phrase | Interface - EBSCOhost Research Databases  Search Screen - Advanced Search  Database - CINAHL Plus | 235,668 |
| S7 | S1 OR S2 | Search modes - Boolean/Phrase | Interface - EBSCOhost Research Databases  Search Screen - Advanced Search  Database - CINAHL Plus | 157,262 |
| S8 | S3 OR S4 | Search modes - Boolean/Phrase | Interface - EBSCOhost Research Databases  Search Screen - Advanced Search  Database - CINAHL Plus | 271,573 |
| S9 | S5 OR S6 | Search modes - Boolean/Phrase | Interface - EBSCOhost Research Databases  Search Screen - Advanced Search  Database - CINAHL Plus | 235,668 |
| S10 | S7 AND S8 AND S9 | Limiters - Published Date: 19650101-20190431; Human  Search modes - Boolean/Phrase | Interface - EBSCOhost Research Databases  Search Screen - Advanced Search  Database - CINAHL Plus | 721 |

| **Date of search: 29.04.19** | |
| --- | --- |
| **Database (via Wiley)** | **Records retrieved** |
| CENTRAL | 877 |

**CENTRAL (via Wiley)**

ID Search

#1 MeSH descriptor: [Ethnic Groups] explode all trees

#2 MeSH descriptor: [Minority Groups] explode all trees

#3 MeSH descriptor: [Asian Continental Ancestry Group] this term only

#4 MeSH descriptor: [African Continental Ancestry Group] this term only

#5 MeSH descriptor: [European Continental Ancestry Group] this term only

#6 MeSH descriptor: [African Americans] this term only

#7 MeSH descriptor: [Asian Americans] this term only

#8 MeSH descriptor: [Hispanic Americans] this term only

#9 MeSH descriptor: [Roma] this term only

#10 MeSH descriptor: [Transients and Migrants] this term only

#11 MeSH descriptor: [Emigration and Immigration] this term only

#12 MeSH descriptor: [Emigrants and Immigrants] this term only

#13 MeSH descriptor: [Refugees] this term only

#14 ("BAME"):ti,ab,kw OR ("BME"):ti,ab,kw OR ("black asian and minority ethnic"):ti,ab,kw OR ("black and minority ethnic"):ti,ab,kw OR ("minority group"):ti,ab,kw (Word variations have been searched) with Publication Year from 1965 to 2019, with Cochrane Library publication date Between Jan 1965 and Apr 2019, in Trials

#15 ("traveller"):ti,ab,kw OR ("travellers"):ti,ab,kw OR ("Gypsies"):ti,ab,kw OR ("Gypsy"):ti,ab,kw OR ("Romany"):ti,ab,kw (Word variations have been searched) with Publication Year from 1965 to 2019, with Cochrane Library publication date Between Jan 1965 and Apr 2019, in Trials

#16 ("displaced people"):ti,ab,kw OR ("displaced person"):ti,ab,kw (Word variations have been searched) with Publication Year from 1965 to 2019, with Cochrane Library publication date Between Jan 1965 and Apr 2019, in Trials

#17 ("refugees"):ti,ab,kw OR ("asylum"):ti,ab,kw OR ("undocumented"):ti,ab,kw OR ("immigrant"):ti,ab,kw OR ("migrant"):ti,ab,kw (Word variations have been searched) with Publication Year from 1965 to 2019, with Cochrane Library publication date Between Jan 1965 and Apr 2019, in Trials

#18 MeSH descriptor: [Psychotherapy] explode all trees

#19 ("mental health care"):ti,ab,kw OR ("mental healthcare"):ti,ab,kw OR ("mental health intervention"):ti,ab,kw OR ("mental health treatment"):ti,ab,kw OR ("mental health support"):ti,ab,kw (Word variations have been searched) with Publication Year from 1965 to 2019, with Cochrane Library publication date Between Jan 1965 and Apr 2019, in Trials

#20 ("psychology intervention"):ti,ab,kw OR ("psychological treatment"):ti,ab,kw OR ("psychological therapies"):ti,ab,kw OR ("psychology support"):ti,ab,kw OR ("psychological support"):ti,ab,kw (Word variations have been searched) with Publication Year from 1965 to 2019, with Cochrane Library publication date Between Jan 1965 and Apr 2019, in Trials

#21 ("psychological therapy"):ti,ab,kw OR ("psychological therapies"):ti,ab,kw OR ("psychological intervention"):ti,ab,kw OR ("psychology intervention"):ti,ab,kw OR ("psychological treatment"):ti,ab,kw (Word variations have been searched) with Publication Year from 1965 to 2019, with Cochrane Library publication date Between Jan 1965 and Apr 2019, in Trials

#22 #1 OR #2 OR #3 OR #4 OR #5 OR #6 OR #7 #OR #8 OR #9 OR #10 OR #11 OR #12 OR #13 OR #14 OR #15 OR #16 OR #17 with Publication Year from 1965 to 2019, with Cochrane Library publication date Between Jan 1965 and Apr 2019, in Trials

#23 #18 OR #19 OR #20 OR #21 with Publication Year from 1965 to 2019, with Cochrane Library publication date Between Jan 1965 and Apr 2019, in Trials

#24 #22 AND #23 with Publication Year from 1965 to 2019, with Cochrane Library publication date Between Jan 1965 and Apr 2019, in Trials

| **Date of search: 29.04.20** |  |
| --- | --- |
| **Database (via Wiley)** | **Records retrieved** |
| CDSR | 6 |

CDSR (via Wiley)

ID Search

#1 MeSH descriptor: [Ethnic Groups] explode all trees

#2 MeSH descriptor: [Minority Groups] explode all trees

#3 MeSH descriptor: [Asian Continental Ancestry Group] this term only

#4 MeSH descriptor: [African Continental Ancestry Group] this term only

#5 MeSH descriptor: [European Continental Ancestry Group] this term only

#6 MeSH descriptor: [African Americans] this term only

#7 MeSH descriptor: [Asian Americans] this term only

#8 MeSH descriptor: [Hispanic Americans] explode all trees

#9 MeSH descriptor: [Roma] this term only

#10 MeSH descriptor: [Transients and Migrants] this term only

#11 MeSH descriptor: [Emigration and Immigration] this term only

#12 MeSH descriptor: [Emigrants and Immigrants] explode all trees

#13 MeSH descriptor: [Refugees] explode all trees

#14 ("BAME"):ti,ab,kw OR ("BME"):ti,ab,kw OR ("black asian and minority ethnic"):ti,ab,kw OR ("black and minority ethnic"):ti,ab,kw OR ("minority group"):ti,ab,kw with Cochrane Library publication date Between Jan 1965 and Apr 2019, in Cochrane Reviews (Word variations have been searched)

#15 ("traveller"):ti,ab,kw OR ("travellers"):ti,ab,kw OR ("Gypsies"):ti,ab,kw OR ("Gypsy"):ti,ab,kw OR ("Romany"):ti,ab,kw with Cochrane Library publication date Between Jan 1965 and Apr 2019, in Cochrane Reviews (Word variations have been searched)

#16 ("displaced people"):ti,ab,kw OR ("displaced person"):ti,ab,kw with Cochrane Library publication date Between Jan 1965 and Apr 2019, in Cochrane Reviews (Word variations have been searched)

#17 ("refugees"):ti,ab,kw OR ("asylum"):ti,ab,kw OR ("undocumented"):ti,ab,kw OR ("immigrant"):ti,ab,kw OR ("migrant"):ti,ab,kw with Cochrane Library publication date Between Jan 1965 and Apr 2019, in Cochrane Reviews (Word variations have been searched)

#18 MeSH descriptor: [Psychotherapy] explode all trees

#19 ("mental health care"):ti,ab,kw OR ("mental healthcare"):ti,ab,kw OR ("mental health intervention"):ti,ab,kw OR ("mental health treatment"):ti,ab,kw OR ("mental health support"):ti,ab,kw with Cochrane Library publication date Between Jan 1965 and Apr 2019, in Cochrane Reviews (Word variations have been searched)

#20 ("psychology intervention"):ti,ab,kw OR ("psychological treatment"):ti,ab,kw OR ("psychological therapies"):ti,ab,kw OR ("psychology support"):ti,ab,kw OR ("psychological support"):ti,ab,kw with Cochrane Library publication date Between Jan 1965 and Apr 2019, in Cochrane Reviews (Word variations have been searched)

#21 ("psychological therapy"):ti,ab,kw OR ("psychological therapies"):ti,ab,kw OR ("psychological intervention"):ti,ab,kw OR ("psychology intervention"):ti,ab,kw OR ("psychological treatment"):ti,ab,kw with Cochrane Library publication date Between Jan 1965 and Apr 2019, in Cochrane Reviews (Word variations have been searched)

#22 #1 OR #2 OR #3 OR #4 OR #5 OR #6 OR #7 #OR #8 OR #9 OR #10 OR #11 OR #12 OR #13 OR #14 OR #15 OR #16 OR #17 with Cochrane Library publication date Between Jan 1965 and Apr 2019, in Cochrane Reviews (Word variations have been searched)

#23 #18 OR #19 OR #20 OR #21 with Cochrane Library publication date Between Jan 1965 and Apr 2019, in Cochrane Reviews (Word variations have been searched)

#24 #22 AND #23 with Cochrane Library publication date Between Jan 1965 and Apr 2019, in Cochrane Reviews (Word variations have been searched)

## **Update search results: June 2020**

| **Date of search: 30.06.20** | |
| --- | --- |
| **Database (via Ovid)** | **Records retrieved** |
| Ovid MEDLINE(R) and Epub Ahead of Print, In-Process & Other Non-Indexed Citations and Daily 1946 to June 29, 2020 | 112 |
| Embase Classic+Embase 1947 to 2020 June 29 | 305 |
| PsycINFO 1806 to June Week 4 2020 | 46 |
| HMIC Health Management Information Consortium 1979 to May 2020 | 5 |

| **Date of search: 30.06.20** | |
| --- | --- |
| **Database (via ProQuest)** | **Records retrieved** |
| Assia 7 April 2019 to 30 June 2020 | 86 |

| **Date of search: 30.06.20** | |
| --- | --- |
| **Database (via EBSCO)** | **Records retrieved** |
| CINAHL | 140 |

| **Date of search: 30.06.20** | |
| --- | --- |
| **Database (via Wiley)** | **Records retrieved** |
| CENTRAL | 8 |

| **Date of search: 30.06.20** |  |
| --- | --- |
| **Database (via Wiley)** | **Records retrieved** |
| CDSR | 1 |

## **Update search results: December 2020**

| **Date of search: 11.12.20** | |
| --- | --- |
| **Database (via Ovid)** | **Records retrieved** |
| Ovid MEDLINE(R) and Epub Ahead of Print, In-Process & Other Non-Indexed Citations and Daily 1946 to December 10 2020 | 45 |
| Embase Classic+Embase 1947 to 2020 December 10 | 206 |
| PsycINFO 1806 to November Week 5 2020 | 32 |
| HMIC Health Management Information Consortium 1979 to November 2020 | 0 |

| **Date of search: 11.12.20** | |
| --- | --- |
| **Database (via ProQuest)** | **Records retrieved** |
| Assia 30 June 2020 to 11 December 2020 | 47 |

| **Date of search: 11.12.20** | |
| --- | --- |
| **Database (via EBSCO)** | **Records retrieved** |
| CINAHL | 42 |

| **Date of search: 11.12.20** | |
| --- | --- |
| **Database (via Wiley)** | **Records retrieved** |
| CENTRAL | 0 |

| **Date of search: 11.12.20** |  |
| --- | --- |
| **Database (via Wiley)** | **Records retrieved** |
| CDSR | 0 |

# Appendix B: Supplementary detail of typology development

Included here is a previous iteration of the conceptual typology, where different terms were used to classify adaptations. Detail on the evolution of terms is provided, including reasons why these were re-organised and modified by the authors:

**Table B.1: A previous iteration of the conceptual typology**

|  | ***Culturally-specific*** | | ***Culturally-informed*** |
| --- | --- | --- | --- |
|  | **Therapeutic delivery** | **Content** | **Organisational** |
| **Common factors** | **Therapeutic relationship**  Alliance;  Empathy; Agreement of treatment goals; Expectations of treatment; Collection of patient feedback | **Acceptability and appropriateness**  Treatment structure; Education; Preparation of the patient  **Cultural**  Culturally relevant terms of reference; Modified materials/resources; Culturally-sensitive language; Emphasis on cultural norms/expectations;  ‘Culturally sensitive’, ‘culturally congruent’, ‘cultural emphasis’, ‘cultural attunement’, culturally-responsive’, ‘culturally tailored’ |  |
| **Specific adaptation types** | **Training for provider/facilitator**  Staff or professional; Layperson or community member |  | **Location of treatment**  Care provided at home; Care provided in the community; Care provided in non-healthcare setting |
|  | **Language translation (delivery)**  Interpreter or bilingual provider | **Language translation (content)**  Translated materials/resources | **Medium used to provide treatment**  Face to face Telephone Online or mobile app Group treatment |
|  | **Provider of treatment**  Ethnic matching; Layperson; Community leader (e.g. religious leader) | **Religious/faith-based adaptations**  Modified materials or resources; Use of religious texts, doctrine or guidance; Presence or support from a religious leader or official | **Time or length of intervention**  Extended or shortened intervention or session length;  Time of day |
|  |  |  | **Method of access**  Rapid or accelerated access; Access route (e.g. via alternative to standard route) |

Treatment-specific adaptations (to interventions provided) were originally coined ‘culturally specific’ to capture that both content and therapist (facilitator/provider) adaptations. The conceptualisation behind this was that treatment-specific adaptations were more likely to be directly influenced by an appeal to the target group’s culture. Upon peer review, it became clear that the term was confusing and did not accurately reflect our intentions, nor the adaptations to which we were intending to refer.

- Therapist-related adaptations were originally termed ‘therapeutic delivery’ adaptations. Both of these terms would be acceptable, but we wanted to make it clear that these adaptations were as a direct result of the therapist/provider/facilitator, including both their demographics, competence, training and behaviour.
- Content-related adaptations – for consistency and clarity the ‘-related’ term was added inline with the therapist-related term above.
- Organisation-specific adaptations (those at the organisation or service level) were initially coined ‘culturally-informed’ to reflect the fact that adaptations included here refer to those that are informed by cultural knowledge and its interface with service delivery and design, yet are not always made as a direct response to specific cultural needs. As with the ‘culturally-specific’ term, it became clear upon peer review that the term was confusing and did not accurately reflect our intentions.
- ‘Acceptability and appropriateness’ was re-termed ‘acceptability and suitability’ as it was thought to better distinguish between what is meant by these two terms.
- ‘Cultural’ was expanded to refer to explicit cultural adaptations in order to clarify the types of adaptations included in this category. This is further elaborated in the review, with examples.

# Appendix C: Study characteristics – primary studies

***Table C.1: study characteristics of primary studies (K=88)***

| **Study ID** | **Study design** | **Target population** | **Adapted intervention** | **EBT upon which intervention is based/adapted from** | **Target condition(s)** | | **Duration of treatment** | **Primary outcome measure(s)** | **RoB judgement** |
| --- | --- | --- | --- | --- | --- | --- | --- | --- | --- |
| (Acarturk et al., 2015) | RCT | Refugees/Asylum seekers | Eye movement desensitization reprocessing (EMDR) | Eye movement desensitization reprocessing (EMDR) | PTSD; Depression | | 7 weeks | IES-R | Unclear |
| (Acarturk et al., 2016) | RCT | Refugees/Asylum seekers | Eye movement desensitization reprocessing (EMDR) | Eye movement desensitization reprocessing (EMDR) | PTSD; Depression | | NR | HTQ | Low risk |
| (Afuwape et al., 2010) | RCT | Black or mixed race | Cares of Life: rapid access | CBT; brief solution-focussed therapy | Anxiety; Depression | | NR | GHQ-28 | -- |
| (Alavi & Hirji, 2020) | RCT | Middle Eastern | eCBT (Microsoft PowerPoint) | CBT | Anxiety | | 12 weeks | BAI | -- |
| (Alegria et al., 2014) | RCT | Latinx | Engagement and Counselling for Latinos (ECLA; telephone or face to face) | CBT; care management | Depression | | 6-8 sessions | PHQ-9 | -- |
| (Alegría et al., 2019) | RCT | Latinx | Integrated Intervention for Dual Problems and Early Action programme (IIDEA) CBT | CBT; Motivational Interviewing; Mindfulness | Mental health NOS | | 10-12 sessions | HSCL-20 | -- |
| (An, Wang, Sun, & Zhang, 2020) | RCT | East Asian | Modified CBT (group) | CBT | Anxiety | | 8 weeks | PTA scale | Low risk |
| (Ashing & Rosales, 2014) | RCT | Latinx | Telephonic intervention + survivorship booklet | CBT; Health-related quality of life intervention | Depression | | 8 sessions | CES-D | -- |
| (Bedoya et al., 2014) | RCT | Latinx | Culturally focussed psychiatric consultation (CFP) | CBT; Psychoeducation (multicomponent intervention) | Depression | | 2 sessions | QIDS-SR | -- |
| (Beeber et al., 2010) | RCT | Latinx | Advanced practice nurse-delivered, culturally tailored, in-home psychotherapy intervention | IPT | Depression | | 16 contacts/sessions | CES-D | Low risk |
| (Bernardi, Dahiya, & Jobson, 2019) | Pre-post | Refugees/asylum seekers | Modified cognitive processing therapy | Cognitive processing therapy | PTSD | | 12 sessions | PCL-5 | -- |
| (Bernstein et al., 2016) | Non-randomised quasi experimental | East Asian | Enhanced Logo-Autobiography | Logotherapy | Depression | | 8 weeks | CES-D (Korean version) | -- |
| (Bolton et al., 2003) | Cluster RCT | Black or mixed race | Group IPT | IPT | Depression | | 16 weeks | HSCL (depression subscale) | -- |
| (Bonilla-Escobar et al., 2018) | RCT | Black or mixed race | Common elements treatment approach (CETA) | CBT | Depression; PTSS; Anxiety | | 12-14 sessions | TMHS | -- |
| (Bradley et al., 2006) | RCT | East Asian | Multiple family group therapy | CBT | Schizophrenia | | 12 months (26 sessions) | BPRS | Low risk |
| (Cachelin et al., 2018) | RCT | Latinx | CBT-guided self help | CBT (CBT-guided self-help) | Eating disorder: binge eating | | 12 weeks | EDE (objective binge-eating episodes) | Low risk |
| (Cajanding, 2016) | RCT | East Asian | Nurse-led CBT | CBT | Depressive symptoms | | 12 weeks | CDS | Low risk |
| (Camacho et al., 2015) | Pre-post | Latinx | Problem solving therapy using the IMPACT model | CBT - Problem solving therapy | Depressive symptoms | | NR | PHQ-9 | -- |
| (Carter, Sbrocco, Gore, Marin, & Lewis, 2003) | RCT | Black or mixed race | Group Panic Control Therapy | CBT | Panic disorder | | 11 sessions | CSR (panic) | Low risk |
| (Chien, Leung, & Sk Chu, 2012) | RCT | East Asian | Nurse-led psychoeducation | Psychoeducation | First-episode mental illness | | 3-6 weeks (6 sessions) | BPRS | -- |
| (Choi et al., 2012) | RCT | East Asian | Internet CBT (Brighten your mood programme) | CBT (internet CBT) | Depression | | 8 weeks | BDI (Chinese version) | Unclear |
| (Choy & Lou, 2016) | RCT | East Asian | Instrumental Reminiscence Intervention -Hong Kong (IRI HK) | Reminiscence Intervention | Depressive symptoms | | 6 weeks | GDS-15 (Chinese version) | Low risk |
| (Collado, Castillo, Maero, Lejuez, & Macpherson, 2014) | Pre-post (stage 1) | Latinx | Behavioural activation (BA) | Behavioural activation (BA) | Depressive symptoms | | 10 sessions | BDI-II | -- |
| (Collado A., Calderon M., MacPherson L., & Lejuez C., 2016) | RCT | Latinx | Behavioural activation (BA) | Behavioural activation (BA) | Depression: Major Depressive Disorder | | 10 sessions | BDI-II | Low risk |
| (Comas-Díaz, 1981) | RCT | Latinx | Cognitive therapy | Cognitive therapy | Depressive symptoms | | 4 weeks | BDI | -- |
| (Cooper et al., 2013) | Cluster randomised trial | Black or mixed race | Patient-centred collaborative care | Collaborative care | Depression: Major Depressive Disorder | | NR | CES-D | -- |
| (Dahne et al., 2019) | RCT | Latinx | ¡Aptívate! (mobile app) | Behavioural activation (brief) | Depressive symptoms | | 8 weeks | BDI-II (Spanish Version) | Unclear |
| (de Graaff et al., 2020) | RCT | Refugees/Asylum seekers | Problem Management Plus | Problem Management Plus | MH NOS (transdiagnostic),  Depression & Anxiety symptoms | | 6 weeks (5 sessions) | HCSL 25-depression subscale; HCSL 25- anxiety subscale | Low risk |
| (Drozdek, Kamperman, Bolwerk, Tol, & Kleber, 2012) | Cohort study (controlled comparison) | Refugees/Asylum seekers | Group therapy: Den Bosch model | CBT; Psychodynamic therapy | PTSD | | 12 months | HTQ PTSD scale 30 | -- |
| (Dwight-Johnson et al., 2011) | RCT | Latinx | CBT (telephone) | CBT | Depression | | 8 sessions | PHQ-9 | -- |
| (Feldman et al., 2016) | RCT | Latinx | Cognitive Behaviour Psychophysiological Therapy (CBPT) | Cognitive Behaviour Psychophysiological Therapy (CBPT) | Panic Disorder | | 8 weeks | PDSS | Low risk |
| (Gallagher-Thompson et al., 2007) | RCT | East Asian | (In-home behavioural management program) IHBMP | CBT | Depression; Stress | | 12 weeks | CES-D | Unclear |
| (Gallagher-Thompson, Gray, Dupart, Jimenez, & Thompson, 2008) | RCT | Latinx | Coping with caregiving (CWC) | CBT | Depressive symptoms | | 4 months | CES-D | -- |
| (Gallagher-Thompson et al., 2010) | RCT | East Asian | DVD: CBT skill training (self-help) | CBT | Depression; Stress | | 12 weeks | CES-D | Unclear |
| (Glueckauf et al., 2012) | Mixed methods (RCT reported) | Black or mixed race | Telephone CBT | CBT | Depression | | 12 weeks | CES-D | Unclear |
| (Gonyea, Lopez, & Velasquez, 2016) | RCT | Latinx | ‘Circulo de Cuidado’ (CBT group intervention) | CBT | Depression; Anxiety | | 5 weeks (+ follow up coaching up to 12 weeks more) | CES-D (Spanish version); STAI-S (Spanish version) | Unclear |
| (Grote et al., 2009) | RCT | BME NOS | Enhanced brief IPT | IPT (brief) | Perinatal depression | | 3-6 months | EPDS | Low risk |
| (Habib, Dawood, Kingdon, & Naeem, 2015) | RCT | South Asian | Culturally adapted CBT for psychosis (CA-CBTp) | CBT (CBTp) | Schizophrenia | | 4-6 months | PANNS - general | Unclear |
| (Hahm et al., 2019) | RCT | East Asian | ‘AWARE’ | CBT; Empowerment; Mindfulness | Depression | | 8 weeks | CES-D | Low risk |
| (Hahm et al., 2020) | Pre-post | East Asian; South Asian | ‘AWARE’ | CBT; Empowerment; Mindfulness | Depression; Anxiety; PTSD | | 8 weeks | CESD-R; HADS-A; PCL-C | -- |
| (Heilemann, Pieters, Kehoe, & Yang, 2011) | Pre-post | Latinx | Motivational Interviewing + Schema Therapy (MIST) | CBT; Motivational interview; Schema therapy | Depression | | 8-16 weeks | BDI-II | -- |
| (Hendriks et al., 2020) | RCT | Black or mixed race | ‘Strong Minds’ | Positive Psychology EBP | Depression; Anxiety | | 6 weeks | DASS-21 | Low risk |
| (Himelhoch et al., 2011) | Pre-post | Black or mixed race | ‘Connect’ (telephone CBT) | CBT | Depression | | 14 weeks | HAM-D | -- |
| (Hinton et al., 2004) | Randomised crossover trial | Refugees/asylum seekers; East Asian | CBT | CBT | PTSD; Panic Disorder | | NR | HTQ | Unclear |
| (Hinton et al., 2005) | Randomised crossover trial | Refugees/asylum seekers; East Asian | CBT | CBT | PTSD; Panic Attacks (co morbid) | | NR | CAPS | Unclear |
| (Hinton, Hofmann, Rivera, Otto, & Pollack, 2011) | RCT | Latinx | Culturally adapted CBT (CA-CBT) | CBT | PTSD | | 14 weeks | PTSD-CL | Unclear |
| (Hovey, Hurtado, & Seligman, 2014) | Pre-post | Latinx | CBT | CBT | Depression; stress | | 6 weeks | CES-D | -- |
| (Huey Jr & Pan, 2006) | RCT | East Asian | Culturally adapted One Session Treatment (OST) | One session-treatment (OST) | Phobia | | 1 session (once) | ADIS-IV (phobia subsection) | -- |
| (Hwang et al., 2015) | RCT | East Asian | Culturally adapted CBT (CA-CBT) | CBT | Depression | | 12 weeks | HAM-D | Low risk |
| (Interian, Allen, Gara, & Escobar, 2008) | Pre-post | Latinx | CBT | CBT | Depression (MDD) | | 12 weeks | BDI (Spanish version) | -- |
| (Jones & Warner, 2011) | RCT | Black or mixed race | Claiming Your Connections (CYC) | Cognitive therapy (strengths-based framework) | Depression; Stress | | 10 weeks | CES-D | Low risk |
| (Kananian, Soltani, Hinton, & Stangier, 2020) | RCT | Refugees/asylum seekers | Culturally adapted CBT Plus | CBT | MH NOS | | 6 weeks (12 sessions) | GHQ-28 | Low risk |
| (Kanter, Santiago-Rivera, Rusch, Busch, & West, 2010) | Open trial (treated as pre-post) | Latinx | Behavioural activation-Latino | Behavioural activation (BA) | Depression | | 12-20 weeks | BDI-II | -- |
| (Kanter et al., 2015) | RCT | Latinx | Behavioural activation-Latino | Behavioural activation (BA) | Depression | | 12 sessions (NOS) | HAM-D (Spanish version) | Low risk |
| (Karasz et al., 2015) | RCT | South Asian | The Action to Improve Self-esteem and Health through Asset building (ASHA) | CBT | Depression | | 26 weeks (12 sessions) | PHQ-9 | -- |
| (Kaltman, de Mendoza, Serrano, & Gonzales, 2016) | Pre-post | Latinx | ‘Latinas Saludables’ ('healty latinas') | CBT; Behavioural activation; Motivational interview | Depression; PTSD | | NR | PHQ-9; PTSD-CL | -- |
| (Kayrouz et al., 2015) | Pre-post | Middle Eastern | Arab Wellbeing Course (online) | CBT (iCBT) | Depression; Anxiety | | 8 weeks | PHQ-9; GAD-7 | -- |
| (Knaevelsrud, Brand, Lange, Ruwaard, & Wagner, 2015) | RCT | Middle Eastern | iCBT | CBT (iCBT) | PTSD | | 5 weeks | PDS total score/overall | Low risk |
| (Koch, Ehring, & Liedl, 2020) | RCT | Refugees/asylum seekers | STARC | STARC | MH NOS (transdiagnostic) | | 14 weeks | GHQ-28 | Low risk |
| (Kohn, Oden, Muñoz, Robinson, & Leavitt, 2002) | Quasi-experimental study | Black or mixed race | African American-CBT (AACBT; group) | CBT | Depression (MDD) | | 16 weeks | BDI | -- |
| (Kruse, Joksimovic, Cavka, Woller, & Schmitz, 2009) | RCT | Refugees/asylum seekers | Trauma-Focussed Psychotherapy | CBT; Progressive muscle relaxation; Trauma-psychotherapy | PTSD | | 25 hours (over several weeks) | HTQ (PTSD subscale) | -- |
| (Laperriere et al., 2005) | RCT | BME NOS | Cognitive behavioural  stress management/  expressive supportive therapy (CBSM+) | CBT | Depression | | 10 weeks | BDI | Unclear |
| (Leiler, Wasteson, Holmberg, & Bjärtå, 2020) | Pre-post | Refugees/asylum seekers | ‘AMIN’ (psychoeducational group intervention) | Psychoeducation | MH NOS | | 5 weeks (6 sessions) | RHS | -- |
| (Lindegaard et al., 2019) | RCT | Middle Eastern; Refugees/asylum seekers | iCBT | CBT | Depression | | 8 weeks | BDI-II | Low risk |
| (Lindegaard et al., 2020) | RCT | BME NOS; Refugees/asylum seekers | iCBT (self-help) | CBT | Depression | | 8 weeks | PHQ-9 | Low risk |
| (Lovell et al., 2014) | RCT | BME NOS | Wellbeing intervention | CBT | Depression; Anxiety | | 16 weeks | CORE-OM | -- |
| (Matsumoto et al., 2020) | Pre-post | East Asian | CBT (video conference) | CBT | Anxiety | | 16 weeks | Y-BOCS | -- |
| (Meffert et al., 2014) | RCT | Refugees/asylum seekers; Black or mixed race | IPT | IPT | Depression; PTSD | | 3 weeks | HTQ | Unclear |
| (Miranda, Azocar, Organista, Dwyer, & Areane, 2003) | RCT | BME NOS | CBT + case management | CBT; case management | Depression | | 12 weeks | BDI | -- |
| (Muto, Hayes, & Jeffcoat, 2011) | RCT | East Asian | Acceptance and commitment therapy (ACT) bibliotherapy | Acceptance and commitment therapy (ACT) bibliotherapy | Depressive symptoms; Anxiety | | 8 weeks | GHQ-12 | Low risk |
| (Naeem et al., 2014) | RCT | South Asian | Culturally adapted CBT self-help (CA-CBT-SH) | CBT | Depression | | 12 weeks | HADS (depression subscale) | Low risk |
| (Naeem et al., 2015) | RCT | South Asian | Culturally adapted CBT for psychosis (CA-CBTp) | CBT (CBTp) | Schizophrenia | | 4 months | PANNS (general) | Low risk |
| (Neuner et al., 2008) | Randomised controlled dissemination trial | Refugees/asylum seekers | Trauma counselling | Trauma counselling | PTSD | | 3 weeks | PDS | Low risk |
| (Pan, Huey, & Hernandez, 2011) | RCT | Black or mixed race | Culturally adapted One Session Treatment (OST) | One session-treatment (OST) | Phobia | | 1 session (once) | FSS-III | -- |
| (Piedra & Byoun, 2011) | Pre-post | Latinx | ‘Vida Alegre’ (group manualized CBT) | CBT | Depression | | 10 weeks | CES-D (Spanish version) | -- |
| (Rathod et al., 2013) | RCT | BME NOS | Culturally adapted CBT for psychosis (CA-CBTp) | CBT (CBTp) | Schizophrenia | | 16-20 weeks | CPRS (total) | Low risk |
| (Razali, Hasanah, Aminah, & Subramaniam, 1998) | RCT | East Asian; Religious minority | Religious cultural psychotherapy | Cognitive therapy | Anxiety; Depression | | 6 months | HARS; HAM-D | Unclear |
| (Razali, Aminah, & Khan, 2002) | RCT | East Asian; Religious minority | Religious cultural psychotherapy | Cognitive therapy | Anxiety (generalised anxiety disorder) | | 6 months | HARS | Unclear |
| (Rosmarin, Pargament, Pirutinsky, & Mahoney, 2010) | RCT | Religious minority | Internet based Spiritually Integrated Treatment (SIT) | Psychotherapy | Anxiety | | NR | PSWQ | Low risk |
| (Ryan, Maurer, Lengua, Duran, & Ornelas, 2018) | Pre-post | Latinx | ‘Amigas Latinas Motivando el Alma’ (ALMA) | Mindfulness | Depression; Anxiety | | 5 weeks | PHQ-9 (Spanish version); GAD-7 (Spanish version) | -- |
| (Sander, Laugesen, Skammeritz, Mortensen, & Carlsson, 2019) | Retrospective cohort study | Refugees/asylum seekers | CBT (interpreter) | CBT | PTSD | | 16 sessions | HTQ | -- |
| (Scogin et al., 2007) | RCT | BME NOS | Home-based CBT | CBT | Depressive symptoms; QoL | | 5.3 months (average) | SCL-R-90 (GSI) | Low risk |
| (Shaw, Ward, Pillai, & Hinton, 2018) | RCT | Refugees/asylum seekers; Middle Eastern | Culturally adapted CBT (CA-CBT) | CBT (CBT) | Depression & Anxiety; PTSD | 8 weeks | | HSCL-25 (depression subscale) | Unclear |
| (Shin & Lukens, 2002) | RCT | East Asian | Psychoeducation + Individual Supportive Therapy | Psychoeducation | Schizophrenia | 10 weeks | | BPRS total | Low risk |
| (So et al., 2015) | RCT | East Asian | Meta cognitive training for delusions (MCTd) | Meta cognitive training for delusions (MCTd) | Schizophrenia | 4 weeks | | PSYRATS (delusions) | Low risk |
| (Sonderegger, Rombouts, Ocen, & McKeever, 2011) | Randomised trial (convenience sample) | Refugees/asylum seekers | ‘EMPOWER’ | CBT | Depressive symptoms; Anxiety symptoms | NR | | APAI (two tam scale) | -- |
| (Tol et al., 2020) | Cluster RCT | Refugees/asylum seekers | Self-help PLUS | ACT | Mental health NOS | 5 weeks | | Kessler-6 | Low risk |
| (Ward & Brown, 2015) | Pre-post | Black or mixed race | ‘Oh Happy Day Class’ (OHDC) | CBT (coping with depression) | Depression (MDD) | 12 weeks | | CES-D | -- |

*ACT = acceptance and commitment therapy; ADIS = Anxiety disorder interview schedule; APAI = The Acholi Psychosocial Assessment Instrument; BADS = Behavioural Activation for Depression Scale; BAI = Beck Anxiety Inventory; BDI = Beck Depression Inventory; BPRS = Brief psychiatric rating scale; BSI = brief symptom inventory; CAPS = Clinician-administered PTSD scale; CBT = cognitive behavioural therapy; CDS = Cardiac Depression Scale; CES-D = Centre for Epidemiological Studies Depression Scale; CORE-OM = CORE outcome measure; CPRS = Comprehensive Psychopathological Rating Scale.; CSR = Clinician severity rating; EBT = evidence based treatment; EDE = eating disorder examination; EPDS = Edinburgh Postnatal Depression Scale; FSS = Fear Survey Schedule; GAD = Generalised Anxiety Disorder Assessment; GHQ = General Health Questionnaire; HAM-D = Hamilton Depression Rating Scale; HARS = Hamilton Anxiety Rating Scale; HSCL = Hopkins Symptom Checklist; HTQ = Harvard Trauma Questionnaire; IED-R = Impact of Events Scale Revised; IPT = interpersonal therapy/treatment; MDD = major depressive disorder; NOS = not otherwise specified; NR = not reported/not found; PANNS = Positive and Negative Syndrome Scale of Schizophrenia; PCL-5 =PTSD checklist; PDS = post-traumatic stress diagnostic scale; PDSS = Panic Disorder Severity Scale; PHQ = Patient health questionnaire; PSWQ = Penn State Worry Questionnaire; PSYRATS = Psychotic Symptom Rating Scales; PTA scale = perceived threat of dementia anxiety scale; PTSD = post-traumatic stress disorder; PTSD-CL = PTSD checklist; PCL-C = PTSD checklist (civilian); PTSS = post-traumatic stress symptoms; QIIDS-SR = Quick inventory of depressive symptomology; RCT = randomised controlled trial; RHS; Refugee Health Screener; RoB = risk of bias; SCL = symptom checklist; STARC = Skills training of affect regulation; TMHS = total mental health scale; Y-BOCS = Yale-Brown Obsessive-Compulsive Scale.*

# Appendix D: Study characteristics - systematic reviews

Twenty-one systematic reviews were identified (**Table D.1**). All but one of the reviews focussed primarily on culturally adapted care. Targeted populations varied with some reviews focussing on studies of a specific BME group and others incorporating studies looking at a range of minority populations. Similarly, many of the reviews looked generally at mental health, including a range of conditions while others focussed on a specific diagnosis (most commonly, depression). Seven of the reviews included meta-analyses, for which observed effect sizes ranged from small to very large. Generally, reviews supported the notion that adapted interventions are beneficial compared to controls, in producing better outcomes for BME groups.

**Table D.1: Study characteristics of systematic reviews**

| **Study ID** | **Population** | **Target condition(s)** | **Included studies (n)** | **Participants (n)** | **Study types included** | **Intervention(s)** | **Adaptation type(s) reported** | **Summary of findings** |
| --- | --- | --- | --- | --- | --- | --- | --- | --- |
| (Antoniades, Mazza, & Brijnath, 2014) | Immigrants: first generation | Depression | 15 | NR | 9 quantitative; 5 mixed methods; 1 case study.  Qual, quant & mixed-methods: Descriptive uncontrolled design;  RCT x1;NR experimental study ; Case study ; Pilot RCT ; Randomised pilot study; Pre/post/follow-up study; Pre/post repeated measures study; Pilot case study; | CBT; BA; Collaborative Care Models; Exercise interventions; PST | Cultural adaptation | 15 included studies, of which 9 looked at adapted treatments. Majority of studies of Latino immigrants in the USA. 12 studies looked at use of psychotherapies; the remaining 3 looked at collaborative care models and exercise interventions for depression. CBT and BA improved symptoms when culturally adapted, PST improved symptoms with and without adaptations. CC models and exercise did not improve depression significantly. |
| (Benish, Quintana, & Wampold, 2011) | Racial ethnic minority groups including African American, Asian American and Latino/Hispanic | Unspecified: psychological symptoms/distress | 19 (reporting 21 direct comparisons) | 472 | Published and non-published studies | Psychotherapy various: individual therapy; group therapy; family therapy | Cultural adaptation | Findings favoured adapted therapy over non-adapted therapy significantly.  Cohen’s d = 0.32 (95% CI 0.21-0.43), *I*^2^ = 63.78% |
| (Benuto & O’Donohue, 2015) | Latino/Hispanic | Various mental health conditions | 12 | NR | RCTs with WL control; RCTs without WL control | Culturally sensitive CBT | Cultural adaptation | The authors found evidence that Hispanics may be effectively treated using conventional CBT. They found little evidence that cultural adaptations result in improved effect sizes; cultural adaptations do not show expected homogeneity re cultural tailoring, indication poor understanding of Hispanic culture. |
| (Bhui et al., 2015) | BME | Various mental health conditions | 21 | NR | RCTs (x12); Observational quantitative studies (x2); case series (x3); qualitative study (x1); descriptive case studies (x3) | CBT; family therapy; tele-psychiatry | Cultural adaptation; Provider of treatment (ethnic matching); Method of access | Culturally adapted psychotherapies (CBT and family therapies) showed evidence of benefit, as did tele-psychiatry that included ethnic matching. |
| (Cabassa & Hansen, 2007) | Latino | Depression | 9 (reporting 4 trials) | NR | RCTs | CBT; Collaborative care | Cultural adaptation; Language translations; Medium used to provide treatment | Effective depression care programs in primary care for Latinos populations need to be carefully adapted and modified to fit the social, cultural and economic realities of this population. |
| (Chowdhary et al., 2014) | ‘ethnic minorities in western countries’ | Depression | 20 | NR | RCTs; non-randomised trials | Psychotherapy various: CBT; IPT); psychoeducation; PST; dynamically-oriented therapy | Cultural adaptation | A meta-analysis of 16 studies included in the review showed a significant benefit of adapted interventions. Cultural adaptations of psychotherapies are effective in the treatment of depressive disorders in people from ethnic minorities.  SMD = -0.72 (95% CI -0.94 - -0.49), *I*^2^ = 90% |
| (Collado A., Lim A.C., & MacPherson L., 2016) | Latino | Depression | 36 | NR | RCTs; OLTs | Psychotherapy various: CBT; PST; IPT; BA | Cultural adaptation | The authors concluded that there might be a correlation between cultural adaptations and treatment outcomes |
| (Degnan et al., 2018) | BME | Schizophrenia | 46 journal articles reporting 43 individual studies | 7828 | RCTs | Psycho-social interventions: Family interventions; CBT; Combined interventions; Social skills training; illness management; mindfulness- based psychoeducation | Cultural adaptation | A meta-analysis found a statistically significant post-treatment effect in favour of adapted interventions for total symptom severity, positive symptoms, negative symptoms and general symptoms. The authors conclude that culturally adapted interventions are more effective when compared to treatments as usual |
| (Escobar & Gorey, 2018) | Latino (Hispanic) | Depression | 9 | NR | RCTs; quasi-experimental study | Cognitive behavioural interventions | Cultural adaptation | A meta-analysis indicated an effect in favour of cognitive behavioural interventions that incorporated ‘deep structure’ cultural adaptations. Interventions that incorporated deep structure interventions were more effective than those that included ‘surface structure’ or non-adapted interventions for Hispanic people with depression. Deep structure culturally adapted interventions had a success rate of 15-30% better than those typically observed with other usual treatment practices.  Cohen’s d = 0.41 (95% CI 0.30 – 0.52) |
| (Gearing et al., 2013) | Middle Eastern and Arab | Various mental health conditions | 22 |  | Various: qualitative and quantitive studies | Psychological and mental health treatments | Language translation; Cultural adaptation | Barriers to intervention acceptability within the cultural context were community and system difficulties and problems with clinical engagement processes. Facilitation strategies were working in partnership with the local community and cultural context, engagement with acceptable and traditional intervention characteristics and development of culturally appropriate treatment strategies and techniques. The authors provide recommendations for effective treatment adaptation and translation for Arab communities. |
| (Griner & Smith, 2006) | BME | Various mental health conditions | 76 | 25225 | Experimental and quasi-experimental studies | Various | Cultural adaptation | A meta-analysis indicated a moderately strong effect size in favour of culturally adapted interventions. Interventions targeted towards a specific cultural group were 4 times more effective than those provided to groups of patients from a variety of cultures.  Cohen’s d = 0.45 (95% CI 0.36 – 0.53), Q = 459 |
| (Hall, Ibaraki, Huang, Marti, & Stice, 2016) | BME | Various mental health conditions | 78 | 13998 | Various study designs | Psychological interventions | Cultural adaptation | The meta-analysis showed an  overall effect size that favoured the effectiveness of culturally adapted interventions over other interventions or conditions (no intervention/other intervention). A medium effect size was found to favour culturally adapted interventions over non-adapted versions of the same intervention. Overall, culturally adapted interventions produced better outcomes than comparison conditions.  Cohen’s d = 0.67 (95% CI NR), *I*^2^ = 72% |
| (Hankerson & Weissman, 2012) | African Americans | Various health and mental health conditions | 8 | 910 | RCTs; Open trials; Observational studies | Church-based health promotion programmes | Method of access; Medium used to provide treatment | Only one of the studies included looked at anxiety and depressive symptoms as outcomes. The authors concluded that research on church-based health promotion programs for depression is currently underdeveloped and that the literature on church-based health promotion programs for mental disorders  among African Americans is extremely  limited. Therefore, any conclusions  about the role of the Black church in  mental health care should be interpreted with caution |
| (Healey et al., 2017) | BME | Various health and mental health conditions | 31 | NR | Various study designs | Various | Cultural adaptation | Seventeen of the included studies report at least one significant effect in favour of culturally adapted care. However, there were also findings that favoured the control group or showed no difference. Researchers did not find consistent evidence supporting  implementation of any specific type of adaptation nor increased efficacy with any particular cultural group |
| (Huey & Tilley, 2018) | Asian Americans | Various mental health conditions | 21 (18 included in meta-analysis) | 6377 | Various study designs | Various | Cultural adaptations (cultural tailoring) | Specificity of cultural tailoring was significantly associated with outcomes, with treatments tailored specifically for Asian subgroups (e.g., Chinese Americans) showing the largest effect sizes (Cohen’s d = 1.10), and those with no cultural tailoring or non-Asian tailoring showing the smallest effects (Cohen’s d = 0.25) |
| (Interian A., Lewis-Fernandez R., & Dixon L.B., 2013) | “racial ethnic groups” | Depression; Schizophrenia | 10 (only 3 studies incorporated an adaptation) | NR | RCTs | Collaborative care for depression; Family therapy for schizophrenia | Cultural adaptations (Socio-cultural enhancements) | The authors concluded that collaborative care for depression can help to improve engagement in people from racial-ethnic populations with depression in primary care. The effectiveness of interventions on clinical outcomes was inconsistent. |
| (Kalibatseva & Leong, 2014) | BME | Depression | 16 | NR | Various study designs | Various depression interventions | Cultural adaptations (culturally-sensitive) | No analysis of effectiveness or meta-analysis was undertaken. The authors conclude that majority of culturally sensitive treatments for depression involve general and practical adaptations, such as translating materials or incorporating specific cultural values. |
| (Leske et al., 2016) | Indigenous populations | Depression; Anxiety; PTSD; Personality Disorder; Bipolar disorder; Psychotic Disorder; mood Disorder | 16 (7 studies incorporated adaptations but only 5 of these considered mental health interventions) | NR | RCTs; pre-post studies | Various: psychological/psychosocial interventions; pharmacological interventions; educational interventions | Cultural adaptations | Of the seven culturally adapted psychological/psychosocial intervention studies, all reported significant improvement on at least one measure of symptoms of mental illness, functioning, and alcohol use |
| (Palic & Elklit, 2011) | Refugees | PTSD | 25 (only 4 used adaptations) | NR | Various study designs | CBT | Cultural adaptations (culturally sensitive) | The majority of the studies were treatment studies of different forms of CBT. Only 4 of the studies incorporated culturally sensitive adaptations. Large effect sizes were obtained in some of the CBT studies, indicating that CBT may be suitable for treatment of PTSD with refugees in many cases |
| (Pineros-Leano, Liechty, & Piedra, 2017) | Immigrants - Latinos | Depression | 11 | NR | RCTs; Quasi-experimental studies; pre-post studies | CBT | Cultural adaptations (categorised as (a) cognitive-informational adaptations, (b) affective-motivational adaptations, and (c) environmental adaptations) | The most commonly used cultural adaptations were language, inclusion of migration experience in the therapy and adjustment of literacy level. Culturally adapted CBT to address depressive symptoms showed promising results |
| (Van Loon A., Van Schaik A., Dekker J., & Beekman A., 2013) | “ethnic minorities” | Depression; Anxiety | 9 | NR | RCTs; pre-post studies | CBT; Panic control therapy; Exposure therapy | Cultural adaptations | Culturally adapted treatment for depression and anxiety driven by guidelines, was effective for minority patients from different cultural backgrounds. The authors found some evidence for the effectiveness of the population-specific cultural adaptations.  SMD = 1.06 (95% CI 0.51 – 1.62) |

*BA = behavioural activation; BME = Black and minority ethnic groups; CBT = cognitive behavioural therapy; CI = confidence interval; IPT = interpersonal therapy/treatment; NR = not reported; PTSD = post-traumatic stress disorder; RCTs = randomised controlled trials; SMD = standardised mean difference; WL = waitlist*

# Appendix E: Adaptations applied to all studies

***Table E.1: Adaptations applied to all studies (K=88)***

| **Study ID**  **(first author +publication date)** | **Target population** | **Target condition(s)** | **Adaptation area** | **Specific adaptation types** | **Common factors:  Acceptability & suitability** | **Common factors: Therapeutic relationship** | **Common factors details** |
| --- | --- | --- | --- | --- | --- | --- | --- |
| **Acarturk 2015** | Refugees/Asylum seekers | PTSD; Depression | *Therapist-related* | Language translation |  |  |  |
|  |  |  | *Content-related* | Cultural | Cultural adaptations; Education |  | Culturally sensitive; Psychoeducation for opinion leaders |
|  |  |  | *Organisation-specific* | Time or length of treatment |  |  |  |
| **Acarturk 2016** | Refugees/Asylum seekers | PTSD; Depression | *Therapist-related* | Language translation |  |  |  |
|  |  |  | *Content-related* | Cultural | Cultural adaptations; Education |  | Culturally sensitive; Psychoeducation for opinion leaders |
|  |  |  | *Organisation-specific* | Time or length of treatment |  |  |  |
| **Alavi 2020** | Middle Eastern | Anxiety | *Therapist-related* | Language translation |  | Alliance; Patient feedback | Participants had opportunity to feedback to facilitator throughout; A designated therapist providing feedback support and simplified communication throughout to reduce barriers traditionally experienced by target population |
|  |  |  | *Content-related-related* | Cultural; Language translation; | Cultural adaptations |  | Cultural background of the target population was considered |
|  |  |  | *Organisation-specific* | Form used to provide treatment (online/email) |  |  |  |
| **An 2020** | East Asian | Anxiety | *Therapist-related* |  |  | Alliance; Agreement of treatment goals; Expectations of treatment | Clarify role of therapist to group and set group dynamics; therapist as facilitator and educator; psychosocial education incorporated |
|  |  |  | *Content-related-related-related* | Cultural |  |  | Culturally adapted in terms of reducing ambiguity for Asian populations; Incorporating Tai Chi as behavioural activation component |
|  |  |  | *Organisation-specific* | Form used to provide treatment | Cultural adaptations; Preparation of the patient; Education |  |  |
| **Afuwape 2010** | Black or mixed race | Anxiety; Depression | *Therapist-related* | Provider of treatment |  |  |  |
|  |  |  | *Content-related* |  | Education |  | Health education as part of the intervention package |
|  |  |  | *Organisation-specific* | Method of access |  |  |  |
| **Alegria 2014** | Latino | Depression | *Therapist-related* | Training for provider; Provider of treatment (ethnic matching) |  |  |  |
|  |  |  | *Content-related* | Cultural | Cultural adaptations; Treatment structure |  | Culturally relevant modifications, terminology, metaphors etc; Structure of treatment was flexible |
|  |  |  | *Organisation-specific* | Form used to provide treatment; Time or length of treatment |  |  |  |
| **Alegria 2019** | Latino | Depression; PTSD; General mental health (secondary); Substance use (primary) | *Therapist-related* | Training for provider; Language translation |  |  |  |
|  |  |  | *Content-related* | Cultural | Cultural adaptations |  | Cultural adaptations (limited info 'tailored specifically for Latinos’) |
|  |  |  | *Organisation-specific* | Location of treatment; Form used to provide treatment |  |  |  |
| **Ashing 2014** | Latino | Depression | *Therapist-related* | Training for provider; Provider of treatment; Language translation |  | Alliance | Therapist-related by paraprofessionals with sociocultural similarities to participants |
|  |  |  | *Content-related* | Cultural | Cultural adaptations; Treatment structure |  | Culturally sensitive, culturally competent facilitators, culturally sensitive resources; Order of the treatment domains was flexible |
|  |  |  | *Organisation-specific* |  |  |  |  |
| **Bedoya 2014** | Latino | Depression | *Therapist-related* | Training for provider; Language translation |  | Alliance | Adopted the Engagement Interview Protocol and the DSM5 OCF at first visit |
|  |  |  | *Content-related* | Cultural: Language translation | Cultural adaptations; Education |  | Culturally focussed; Facilitation of patient's knowledge of and resources for getting treatment |
|  |  |  | *Organisation-specific* |  |  |  |  |
| **Beeber 2010** | Latino | Depression | *Therapist-related* | Training for provider; Language translation |  | Alliance | Specific considerations to the development of the TR which underpinned the intervention based on previous work with lived experience ppts with similar demographics to the study group |
|  |  |  | *Content-related* | Cultural | Cultural adaptations |  |  |
|  |  |  | *Organisation-specific* | Location of treatment |  |  |  |
| **Bernardi 2019** | Refugees/asylum seekers | PTSD | *Therapist-related* | Language translation |  | Alliance; Expectations of treatment | Interpreter was viewed also as a facilitator of the therapist-patient relationship |
|  |  |  | *Content-related* | Cultural; Language translation; Religious/faith-based | Cultural adaptations; Preparation of the patient; Treatment structure |  | Treatment provided with a facilitator to support relationship-building; foundation and rationale for treatment discussed; Simplified structural elements |
|  |  |  | *Organisation-specific* |  |  |  |  |
| **Bernstein 2016** | East Asian | Depression | *Therapist-related* |  |  |  |  |
|  |  |  | *Content-related* | Cultural | Cultural adaptations |  | ‘Culturally tailored’ |
|  |  |  | *Organisation-specific* | Time or length of treatment |  |  |  |
| **Bolton 2003** | Black or mixed race | Depression | *Therapist-related* | Training for provider/facilitator; Provider of treatment |  | Agreement of treatment goals; Expectations of treatment | Specific focus on agreeing goals; Initial meeting sets out what group treatment will entail and how it will work |
|  |  |  | *Content-related* | Cultural | Cultural adaptations; Treatment structure |  | Culturally-relevant terminology and modifications to manual based on consultation with layperson group leaders; Flexible treatment structure |
|  |  |  | *Organisation-specific* | Form used to provide treatment (group); Location of treatment |  |  |  |
| **Bonilla-Escobar 2018** | Black or mixed race | Depression; PTSD; Anxiety | *Therapist-related* | Training for provider; Provider of treatment (layperson); Language translation |  | Alliance | Trained laypersons were all survivors of displace and violence themselves and were recognised leaders or caregivers in the community |
|  |  |  | *Content-related* |  | Treatment structure; Education |  | Modular elements can stand alone and be delivered in any order; Psychoeducation provided early in treatment |
|  |  |  | *Organisation-specific* | Location of treatment (community) |  |  |  |
| **Bradley 2006** | East Asian | Schizophrenia | *Therapist-related* | Language translation |  |  |  |
|  |  |  | *Content-related* | Cultural | Cultural adaptations; Education |  | Incorporation of traditional cultural healing practices alongside intervention, informal outreach conducted; Psychoeducation for families |
|  |  |  | *Organisation-specific* | Location of treatment |  |  |  |
| **Cachelin 2018** | Latino | Eating disorder (binge eating) | *Therapist-related* |  |  |  |  |
|  |  |  | *Content-related* | Cultural; Language translation | Cultural adaptations; Education |  | Several cultural adaptations made including socio-cultural sensitivity; Patients were educated on potential benefits of intervention |
|  |  |  | *Organisation-specific* | Time or length of treatment |  |  |  |
| **Cajanding 2016** | East Asian | Depressive symptoms | *Therapist-related* |  |  |  |  |
|  |  |  | *Content-related* | Cultural | Cultural adaptations |  | Culturally adapted to fit the Filipino context |
|  |  |  | *Organisation-specific* |  |  |  |  |
| **Camacho 2015** | Latinx | Depressive symptoms | *Therapist-related* | Training for provider; Language translation |  | Expectations of treatment | Trained ‘depression care specialists’ in the intervention and provided a warm hand-off to confer trust and improve compliance between therapist and patient |
|  |  |  | *Content-related* | Cultural | Cultural adaptations; Treatment structure |  | Altered two of the first key steps of intervention to meet patient need; Modifications to cultural perspectives and beliefs |
|  |  |  | *Organisation-specific* |  |  |  |  |
| **Carter 2003** | Black or mixed race | Panic disorder | *Therapist-related* | Provider of treatment |  | Alliance | Developed through therapist displaying ethnic sensitivity |
|  |  |  | *Content-related* | Cultural | Cultural adaptions |  | Cultural sensitivity/awareness |
|  |  |  | *Organisation-specific* | Form used to provide treatment |  |  |  |
| **Chien 2012** | East Asian | First-episode mental illness (NOS) | *Therapist-related* |  |  |  |  |
|  |  |  | *Content-related* | Cultural | Cultural adaptations; Education; Preparation of the patient | | Adopted strategies to address traditional Chinese cultural tenets; Educational needs identified and incorporated; orientation and understanding of MH focussed on interdependence and collective actions |
|  |  |  | *Organisation-specific* |  |  |  |  |
| **Choi 2012** | East Asian | Depression | *Therapist-related* | Language translation |  |  |  |
|  |  |  | *Content-related* | Cultural; Language translation | Cultural adaptations |  | Culturally attuned re terms of reference and images |
|  |  |  | *Organisation-specific* |  |  |  |  |
| **Choy 2016** | East Asian | Depressive symptoms | *Therapist-related* |  |  | Alliance; Empathy | Pre-intervention interview to establish rapport; Observer assisted therapist to nurture individual behavioural and emotional concerns |
|  |  |  | *Content-related* | Cultural | Cultural adaptations; Preparation of the patient |  | Culturally-modified manual, culturally sensitive protocol, culturally-appropriate homework materials |
|  |  |  | *Organisation-specific* | Form used to provide treatment; Location of treatment |  |  |  |
| **Collado 2014** | Latino | Depressive symptoms | *Therapist-related* | Language translation |  |  |  |
|  |  |  | *Content-related* | Language translation |  |  |  |
|  |  |  | *Organisation-specific* |  |  |  |  |
| **Collado 2016** | Latino | Depression: Major Depressive Disorder | *Therapist-related* | Language translation |  |  |  |
|  |  |  | *Content-related* | Language translation |  |  |  |
|  |  |  | *Organisation-specific* |  |  |  |  |
| **Comas-Diaz 1981** | Latino | Depressive symptoms | *Therapist-related* | Language translation; Provider of treatment (ethnic matching) |  | Alliance | Therapist from same cultural background |
|  |  |  | *Content-related* | Cultural | Cultural adaptations; Treatment structure |  | Treatment consistent with cultural values; Group therapy consistent with family/social values |
|  |  |  | *Organisation-specific* | Form used to provide treatment (group) |  |  |  |
| **Cooper 2013** | Black or mixed race | Depression: Major Depressive Disorder | *Therapist-related* | Provider of treatment (ethnic matching) |  | Alliance | Clinicians received an 'intervention' aimed to help them develop rapport |
|  |  |  | *Content-related* | Cultural | Cultural adaptations; Treatment structure; Education |  | Culturally-tailored focussed on access barriers, social context, patient-provider relationship + culturally targeted materials; Flexible structure; Education element to treatment |
|  |  |  | *Organisation-specific* |  |  |  |  |
| **Dahne 2019** | Latino | Depressive symptoms | *Therapist-related* |  |  |  |  |
|  |  |  | *Content-related* | Language translation | Education |  | Psychoeducation on relationship between mood and activities provided |
|  |  |  | *Organisation-specific* | Form used to provide treatment |  |  |  |
| **De Graaff 2020** | Refugees/Asylum seekers | Depression; Anxiety | *Therapist-related* | Provider of treatment (peers); Training for provider; Language translation |  | Alliance | Peer provided to support alliance |
|  |  |  | *Content-related* | Cultural | Cultural adaptation |  | ‘Culturally adapted’ |
|  |  |  | *Organisation-specific* |  |  |  |  |
| **Drozdek 2012** | Refugees/Asylum seekers | PTSD | *Therapist-related* |  |  | Alliance; Expectations of treatment; Agreement of treatment goals | Phase one focusses specifically on building alliance; Phase one includes agreement of treatment goals; Phase one includes setting out expectations |
|  |  |  | *Content-related* | Cultural | Cultural adaptations; Education; Treatment structure |  | Cultural adaptations for displaced people; Psychoeducation formed part of the early and later phases; Included nonverbal elements |
|  |  |  | *Organisation-specific* | Time or length of treatment; Location of treatment; Form used to provide treatment (group) |  |  |  |
| **Dwight-Johnson 2011** | Latino | Depression | *Therapist-related* | Language translation; Provider of treatment (ethnic match) |  | Alliance |  |
|  |  |  | *Content-related* | Cultural; language translation | Cultural adaptations; Treatment structure | |  |
|  |  |  | *Organisation-specific* | Location of treatment; Form used to provide treatment (phone); Method of access; Time of length of treatment |  |  |  |
| **Feldman 2016** | Latino | Panic Disorder | *Therapist-related* | Training for provider; Language translation |  |  |  |
|  |  |  | *Content-related* | Cultural | Cultural adaptations |  | Modified treatment to address cultural characteristics of Latino pop |
|  |  |  | *Organisation-specific* |  |  |  |  |
| **Gallagher-Thompson 2007** | East Asian | Depression | *Therapist-related* | Language translation |  | Patient feedback | Final session reviewed what worked and did not work |
|  |  |  | *Content-related* | Cultural; Language translation | Cultural adaptations; Treatment structure |  | Culturally acceptable terms used; Modules could be presented in any order |
|  |  |  | *Organisation-specific* | Location of treatment; Form used to provide treatment |  |  |  |
| **Gallagher-Thompson 2008** | Latino | Depressive symptoms | *Therapist-related* | Language translation |  |  |  |
|  |  |  | *Content-related* | Cultural; Language translation | Cultural adaptations; Education |  | Culturally sensitive to concerns of Latino caregivers; Participants receive education in session 1 on negative effects of stress on body and mind and how treatment might help; |
|  |  |  | *Organisation-specific* | Form used to provide treatment (small groups) |  |  |  |
| **Gallagher-Thompson 2010** | East Asian | Depression | *Therapist-related* |  |  |  |  |
|  |  |  | *Content-related* | Cultural; Language translation | Cultural adaptations |  | ‘Culturally-tailored’ |
|  |  |  | *Organisation-specific* | Form used to provide treatment |  |  |  |
| **Glueckauf 2012** | Black or mixed race | Depression | *Therapist-related* |  |  |  |  |
|  |  |  | *Content-related* |  |  |  |  |
|  |  |  | *Organisation-specific* | Form used to provide treatment |  |  |  |
| **Gonyea 2016** | Latino | Depression; Anxiety | *Therapist-related* | Language translation |  | Alliance |  |
|  |  |  | *Content-related* | Cultural | Cultural adaptations |  |  |
|  |  |  | *Organisation-specific* | Location of treatment |  |  |  |
| **Grote 2009** | BME NOS | Perinatal depression | *Therapist-related* |  |  | Agreement of treatment goals; Alliance | Collaborative problem solving of identified barriers to care; Trust-building |
|  |  |  | *Content-related* | Cultural | Cultural adaptations; Treatment structure |  | Culturally relevant modifications, terminology, metaphors etc. |
|  |  |  | *Organisation-specific* | Time or length of treatment; Location of treatment |  |  |  |
| **Habib 2015** | South Asian | Schizophrenia | *Therapist-related* |  |  |  |  |
|  |  |  | *Content-related* | Cultural | Cultural adaptations |  |  |
|  |  |  | *Organisation-specific* |  |  |  |  |
| **Hahm 2019** | East Asian | Depression | *Therapist-related* | Provider of treatment (ethnic matching); Language translation |  | Alliance; | Initial sessions emphasise building therapeutic relationship; Cultivating a space free of judgement and fostering a sense of safety |
|  |  |  | *Content-related* | Cultural; Language translation | Cultural adaptations; Preparation of the patient; Education |  | Incorporates culture-specific elements in the therapeutic process; Initial sessions incorporate educational aspects |
|  |  |  | *Organisation-specific* |  |  |  |  |
| **Hahm 2020** | East Asian; South Asian | Anxiety; Depression; PTSD | *Therapist-related* | Provider of treatment (ethnic matching); Language translation |  | Alliance; | Initial sessions emphasise building therapeutic relationship; Cultivating a space free of judgement and fostering a sense of safety |
|  |  |  | *Content-related* | Culture; Language translation | Cultural adaptations; Preparation of the patient; Education |  | Incorporates culture-specific elements in the therapeutic process; Initial sessions incorporate educational aspects |
|  |  |  | *Organisation-specific* |  |  |  |  |
| **Heilemann 2011** | Latino | Depression | *Therapist-related* |  |  | Alliance | Through use of collaborative maps co-created by patient and therapist in early stages |
|  |  |  | *Content-related* | Cultural | Cultural adaptations; Education; Preparation of the patient; | | Sensitive to cultural milieu; Education on CBT and analysis of automatic thoughts; |
|  |  |  | *Organisation-specific* | Form used to provide treatment; Location of treatment (community setting); Time or length of treatment |  |  |  |
| **Hendriks 2020** | Black or mixed race | Depression; Anxiety | *Therapist-related* | Training for provider/facilitator |  |  |  |
|  |  |  | *Content-related* | Cultural; Religious/faith-based | Cultural adaptations; Treatment structure; Education |  | Followed a comprehensive cultural adaptation framework and drew upon existing methods of adaptations (surface and deep structure); Some modules were dropped and structural changes were made to better suit target population; Psychoeducation elements |
|  |  |  | *Organisation-specific* | Time or length of treatment |  |  |  |
| **Himelhoch 2011** | Black or mixed race | Depression | *Therapist-related* | Training for provider |  | Alliance | Explicit methods taken to establish alliance with particular population group in early stages |
|  |  |  | *Content-related* | Cultural | Cultural adaptations |  | Cultural appropriateness determined by lived experience advisers |
|  |  |  | *Organisation-specific* | Form used to provide treatment (phone); Time or length of treatment (flexible) |  |  |  |
| **Hinton 2004** | Refugees/asylum seekers; East Asian | PTSD; Panic Disorder | *Therapist-related* |  |  |  |  |
|  |  |  | *Content-related* | Cultural | Cultural adaptations |  | Culturally appropriate visualisation representing cultural values and cultural modifications |
|  |  |  | *Organisation-specific* |  |  |  |  |
| **Hinton 2005** | Refugees/asylum seekers; East Asian | PTSD; Panic Attacks | *Therapist-related* |  |  |  |  |
|  |  |  | *Content-related* | Cultural | Cultural adaptations |  | Culturally appropriate visualisation representing cultural values and cultural modifications |
|  |  |  | *Organisation-specific* |  |  |  |  |
| **Hinton 2011** | Latino | PTSD | *Therapist-related* | Language translation |  |  |  |
|  |  |  | *Content-related* | Cultural | Cultural adaptations; Education |  | Culturally appropriate and specific analogies and imagery, culturally appropriate visualization representing cultural values; Education about PTSD using imagery |
|  |  |  | *Organisation-specific* |  |  |  |  |
| **Hovey 2014** | Latino | Depression | *Therapist-related* | Language translation; Provider of treatment (layperson in additional clinician); Training for provider (layperson assisting) |  | Alliance; Agreement of treatment goals | Early sessions focussed on patient contributions and collaborative working, establishing trust; Shared goal-setting |
|  |  |  | *Content-related* | Cultural | Cultural adaptations; Education |  | Culturally responsive support groups, cultural values incorporated; Educational materials |
|  |  |  | *Organisation-specific* | Location of treatment; Form used to provide treatment; Time or length of treatment |  |  |  |
| **Huey Jr 2006** | East Asian | Phobia | *Therapist-related* | Training for provider |  |  |  |
|  |  |  | *Content-related* | Cultural | Cultural adaptations |  | Culture-responsive, adaptations derived from research, scholars and recommendations |
|  |  |  | *Organisation-specific* |  |  |  |  |
| **Hwang 2015** | East Asian | Depression | *Therapist-related* | Language translation |  | Alliance; Collecting patient feedback | Improving the client-therapist relationship is part of framework; reviewing and refining adaptions and testing the adapted interventions with stakeholders |
|  |  |  | *Content-related* | Cultural | Cultural adaptations; Preparation of the patient |  | Incorporates cultural beliefs about mental illness |
|  |  |  | *Organisation-specific* |  |  |  |  |
| **Interian 2008** | Latino | Depression (MDD) | *Therapist-related* | Language translation |  | Expectations of treatment; Alliance | Potential benefits of treatment communicated using Spanish phrases and terms; Use of terms/sayings to complement therapeutic techniques to ensure constant discussion between therapist and patient |
|  |  |  | *Content-related* | Cultural | Cultural adaptations |  | Culturally relevant terminology and culturally sensitive language and approaches |
|  |  |  | *Organisation-specific* | Location of treatment |  |  |  |
| **Jones 2011** | Black or mixed race | Depression | *Therapist-related* | Provider of treatment |  |  |  |
|  |  |  | *Content-related* | Cultural | Cultural adaptations |  |  |
|  |  |  | *Organisation-specific* | Form used to provide treatment (group) |  |  |  |
| **Kananian 2020** | Refuges/asylum seekers | MH NOS | *Therapist-related* | Training for provider; Language translation |  | Alliance | Steps to ensure safe, comfortable environment |
|  |  |  | *Content-related* | Cultural; Language translation | Cultural adaptations; Education |  | Culturally adapted imagery and terms of reference; Each session started with psychoeducation |
|  |  |  | *Organisation-specific* | Form used to provide treatment (group) |  |  |  |
| **Kanter 2010** | Latino | Depression | *Therapist-related* | Language translation; Training for provider/facilitator |  | Agreement of treatment goals | Therapists work collaboratively to agree goals and include the family give importance of family in this group |
|  |  |  | *Content-related* | Cultural; Language translation | Cultural adaptations; Treatment structure; Education |  | Culturally sensitive activation targets, incorporation of Latino specific values and beliefs; Additions to and structuring of final sessions; Education about therapy in session one |
|  |  |  | *Organisation-specific* | Time or length of treatment |  |  |  |
| **Kanter 2015** | Latino | Depression | *Therapist-related* | Language translation; Training for provider/facilitator |  | Agreement of treatment goals | Therapist worked collaboratively with the client to set goals |
|  |  |  | *Content-related* | Cultural; Language translation | Cultural adaptations |  | Increased emphasis on culturally important resources as family and community, terms of reference acronyms that did not translate well |
|  |  |  | *Organisation-specific* | Time or length of treatment; Method of access (access route alternative) |  |  |  |
| **Karasz 2015** | South Asian | Depression | *Therapist-related* |  |  | Agreement of treatment goals | Group goal-getting |
|  |  |  | *Content-related* | Cultural | Cultural adaptations; Education |  | Culturally synchronous approach; Educational component of each session |
|  |  |  | *Organisation-specific* | Form used to provide treatment (group) |  |  |  |
| **Kaltman 2016** | Latino | Depression; PTSD | *Therapist-related* |  |  | Alliance; Agreement of treatment goals | Initial exercise designed to induce trust-building between interventionist and patient; Ongoing goal setting and review |
|  |  |  | *Content-related* | Cultural | Cultural adaptations; Preparation of the patient; Education | | MI techniques used to increase readiness for participation in groups; Psychoeducation component was made relevant to Latinos |
|  |  |  | *Organisation-specific* | Time or length of treatment; Form used to provide treatment (group and individual sessions) |  |  |  |
| **Kayrouz 2015** | Middle Eastern | Depression; Anxiety | *Therapist-related* |  |  |  |  |
|  |  |  | *Content-related* | Cultural; Language translation | Cultural adaptations; Education; Preparation of patient |  | Culturally-modified, culturally relevant terminology; Module educating ppts on depression and anxiety and symptoms using terms relevant to the target pop; Basic principles of CBT included modifications to make it appropriate for target group's context |
|  |  |  | *Organisation-specific* | Form used to provide treatment (internet) |  |  |  |
| **Knaevelsrud 2015** | Middle Eastern | PTSD | *Therapist-related* | Language translation; Training for provider/facilitator |  | Expectations of treatment | Therapists responded to patient expectations of healthcare professional - provided straight instructions and decisiveness; |
|  |  |  | *Content-related* | Cultural; Language translation; Religious/faith based | Cultural adaptations |  | Explicit respect towards the concept of family in line with cultural views |
|  |  |  | *Organisation-specific* |  |  |  |  |
| **Kohn 2002** | Black or mixed race | Depression (MDD) | *Therapist-related* |  |  |  |  |
|  |  |  | *Content-related* | Cultural | Cultural adaptations; Treatment structure |  | Changes to some of the language used to describe CBT techniques, African American anecdotes to illustrate concepts; Added specific modules to address issues relevant to AA women |
|  |  |  | *Organisation-specific* |  |  |  |  |
| **Koch 2020** | Refugees/asylum seekers | MH NOS | *Therapist-related* |  |  | Agreement of treatment goals; Expectations of treatment | Clarifying the role of the therapist (= Person) and the clients’ expectations towards a treatment (= Goals) in an initial clinical interview |
|  |  |  | *Content-related* | Cultural | Cultural adaptations; Education |  | Offered same sex groups due to cultural barriers; Expressing ideas in culturally relevant visual and verbal forms |
|  |  |  | *Organisation-specific* | Form used to provide treatment (group) |  |  |  |
| **Kruse 2009** | Refugees/asylum seekers | PTSD | *Therapist-related* | Language translation |  | Alliance | First stage dedicated to development of treatment alliance where a relationship based on respect, truthful information and emotional bonding was established; Care taken to ensure a safe, healing space |
|  |  |  | *Content-related* | Cultural | Cultural adaptations; Education |  | Adapted principles from the trauma-specific stabilization phase for war refugees + culture-related issues; First stage dedicated to education including psychoeducation |
|  |  |  | *Organisation-specific* |  |  |  |  |
| **Laperriere 2005** | BME NOS | Depression | *Therapist-related* |  |  |  |  |
|  |  |  | *Content-related* | Cultural | Cultural adaptations |  | Culturally-relevant emphasis |
|  |  |  | *Organisation-specific* | Form used to provide treatment |  |  |  |
| **Lindegaard 2019** | Middle Eastern; Refugees/asylum seekers | Depression | *Therapist-related* | Language translation |  |  |  |
|  |  |  | *Content-related* | Cultural; Language translation | Cultural adaptations |  | Modifications made to ensure cultural appropriateness included focus on positive social reinforcers and making the intervention more in line with collectivistic orientation of the target population's culture |
|  |  |  | *Organisation-specific* | Form used to provide treatment (online); Method of access |  |  |  |
| **Leiler 2020** | Refugees/asylum seekers | MH NOS | *Therapist-related* | Training for provider/facilitator; Provider of treatment (layperson/untrained); Language translation |  | Patient feedback | Qualitative feedback collected throughout from both staff and patients; Trained students to provide treatment |
|  |  |  | *Content-related* | Cultural; Language translation | Cultural adaptations; Education; Preparation of patient |  | Culturally inappropriate material removed |
|  |  |  | *Organisation-specific* | Form used to provide treatment (group); Location of treatment (asylum centre) |  |  |  |
| **Lindegaard 2020** | Refugees/asylum seekers | Depression | *Therapist-related* |  |  |  |  |
|  |  |  | *Content-related* | Cultural; Language translation | Cultural adaptations; Treatment structure |  | Culturally adapted in line with cultural stigma about mental health; Treatment format adapted for participant group |
|  |  |  | *Organisation-specific* | Form used to provide treatment (online) |  |  |  |
| **Lovell 2014** | Black or mixed race; South Asian | Depression; Anxiety | *Therapist-related* | Training for provider/facilitator |  | Agreement of treatment goals | Collaborative goal identification |
|  |  |  | *Content-related* | Cultural | Cultural adaptations |  | Culturally-sensitive terminology and phrasing, reference to target group specific challenges |
|  |  |  | *Organisation-specific* | Form used to provide treatment (telephone or face-to-face offered); Method of access (referral options) |  |  |  |
| **Matsumoto 2020** | East Asian | Anxiety (various types) | *Therapist-related* |  |  |  |  |
|  |  |  | *Content-related* |  |  |  |  |
|  |  |  | *Organisation-specific* | Form used to provide treatment (videoconference) |  |  |  |
| **Meffert 2014** | Refugees/asylum seekers; Black or mixed race | Depression; PTSD | *Therapist-related* | Training for provider; Provider of treatment; Language translation |  |  |  |
|  |  |  | *Content-related* | Language translation |  |  |  |
|  |  |  | *Organisation-specific* | Time or length of treatment; Location of treatment |  |  |  |
| **Miranda 2003** | BME NOS | Depression | *Therapist-related* | Training for provider; Language translation |  |  |  |
|  |  |  | *Content-related* | Cultural; Language translation | Cultural adaptations |  | More personalised interactions |
|  |  |  | *Organisation-specific* | Time or length of treatment |  |  |  |
| **Muto 2011** | East Asian | Depressive symptoms; Anxiety | *Therapist-related* |  |  |  |  |
|  |  |  | *Content-related* | Language translation; Cultural | Cultural adaptations |  | Culturally-relevant terms of reference and metaphors |
|  |  |  | *Organisation-specific* |  |  |  |  |
| **Naeem 2014** | South Asian | Depression | *Therapist-related* |  |  |  |  |
|  |  |  | *Content-related* | Cultural; Religious/faith-based | Cultural adaptations; Education |  | Culturally-relevant terms of reference and stories; Psychoeducation provided at start |
|  |  |  | *Organisation-specific* |  |  |  |  |
| **Naeem 2015** | South Asian | Schizophrenia | *Therapist-related* |  |  |  |  |
|  |  |  | *Content-related* | Cultural; Religious/faith-based | Cultural adaptations; Treatment structure |  | Culturally appropriate terms of reference, involvement of family member in care enhances acceptability; Added session or the family, family member of co-therapist, flexibility of structure |
|  |  |  | *Organisation-specific* |  |  |  |  |
| **Neuner 2008** | Refugees/asylum seekers | PTSD | *Therapist-related* | Training for provider; Provider of treatment |  | Empathy | The lay people trained were refugees themselves and were chosen because of their ability to empathize with ppts |
|  |  |  | *Content-related* |  | Education; Preparation of the patient; Treatment structure |  | Psychoeducation about PTSD before treatment begun; Prepared ppts for what treatment would entail; Flexible treatment structure |
|  |  |  | *Organisation-specific* |  |  |  |  |
| **Pan 2011** | Black or mixed race | Phobia | *Therapist-related* |  |  | Alliance | Focussed on vertical relationship which fosters directive therapy to encourage alliance |
|  |  |  | *Content-related* | Cultural | Cultural adaptations; Education |  | Addressing the participant’s cultural background and acculturation status, semantic modifications; Psychoeducation |
|  |  |  | *Organisation-specific* |  |  |  |  |
| **Peidra 2012** | Latino | Depression | *Therapist-related* | Training for provider; Language translation |  |  |  |
|  |  |  | *Content-related* | Cultural; Language translation | Cultural adaptations; Preparation of the patient |  | Modifications to terminology and language and to reflect experience of migration and acculturation; Specific modifications to provide orientation to the intervention and therapeutic process |
|  |  |  | *Organisation-specific* | Time or length of treatment; Method of access; Location of treatment; Medium used to provide treatment (group) |  |  |  |
| **Rathod 2013** | BME NOS | Schizophrenia | *Therapist-related* |  |  |  |  |
|  |  |  | *Content-related* | Cultural; Training for provider/facilitator | Cultural adaptations |  | Culturally-based patient health beliefs, attributions concerning psychosis, attention to help seeking pathways, |
|  |  |  | *Organisation-specific* |  |  |  |  |
| **Razili 1998** | East Asian; Religious minority | Anxiety | *Therapist-related* |  |  | Alliance (rapport) | Established a good rapport by not preventing ppts from seeking additional religious support |
|  |  |  | *Content-related* | Cultural; Religious/faith-based | Cultural adaptations |  | Socio-cultural adaptations in addition to specific religious/faith messages incorporated |
|  |  |  | *Organisation-specific* |  |  |  |  |
| **Razili 2002** | East Asian; Religious minority | Anxiety (Generalised anxiety disorder) | *Therapist-related* |  |  | Alliance (rapport) | Incorporated discussion about cultural beliefs of mental illness to strengthen therapeutic relationship and develop rapport |
|  |  |  | *Content-related* | Cultural; Religious/faith-based | Cultural adaptations |  | Socio-cultural adaptations in addition to specific religious/faith messages incorporated |
|  |  |  | *Organisation-specific* |  |  |  |  |
| **Rosmarin 2010** | Religious minority | Anxiety | *Therapist-related* |  |  |  |  |
|  |  |  | *Content-related* | Cultural; Religious/faith-based | Cultural adaptations; Preparation of the patient | | Content-related is adapted in line with Jewish sources and folk-tales; Introduction prepares and encourages patients to engage in intervention |
|  |  |  | *Organisation-specific* |  |  |  |  |
| **Ryan 2018** | Latino | Depression; Anxiety | *Therapist-related* |  |  |  |  |
|  |  |  | *Content-related* | Cultural; Language translation | Cultural adaptations; Preparation of the patient; Education |  | Adapted to be culturally relevant to Latina immigrant mothers e.g. culturally relevant imagery, vignettes, terms etc.; Orientation pre-intervention; Education about key terms and dialogues |
|  |  |  | *Organisation-specific* | Location of treatment; Time or length of treatment; Method of access (open) |  |  |  |
| **Sander 2019** | Refugees/asylum seekers | PTSD | *Therapist-related* |  |  |  |  |
|  |  |  | *Content-related* | Language translation |  |  |  |
|  |  |  | *Organisation-specific* |  |  |  |  |
| **Scogin 2007** | BME NOS | Depressive symptoms | *Therapist-related* | Provider of treatment; Training for provider/facilitator |  |  |  |
|  |  |  | *Content-related* | Cultural | Cultural adaptations; Treatment structure | | Inclusion of family member or friend as a facilitator; Variable amount of sessions availble |
|  |  |  | *Organisation-specific* | Medium used to provide treatment; Location of treatment |  |  |  |
| **Shaw 2018** | Refugees/Asylum seekers; Middle Eastern | Depression; Anxiety | *Therapist-related* | Provider of treatment; Training for provider/facilitator |  |  |  |
|  |  |  | *Content-related* | Cultural | Cultural adaptations; Education |  | Incorporated culturally and religiously appropriate symbols and imagery; Initial education on trauma-related disorders and emotion |
|  |  |  | *Organisation-specific* | Medium used to provide treatment; Time or length of treatment |  |  |  |
| **Shin 2002** | East Asian | Schizophrenia | *Therapist-related* | Language translation |  | Agreement of treatment goals | Goals specified for each module |
|  |  |  | *Content-related* | Cultural; Language translation | Cultural adaptations; Education |  | Traditional disease concepts incorporated into sessions, inclusion of family members offered; Educational techniques designed to enhance ppts' learning and maintain attention |
|  |  |  | *Organisation-specific* |  |  |  |  |
| **So 2015** | East Asian | Schizophrenia | *Therapist-related* | Language translation |  |  |  |
|  |  |  | *Content-related* | Cultural; Language translation | Cultural adaptations |  | Culturally-relevant terms of reference, replaced Western examples and scenarios with those that are culture-neutral or more familiar to the HK Chinese population |
|  |  |  | *Organisation-specific* |  |  |  |  |
| **Sonderegger 2011** | Refugees/asylum seekers | Mental health NOS (including depressive symptoms) | *Therapist-related* | Training for facilitator/provider |  | Agreement of treatment goals | Goal-setting is covered in the first phase |
|  |  |  | *Content-related* | Cultural; Language translation | Cultural adaptations |  | Culturally sensitive and relevant activities, includes forgiveness/reconciliation component |
|  |  |  | *Organisation-specific* | Medium used to provide treatment (group) |  |  |  |
| **Tol 2020** | Refugees/asylum seekers | Mental health NOS | *Therapist-related* | Training for provider/facilitator; Provider of treatment (layperson); Language translation |  |  |  |
|  |  |  | *Content-related* | Language translation | Education |  | Psychoeducation and engagement elements |
|  |  |  | *Organisation-specific* | Medium used to provide treatment (self-help, group); Method of access (rapid) |  |  |  |
| **Ward 2015** | Black or mixed race | Depression (MDD) | *Therapist-related* | Provider of treatment (ethnic match); Training for provider/facilitator |  | Alliance | Methods taken specifically to elucidate trust and respect with AA clients |
|  |  |  | *Content-related* | Cultural; Religious adaptations | Cultural adaptations; Education |  | Culturally-specific incorporating AA cultural beliefs into treatment; Psychoeducation incorporating AA issues |
|  |  |  | *Organisation-specific* | Medium used to provide treatment (group) |  |  |  |

*AA = African American; CBT = cognitive behavioural therapy; PTSD = post-traumatic stress disorder*

# Appendix F: Supplementary results and analyses

## **Search results and data acquisition**

The initial search (April 2019) yielded 7640 results of which 235 potentially relevant articles were considered at full-text. One hundred and forty-nine articles were excluded leaving 86 for inclusion in the review. Of these, 66 were primary studies. Reference lists of 21 eligible systematic reviews were screened, leading to identification of an additional 8 studies which met inclusion criteria. Details of primary studies can be found in **Appendix C** and systematic reviews in **Appendix D**.

There were 13 primary studies for which additional data was requested from the corresponding author. Of these, 6 authors responded and were able to provide missing data (Alegría et al., 2019, 2014; Cajanding, 2016; Dahne et al., 2019; Habib et al., 2015; Shaw et al., 2018). A further 2 study authors responded but were unable to access or provide missing data (Camacho et al., 2015; Kohn et al., 2002). There was no response from the authors of the remaining 5 studies (Comas-Díaz, 1981; Cooper et al., 2013; Huey Jr & Pan, 2006; Karasz et al., 2015; Miranda et al., 2003). In total, 7 studies were excluded from consideration for analysis on the basis of insufficient data. However, all studies are included in the study characteristics table (**Table C.1**) and were used to answer research question 1 about frequency of adaptations reported.

### Update searches

An update search in July 2020 retrieved 579 additional studies, after removing duplicates. Of these, 13 primary studies were considered at full text. Six of these studies met inclusion criteria; 5 RCTs (including a cluster randomised controlled trial) and 1 pre-post study. Missing data was requested from one study author (Alavi & Hirji, 2020) but was not received and so could not be considered for inclusion in the meta-analysis. A further update search was run in December 2020 leading to the identification of 209 studies after removing duplicates. Of these, 24 primary studies were considered at full text. Eight met inclusion criteria; 5 RCTs and 3 pre-post studies (see **Figure 1** for the study identification flowchart).

In total, 88 primary studies were included, of which 57 were RCTs (including pilots) initially considered for meta-analysis; we included 51studies in the main analyses, following a sensitivity analysis and the removal of 6 studies with unusually large effect sizes (i.e., greater than 2). Study characteristics are available in **Appendix C**.

## **Sub-group analyses (RCTs) by target population group**

**Table F.1: Target population/group categories**

| Target population/group | Included in population group |
| --- | --- |
| Latinx | Latinx; Latino; Latina; Hispanic (Puerto Rican) |
| Black or mixed race | African; African American; African British; Black African; Black Caribbean; "Black"; mixed race Black African or Caribbean |
| East Asian | East Asian; Asian (Chinese, Korean, Japanese, Vietnamese) |
| South Asian | South Asian; Asian (Indian; Pakistani; Bangladeshi; Sri Lankan) |
| NOS/mixed groups/immigrants/migrants | "racial/ethnic diverse" “immigrants” “migrants” or no other info; mixed ethnic/racial groups |
| Middle Eastern | Middle Eastern; Arabian |
| Religious minority | Jewish; Muslim |
| Refugees or asylum seekers | Refugees; asylum seekers; displaced people |

*BME = Black and other minority ethnic; NOS = not otherwise specified*

**Table F.2: Number of RCTs targeting BME groups**

| **Target population/group** | **Number of studies (K)** | **Study IDs of included RCTs** |
| --- | --- | --- |
| East Asian | 14 | An (2020); Bradley (2006); Cajanding (2016); Choi (2012); Choy (2016); Gallagher-Thompson (2007); Gallagher-Thompson (2010); Hwang (2005); Muto (2011); Razali (1998)*; Razali (2002)*; Shin (2002); So (2015); Hahm (2019) |
| Latinx | 12 | Alegria (2019); Ashing (2014); Beeber (2010); Cachelin (2018); Collado (2016); Dahne (2019); Dwight-Johnson (2011); Feldman (2016); Gallgher-Thompson (2008); Gonyea (2016); Hinton (2011); Kanter (2015) |
| Refugees/asylum seekers | 10 | Acarturk (2015); Acarturk (2016); De Graaff (2020); Koch (2020); Meffert (2014)*; Neuner (2009); Shaw (2018)*; Lindegaard (2019)*; Linedegaard (2020)*; Tol (2020) |
| Black or mixed race | 8 | Afuwape (2010); Bolton (2003); Bonilla-Escobar (2018); Carter (2003); Glueckauf (2012); Jones (2011); Meffert (2014)*; Hendriks (2020) |
| NOS/mixed groups | 5 | Laperriere (2005); Lindegaard (2020)*; Grote (2009); Rathod (2013); Scogin (2007) |
| Religious minority | 3 | Razali (1998)*; Razali (2002)*; Rosmarin (2010) |
| Middle Eastern | 3 | Knaevelsrud (2015); Shaw (2018)*; Lindegaard (2019)* |
| South Asian | 2 | Naeem (2014); Naeem (2015) |

**studies which explicitly targeted people from one of the ‘BME’ groups who also belonged to ‘religious minority’ or ‘refugees/asylum seekers’ groups.*

**Table F.3: Meta-analyses of adapted interventions for different target population subgroups**

| **Target population** | **K (Number of comparisons)** | **Hedge’s g (95% CI)** | **p-value** | ***I*^2^** |
| --- | --- | --- | --- | --- |
| East Asian | 14 (15) | -0.43 (-0.71, -0.16) | .002 | 78.55% |
| Latinx | 12 (13) | -0.48 (-0.84, -0.11) | .011 | 86.92% |
| Refugees or asylum seekers | 10 (11) | -0.99 (-1.35, -0.64) | <.001 | 81.98% |
| Black or mixed race | 8 (10) | -0.70 (-0.95, -0.46) | <.001 | 62.59% |
| NOS/mixed groups/’immigrants/migrants’ | 5 (5) | -0.62 (-1.04, -0.19) | .004 | 71.14% |
| Religious minority | 3 (4) | -0.18 (-0.52, -0.16) | .291 | 64.14% |
| Middle Eastern | 3 (3) | -1.02 (-1.29, -0.74) | <.001 | 0.00% |
| South Asian | 2 (2) | -1.02 (-1.27, -0.77) | <.001 | 2.05% |

*‘NOS’ = not otherwise specified*

#### **Population group: East Asian**

Fourteen studies looked at interventions adapted for East Asian people. One study included 2 separate samples of East Asian participants, analysed separately based on their primary diagnosis of either anxiety or depression (Razali et al., 1998), so we treated the anxiety and depression samples individually in the analysis. Two studies targeted religious minorities who were of East Asian ethnicity (Razali et al., 2002, 1998). Most studies targeted depression (Cajanding, 2016; Choi et al., 2012; Choy & Lou, 2016; Gallagher-Thompson et al., 2007, 2010; Hwang et al., 2015; Muto et al., 2011; Razali et al., 1998); fewer targeted anxiety (An et al., 2020; Razali et al., 2002, 1998); psychosis (Bradley et al., 2006; Shin & Lukens, 2002; So et al., 2015). Eight of the studies compared an adapted intervention to an active control condition, while the other 6 compared the intervention to a waitlist or no intervention condition. The majority of the studies were adapted from CBT or cognitive therapy. Seven of the studies made therapist-related adaptations; all made at least one Content-related-level adaptation and 5 made Organisation-specific-level adaptations. Eight studies made adaptations to impact the therapeutic relationship, however, 3 of the studies were self-help or self-administered interventions (Choi et al., 2012; D. Gallagher-Thompson et al., 2010; Muto et al., 2011). All interventions included acceptability and suitability adaptations.

The results indicate that for East Asian people, adapted interventions were significantly more effective than non-adapted or waitlist controls (*K* = 14 (15 comparisons); Hedge’s *g* = -0.43 [95% CI: -0.71, -0.16, *p*=.002) and heterogeneity was substantial (*I^2^* = 78.55%).

#### **Population group: Latinx**

Twelve studies targeted Latinx (including Hispanic, Latina or Latino) populations. One study (Gonyea et al., 2016) included primary outcome measures for both anxiety and depression. The majority of the studies targeted depression (Ashing & Rosales, 2014; Beeber et al., 2010; Collado A., Calderon et al., 2016; Dahne et al., 2019; Dwight-Johnson et al., 2011; Gallagher-Thompson et al., 2008; Gonyea et al., 2016; Kanter et al., 2015). Anxiety was targeted in 2 studies (Feldman et al., 2016; Gonyea et al., 2016). One study each looked at eating disorder (specifically, binge eating disorder) (Cachelin et al., 2018); PTSD (Hinton et al., 2011) and mental health NOS (Alegría et al., 2019). All but two of the studies compared adapted interventions to waitlist controls. Eight studies adapted traditional CBT, 3 adapted behavioural activation and one adapted IPT. Ten studies made Therapist-related adaptations, all made Content-related adaptations and 8 made Organisation-specific adaptations. The most frequently applied specific adaptation types were explicit cultural (Content-related) and language translation (Therapist-related). Common factors adaptations were made to improve the therapeutic relationship in 5 studies whereas adaptations to improve acceptability and appropriateness were made in all but one of the studies.

A significant, medium effect size was observed in favour of adapted interventions for Latinx people compared to controls (*K*=12 (13 comparisons), Hedge’s *g* = -0.47 [95% CI: -0.85, -0.11], *p*=.0107). Heterogeneity was substantial: *I*^2^ = 86.92%.

#### **Population group: Refugees and/or asylum seekers**

Ten studies looked at adapted interventions for refugees or asylum seekers. Of these, the participant groups of 4 studies also belonged to another BME group (Hinton et al., 2004; Lindegaard et al., 2019, 2020; Meffert et al., 2014; Shaw et al., 2018). Most of the studies focussed on treating PTSD (Acarturk et al., 2016; Acarturk et al., 2015; Meffert et al., 2014; Neuner et al., 2008); one was transdiagnostic (de Graaff et al., 2020); the others targeted depression (Lindegaard et al., 2019, 2020; Shaw et al., 2018). Five of the studies included at least one explicit cultural-Content-related adaptation. The 3 studies that didn’t incorporate explicit cultural (Content-related) adaptations made Therapist-related-level adaptations (training for provider/facilitator and changed the provider of treatment to a layperson/non-professional). Three studies included adaptations of CBT; 2 were adaptations of EMDR; 1 provided adapted trauma counselling; 1 study adapted ACT; 1 adapted problem management, and 1 was adapted from IPT. Three RCTs compared adapted interventions to active treatments, the remainder compared adapted treatments to waitlist or no intervention/delayed intervention controls.

We observed a large effect of adapted interventions for refugees/asylum seekers compared to controls (*K*=10 (11 comparisons), Hedge’s *g* = -0.99 [95% CI: -1.35to -0.64], *p* <.0001); Heterogeneity was substantial: *I*^2^ = 81.98%.

#### **Population group: Black or mixed-race people**

Black or mixed-race people were targeted in 8 studies. Five studies targeted depression (Afuwape et al., 2010; Bolton et al., 2003; Glueckauf et al., 2012; Hendriks et al., 2020; Jones & Warner, 2011); 2 targeted anxiety (Carter et al., 2003; Hendriks et al., 2020); 1 looked at mental health problems NOS (Bonilla-Escobar et al., 2018) and the remaining study targeted PTSD (Meffert et al., 2014). The study by Hendriks and colleagues (2020) included outcome measures for depression and anxiety symptoms. All but one of the studies compared adapted interventions to waitlist controls. Five of the studies used an adaptation of CBT, 1 utilised positive psychology and 2 adapted IPT. Therapist-related adaptations were made in all but one of the studies, Content-related adaptations in 5 studies and Organisation-specific adaptations in all 8 studies. The most frequently applied specific adaptation types were Therapist-related (provider of treatment) and Organisation-specific (the medium used to provide treatment).

A significant, medium effect size was observed in favour of adapted interventions compared to controls (*K*=8 (10 comparisons); Hedge’s *g*= -0.70 [95% CI: -0.95, =-0.46], *p*<.0001). Heterogeneity was substantial: *I*^2^ = 62.59%.

#### **Population group: BME ‘not otherwise specified’, immigrants/migrants or mixed groups**

Five of the included RCTs were of BME groups that were not explicitly defined (e.g., ‘immigrants/migrants’ with little further detail) or were of mixed ethnic groups. Four studies targeted depression (including 1 for perinatal depression) (Grote et al., 2009; Laperriere et al., 2005; Lindegaard et al., 2020; Scogin et al., 2007) and the other targeted schizophrenia (Rathod et al., 2013). All but one compared adapted interventions to active controls. Only 1 study included therapist-related adaptations, whilst all included Content-related adaptations. Four included organisation- specific adaptations. Only 1 study made adaptations to influence therapeutic relationship whilst all made adaptations to improve acceptability and suitability of treatment. The most frequently applied specific adaptation was explicit cultural adaptation, which were made across all 5 studies. Almost half of the studies provided training to the provider, the location of treatment Therapist-related and the medium used to provide treatment. Four studies adapted CBT and the other adapted IPT.

We observed significant medium effect size in favour of adapted interventions compared to controls (*K*=5; Hedge’s *g*=-0.62 [95% CI: -1.04, -0.19], *p*=.004). Heterogeneity was considerable: *I*^2^ = 71.14%.

#### **Population group: South Asian**

Only 2 studies looked at South Asian communities. One study targeted schizophrenia (Naeem et al., 2015) and used an active control and the other targeted depression (Naeem et al., 2014) and used a waitlist/no intervention control. Neither study made Therapist-related adaptations or organisation-specific adaptations to interventions. Both made content-related adaptations and these were all exclusively and explicitly described as cultural. Both incorporated religious adaptations and neither made adaptations explicitly to influence the therapeutic relationship. Both of the interventions were adaptations of CBT.

A very large effect size was observed (*K* = 2; Hedge’s *g* = -1.02 [95% CI: -1.27, -0.77], *p*<.0001) and there was minimal heterogeneity (*I^2^* =2.05%).

#### **Population group: Religious minority**

Religious minority groups were the focus of 3 studies; one which used 2 separate participant samples for anxiety and depression (Razali et al., 1998), and 2 which targeted anxiety specifically (Razali et al., 2002; Rosmarin et al., 2010). All but one of the studies were adapted from CBT; these studies also all compared the adapted CBT intervention to an active control whilst the other used a waitlist control group. The other study adapted loosely defined ‘psychotherapy’ None of the studies made Therapist-related adaptations, yet all made Content-related adaptations. Three of the studies included explicit modifications to influence the therapeutic relationship and all made adaptations to improve acceptability and appropriateness (all of which were described as explicitly cultural). Specific religious adaptations were made in all studies.

We observed a very small effect size in favour of adapted interventions for religious minority groups compared to controls (*K*=3 (4 comparisons); Hedge’s *g* = -0.18 [95% CI: -0.52, -0.16], *p*= .2907) but this was not significant. Heterogeneity was substantial: *I*^2^ = 63.14%.

#### **Population group: Middle Eastern**

Three of the studies targeted people from Middle Eastern backgrounds; 1 targeted people with PTSD (Knaevelsrud et al., 2015) and the others depression (Lindegaard et al., 2019; Shaw et al., 2018). All compared adapted CBT interventions to waitlist controls. Adaptations made to all studies included Therapist-related adaptations and Content-related adaptations. Two of the studies also made Organisation-specific adaptations. Common factors adaptations included acceptability and appropriateness adaptations made in all studies, while only one study made modifications to impact the therapeutic relationship. All studies made explicitly described cultural adaptions.

A meta-analysis showed a very large effect size in favour of adapted interventions compared to waitlist controls (*K*=3); Hedge’s *g*= -1.02 [95% CI: -1.29, -0.74], *p*<.0001. There was no heterogeneity observed: *I*^2^ = 0.00%.

## **Meta-regressions**

### All RCTs

#### **Control type**

**Table F.4: Single predictor meta-regression - control group type**

| **K** | **Variable** | **Coefficient** | **SE** | **p-value** | **95% CI** | **R^2** |
| --- | --- | --- | --- | --- | --- | --- |
| 56 | Waitlist/no intervention ^a^ | -0.4171 | 0.1315 | .002 | -0.67, -0.16 | 19.77% |

*^a^ reference category = active control*

#### **Mental health problem**

**Table F.5: Mental health problem meta-regressions**

| **K** | **Model** | **Variable** | **Coefficient** | **SE** | **p-value** | **95% CI** | **R^2** |
| --- | --- | --- | --- | --- | --- | --- | --- |
| 56 | 1 | Target problem ^a^ |  |  |  |  |  |
|  |  | Depression | -0.1328 | 0.2136 | .5340 | -0.55, 0.28 | 0.00% |
|  |  | PTSD | -0.6446 | 0.3023 | .033 | -1.24, -0.05 |  |
|  |  | Psychosis | -0.0544 | 0.3152 | .8628 | -0.56, 0.67 |  |
|  |  | Eating disorder | -0.6780 | 0.6215 | .2753 | -1.90, 0.54 |  |
|  |  | MH problem NOS | -0.0533 | 0.2977 | .8579 | -0.64, 0.53 |  |
| 56 | 2 | Target problem ^a^ |  |  |  |  |  |
|  |  | Depression | -0.1414 | 0.1982 | .4755 | -0.53, 0.25 | 6.96% |
|  |  | PTSD | -0.5570 | 0.2823 | .049 | -1.11, -0.00 |  |
|  |  | Psychosis | -0.0451 | 0.2951 | .8785 | -0.62, 0.53 |  |
|  |  | Eating disorder | -0.4779 | 0.5874 | .416 | -1.63, 0.67 |  |
|  |  | MH problem NOS | -0.0132 | 0.2743 | .9618 | -0.55, 0.52 |  |
|  |  | Waitlist/no intervention ^b^ | -0.3690 | 0.1434 | .010 | -0.65, -0.09 |  |

*^a^ reference category = anxiety; b reference category = active control; MH = mental health; NOS = not otherwise specified; PTSD = post-traumatic stress disorder*

#### **Risk of bias**

***Table F.6: Single predictor meta-regression – risk of bias***

| **K** | **Variable** | **Coefficient** | **SE** | **p-value** | **95% CI** | **R^2** |
| --- | --- | --- | --- | --- | --- | --- |
| 56 | Low risk of bias ^a^ | 0.2793 | 0.1508 | .064 | -0.57, 0.02 | 12.21% |

*^a^ reference category = unclear risk of bias*

#### **Overarching adaptation area**

**Table F.7: Single predictor meta-regression- therapist-related adaptations**

| **K** | **Variable** | **Coefficient** | **SE** | **p-value** | **95% CI** | **R^2** |
| --- | --- | --- | --- | --- | --- | --- |
| 56 | Therapist-related adaptation ^a^ | -0.0707 | 0.1485 | .664 | -0.22, 0.36 | 0.00% |

*^a^ reference category = no therapist-related adaptation*

**Table F.8: Single predictor meta-regression- Content-related-related adaptations**

| **K** | **Variable** | **Coefficient** | **SE** | **p-value** | **95% CI** | **R^2** |
| --- | --- | --- | --- | --- | --- | --- |
| 51 | Content-related-related adaptation ^a^ | -0.2410 | 0.2486 | .332 | -0.73, 0.25 | 0.00% |

*^a^ reference category = no Content-related-related adaptation*

**Table F.9: Single predictor meta-regression- organisation-specific adaptations**

| **K** | **Variable** | **Coefficient** | **SE** | **p-value** | **95% CI** | **R^2** |
| --- | --- | --- | --- | --- | --- | --- |
| 56 | Organisation-specific adaptation ^a^ | -0.3692 | 0.1425 | .009 | -0.65, -0.09 | 7.29% |

*^a^ reference category = no organisation-specific adaptation*

**Table F.10: Overarching adaptation areas meta-regressions**

| **K** | **Model** | **Variable** | **Coefficient** | **SE** | **p-value** | **95% CI** | **R^2** |
| --- | --- | --- | --- | --- | --- | --- | --- |
| 56 | 1 | Overarching adaptation area |  |  |  |  |  |
|  |  | Therapist-related adaptation ^a^ | 0.1089 | 0.1474 | .459 | -0.18, 0.49 | 6.98% |
|  |  | Content-related-related adaptation ^b^ | -0.2626 | 0.2458 | .285 | -0.74, 0.22 |  |
|  |  | Organisation-specific adaptation ^c^ | -0.3995 | 0.1448 | .006 | -0.68, -0.12 |  |
| 56 | 2 | Overarching adaptation area |  |  |  |  |  |
|  |  | Therapist-related adaptation ^a^ | 0.1121 | 0.1333 | .401 | -0.15, 0.37 | 30.50% |
|  |  | Content-related-related adaptation ^b^ | -0.3460 | 0.2215 | .118 | -0.78, 0.09 |  |
|  |  | Organisation-specific adaptation ^c^ | -0.3401 | 0.1316 | .009 | -0.60, -0.08 |  |
|  |  | Waitlist/no intervention ^d^ | -0.3977 | 0.1274 | .002 | -0.65, -0.15 |  |

*^a^ reference category = no therapist-related adaption; ^b^ reference category = no Content-related-related adaption; ^c^ reference category = no organisation-specific adaption; ^d^ reference category = active control*

#### **Specific adaptation type**

**Table F.11: Single predictor meta-regression – explicit cultural (Content-related) adaptation**

| **K** | **Variable** | **Coefficient** | **SE** | **p-value** | **95% CI** | **R^2** |
| --- | --- | --- | --- | --- | --- | --- |
| 56 | Explicit cultural adaptation ^a^ | -0.1374 | 0.2012 | .495 | -0.53, 0.26 | 0.00% |

*^a^ reference category = no explicit cultural (Content-related) adaptation*

***Table F.12: Single predictor meta-regression- form used to provide treatment adaptation***

| **K** | **Variable** | **Coefficient** | **SE** | **p-value** | **95% CI** | **R^2** |
| --- | --- | --- | --- | --- | --- | --- |
| 56 | Form used adaptation ^a^ | -0.2031 | 0.1503 | .177 | -0.49, 0.09 | 0.00% |

*^a^ reference category = no form used adaptation*

***Table F.13: Single predictor meta-regression -* *language translation adaptation***

| **K** | **Variable** | **Coefficient** | **SE** | **p-value** | **95% CI** | **R^2** |
| --- | --- | --- | --- | --- | --- | --- |
| 56 | Language translation adaptation ^a^ | 0.0675 | 0.1457 | .643 | -0.22, 0.35 | 0.00% |

*^a^ reference category = no language translation adaptation*

**Table F.13: Single predictor meta-regression – provider of treatment adaptation**

| **K** | **Variable** | **Coefficient** | **SE** | **p-value** | **95% CI** | **R^2** |
| --- | --- | --- | --- | --- | --- | --- |
| 56 | Provider of treatment adaptation ^a^ | -0.0749 | 0.1597 | .639 | -0.39, 0.24 | 0.00% |

*^a^ reference category = no provider of treatment adaptation*

***Table F.14: Single predictor meta-regression- training for provider***

| **K** | **Variable** | **Coefficient** | **SE** | **p-value** | **95% CI** | **R^2** |
| --- | --- | --- | --- | --- | --- | --- |
| 56 | Training for provider adaptation ^a^ | 0.2269 | 0.1476 | .124 | -0.06, 0.52 | 0.42% |

*^a^ reference category = no training for provider adaptation*

***Table F.15: Single predictor meta-regression –* *location of treatment***

| **K** | **Variable** | **Coefficient** | **SE** | **p-value** | **95% CI** | **R^2** |
| --- | --- | --- | --- | --- | --- | --- |
| 56 | Location of treatment adaptation ^a^ | -0.0654 | 0.1640 | .690 | -0.39, 0.26 | 0.00% |

*^a^ reference category = no location of treatment adaptation*

***Table F.14:*** ***Specific adaptation type meta-regressions***

| **K** | **Model** | **Variable** | **Coefficient** | **SE** | **p-value** | **95% CI** | **R^2** |
| --- | --- | --- | --- | --- | --- | --- | --- |
| 56 | 1 | Specific adaptation type |  |  |  |  |  |
|  |  | Explicit cultural | -0.1540 | 0.2360 | .514 | -0.62, 0.31 | 0.00% |
|  |  | Language translation | -0.0309 | 0.1574 | .844 | -0.28, 0.34 |  |
|  |  | Form used | -0.1315 | 0.1669 | .431 | -0.46, 0.20 |  |
|  |  | Provider of treatment | -0.2418 | 0.2136 | .256 | -0.66, 0.18 |  |
|  |  | Training for provider | 0.3166 | 0.1841 | .085 | -0.00, 0.68 |  |
|  |  | Location of treatment | -0.0832 | 0.1813 | .646 | -0.44, 0.28 |  |
| 56 | 2 | Specific adaptation type |  |  |  |  |  |
|  |  | Explicit cultural | -0.1826 | 0.2164 | .398 | -0.61, 0.24 | 6.91% |
|  |  | Language translation | -0.0002 | 0.1436 | .999 | -0.28, 0.28 |  |
|  |  | Form used | -0.1375 | 0.1522 | .366 | -0.44, 0.16 |  |
|  |  | Provider of treatment | -0.1692 | 0.1967 | .389 | -0.55, 0.22 |  |
|  |  | Training for provider | 0.2694 | 0.1684 | .109 | -0.06, 0.60 |  |
|  |  | Location of treatment | -0.1350 | 0.1658 | .416 | -0.46, 0.19 |  |
|  |  | Waitlist/no intervention ^a^ | -0.4099 | 0.1417 | .004 | -0.69, -0.13 |  |

*^a^ reference category = active control; Language translation (therapist and Content-related-related) adaptations; form used = form used to provide treatment*

#### **Common factors adaptations**

***Table F.16: Single predictor meta-regression – therapeutic relationship adaptations***

| **K** | **Variable** | **Coefficient** | **SE** | **p-value** | **95% CI** | **R^2** |
| --- | --- | --- | --- | --- | --- | --- |
| 56 | Therapeutic relationship | 0.0339 | 0.1450 | .820 | -0.25, 0.32 | 0.00% |

*^a^ reference category= no therapeutic relationship adaptation*

***Table F.16: Single predictor meta-regression - acceptability and appropriateness adaptations***

| **K** | **Variable** | **Coefficient** | **SE** | **p-value** | **95% CI** | **R^2** |
| --- | --- | --- | --- | --- | --- | --- |
| 56 | Acceptability and suitability ^a^ | 0.2130 | 0.3827 | .578 | -0.54, 0.96 | 0.00% |

*^a^ reference category= no acceptability and suitability* *adaptation*

***Table F.17: Common factors adaptations meta-regressions***

| **K** | **Model** | **Variable** | **Coefficient** | **SE** | **p-value** | **95% CI** | **R^2** |
| --- | --- | --- | --- | --- | --- | --- | --- |
| 56 | 1 | Common factors |  |  |  |  |  |
|  |  | Therapeutic relationship | -0.0191 | 0.1489 | .897 | -0.31, 0.27 | 0.00% |
|  |  | Acceptability and suitability | -0.2059 | 0.3941 | .601 | -0.98, -0.40 |  |
| 56 | 2 | Common factors |  |  |  |  |  |
|  |  | Therapeutic relationship | -0.0505 | 0.1373 | .713 | -0.22, 0.32 | 16.21% |
|  |  | Acceptability and suitability | -0.3031 | 0.3715 | .415 | -1.03, 0.42 |  |
|  |  | Waitlist/no intervention ^a^ | -0.4314 | 0.1356 | .002 | -0.70, -0.17 |  |

*^a^ reference category = active control*

### **RCTs of adapted CBT interventions for anxiety and depression**

#### **Common factors adaptations**

**Table F.18: Common factors adaptations meta-regressions**

| **K** | **Model** | **Variable** | **Coefficient** | **SE** | **p-value** | **95% CI** | **R^2** |
| --- | --- | --- | --- | --- | --- | --- | --- |
| 27 | 1 | Common factors |  |  |  |  |  |
|  |  | Therapeutic relationship | -0.1038 | 0.2531 | .6818 | -0.60, 0.39 | 0.00% |
|  |  | Acceptability and suitability | -0.3327 | 0.8839 | .7067 | -2.07,1.40 |  |

#### **Specific adaptation type**

**Table F.19: Specific adaptation type meta-regressions**

| **K** | **Model** | **Variable** | **Coefficient** | **SE** | **p-value** | **95% CI** | **R^2** |
| --- | --- | --- | --- | --- | --- | --- | --- |
| 27 | 1 | Specific adaptation type |  |  |  |  |  |
|  |  | Explicit cultural | -0.0187 | 0.5587 | .973 | -1.18, -0.20 | 0.97% |
|  |  | Language – Content-related-related | 0.2779 | 0.3282 | .397 | -0.37, 0.92 |  |
|  |  | Form used | 0.3151 | 0.2676 | .239 | -0.21, 0.84 |  |
|  |  | Language – therapist-related | -0.4174 | 0.2934 | .155 | -0.99, 0.16 |  |

*Language – Content-related = language translation (Content-related-related) adaptations; language – therapist-related = language translation (therapist-related) adaptations; form used = form used to provide treatment*

# References

Acarturk, C., Konuk, E., Cetinkaya, M., Senay, I., Sijbrandij, M., Cuijpers, P., & Aker, T. (2015). EMDR for Syrian refugees with posttraumatic stress disorder symptoms: Results of a pilot randomized controlled trial. *European Journal of Psychotraumatology*, *6*. Retrieved from http://ovidsp.ovid.com/ovidweb.cgi?T=JS&PAGE=reference&D=psyc12&NEWS=N&AN=2015-26838-001

Acarturk, C., Konuk, E., Cetinkaya, M., Senay, I., Sijbrandij, M., Gulen, B., & Cuijpers, P. (2016). The efficacy of eye movement desensitization and reprocessing for post-traumatic stress disorder and depression among Syrian refugees: Results of a randomized controlled trial. *Psychological Medicine*, *46*(12), 2583–2593.

Acarturk, Ceren, Konuk, E., Cetinkaya, M., Senay, I., Sijbrandij, M., Cuijpers, P., & Aker, T. (2015). EMDR for Syrian refugees with posttraumatic stress disorder symptoms: Results of a pilot randomized controlled trial. *European Journal of Psychotraumatology*, *6*. Retrieved from http://ovidsp.ovid.com/ovidweb.cgi?T=JS&PAGE=reference&D=psyc12&NEWS=N&AN=2015-26838-001

Afuwape, S. A., Craig, T. K., Harris, T., Clarke, M., Flood, A., Olajide, D., … Thornicroft, G. (2010). The Cares of Life Project (CoLP): An exploratory randomised controlled trial of a community-based intervention for black people with common mental disorder. *Journal of Affective Disorders*, *127*(1), 370–374.

Alavi, N., & Hirji, A. (2020). The Efficacy of PowerPoint-based CBT Delivered Through Email: Breaking the Barriers to Treatment for Generalized Anxiety Disorder. *Journal of Psychiatric Practice*, *26*(2), 89–100.

Alegría, M., Falgas-Bague, I., Collazos, F., Carmona Camacho, R., Lapatin Markle, S., Wang, Y., … Mueser, K. T. (2019). Evaluation of the Integrated Intervention for Dual Problems and Early Action Among Latino Immigrants With Co-occurring Mental Health and Substance Misuse Symptoms: A Randomized Clinical Trial. *JAMA Network Open*, *2*(1), e186927–e186927.

Alegria, M., Ludman, E., Kafali, E. N., Lapatin, S., Vila, D., Shrout, P. E., … Canino, G. (2014). Effectiveness of the Engagement and Counseling for Latinos (ECLA) intervention in low-income Latinos. *Medical Care*, *52*(11), 989–997.

Alegría, M., Ludman, E., Kafali, E. N., Lapatin, S., Vila, D., Shrout, P. E., … Canino, G. (2014). Effectiveness of the Engagement and Counseling for Latinos (ECLA) intervention in low-income Latinos. *Medical Care*, *52*(11), 989–997.

An, Q., Wang, K., Sun, F., & Zhang, A. (2020). The effectiveness of modified, group-based CBT for dementia worry among Chinese elders. *Journal of Affective Disorders*, *274*, 76–84. https://doi.org/10.1016/j.jad.2020.05.054

Antoniades, J., Mazza, D., & Brijnath, B. (2014). Efficacy of depression treatments for immigrant patients: Results from a systematic review. *BMC Psychiatry*, *14*, 176.

Ashing, K., & Rosales, M. (2014). A telephonic-based trial to reduce depressive symptoms among Latina breast cancer survivors. *Psycho-Oncology*, *23*(5), 507‐515.

Bedoya, C. A., Traeger, L., Trinh, N. H., Chang, T. E., Brill, C. D., Hails, K., … Yeung, A. (2014). Impact of a culturally focused psychiatric consultation on depressive symptoms among Latinos in primary care. *Psychiatric Services (Washington, D.C.)*, *65*(10), 1256‐1262.

Beeber, L. S., Holditch-Davis, D., Perreira, K., Schwartz, T. A., Lewis, V., Blanchard, H., … Davis Goldman, B. (2010). Short-term in-home intervention reduces depressive symptoms in Early Head Start Latina mothers of infants and toddlers. *Research in Nursing & Health*, *33*(1), 60–76.

Benish, Quintana, & Wampold, B. E. (2011). Culturally adapted psychotherapy and the legitimacy of myth: A direct-comparison meta-analysis. *Journal of Counseling Psychology*, *58*(3), 279–289. https://doi.org/10.1037/a0023626

Benuto, L. T., & O’Donohue, W. (2015). Is culturally sensitive cognitive behavioral therapy an empirically supported treatment?: The case for Hispanics. *International Journal of Psychology & Psychological Therapy*, *15*(3), 405–421.

Bernardi, J., Dahiya, M., & Jobson, L. (2019). Culturally modified cognitive processing therapy for Karen refugees with posttraumatic stress disorder: A pilot study. *Clinical Psychology & Psychotherapy*, *26*(5), 531–539.

Bernstein, K., Park, S. Y., Hahm, S., Lee, Y. N., Seo, J. Y., & Nokes, K. M. (2016). Efficacy of a Culturally Tailored Therapeutic Intervention Program for Community Dwelling Depressed Korean American Women: A Non-Randomized Quasi-Experimental Design Study. *Archives of Psychiatric Nursing*, *30*(1), 19–26.

Bhui, K., Aslam, R. W., Palinski, A., McCabe, R., Johnson, M. R. D., Weich, S., … Szczepura, A. (2015). Interventions designed to improve therapeutic communications between black and minority ethnic people and professionals working in psychiatric services: A systematic review of the evidence for their effectiveness. *Health Technology Assessment (Winchester, England)*, *19*(31), vii–173.

Bolton, P., Bass, J., Neugebauer, R., Verdeli, H., Clougherty, K. F., Wickramaratne, P., … Weissman, M. (2003). Group interpersonal psychotherapy for depression in rural Uganda: A randomized controlled trial. *JAMA*, *289*(23), 3117–3124. https://doi.org/10.1001/jama.289.23.3117

Bonilla-Escobar, F. J., Fandino-Losada, A., Martinez-Buitrago, D. M., Santaella-Tenorio, J., Tobon-Garci, D., Munoz-Morales, E. J., … Bolton, P. (2018). Randomized controlled trial of a transdiagnostic cognitive-behavioral intervention for Afro-descendants’ survivors of systemic violence in Colombia. *PLoS ONE*, *13*(12). Retrieved from http://ovidsp.ovid.com/ovidweb.cgi?T=JS&PAGE=reference&D=psyc14&NEWS=N&AN=2018-63843-001

Bradley, G. M., Couchman, G. M., Perlesz, A., Nguyen, A. T., Singh, B., & Riess, C. (2006). Multiple-family group treatment for English- and Vietnamese-speaking families living with schizophrenia. *Psychiatric Services (Washington, D.C.)*, *57*(4), 521–530.

Cabassa, L. J., & Hansen, M. C. (2007). A systematic review of depression treatments in primary care for Latino adults. *Research on Social Work Practice*, *17*(4), 494–503.

Cachelin, F. M., Gil-Rivas, V., Palmer, B., Vela, A., Phimphasone, P., de Hernandez, B. U., & Tapp, H. (2018). Randomized controlled trial of a culturally-adapted program for Latinas with binge eating. *Psychological Services*, No-Specified.

Cajanding, R. J. M. (2016). The Effectiveness of a Nurse-Led Cognitive-Behavioral Therapy on the Quality of Life, Self-Esteem and Mood Among Filipino Patients Living With Heart Failure: A Randomized Controlled Trial. *Applied Nursing Research*, *31*, 86–93.

Camacho, Á., González, P., Castañeda, S. F., Simmons, A., Buelna, C., Lemus, H., & Talavera, G. A. (2015). Improvement in Depressive Symptoms Among Hispanic/Latinos Receiving a Culturally Tailored IMPACT and Problem-Solving Intervention in a Community Health Center. *Community Mental Health Journal*, *51*(4), 385–392. https://doi.org/10.1007/s10597-014-9750-7

Carter, M. M., Sbrocco, T., Gore, K. L., Marin, N. W., & Lewis, E. L. (2003). Cognitive–Behavioral Group Therapy Versus a Wait-List Control in the Treatment of African American Women with Panic Disorder. *Cognitive Therapy and Research*, *27*(5), 505–519.

Chien, W.-T., Leung, S.-F., & Sk Chu, C. (2012). A nurse-led, needs-based psycho-education intervention for Chinese patients with first-onset mental illness. *Contemporary Nurse: A Journal for the Australian Nursing Profession*, *40*(2), 194–207.

Choi, I., Zou, J., Titov, N., Dear, B. F., Li, S., Johnston, L., … Hunt, C. (2012). Culturally attuned Internet treatment for depression amongst Chinese Australians: A randomised controlled trial. *Journal of Affective Disorders*, *136*(3), 459–468. https://doi.org/10.1016/j.jad.2011.11.003

Chowdhary, N., Jotheeswaran, A. T., Nadkarni, A., Hollon, S. D., King, M., Jordans, M. J. D., … Patel, V. (2014). The methods and outcomes of cultural adaptations of psychological treatments for depressive disorders: A systematic review. *Psychological Medicine*, *44*(6), 1131–1146.

Choy, J. C. P., & Lou, V. W. Q. (2016). Effectiveness of the Modified Instrumental Reminiscence Intervention on Psychological Well-Being Among Community-Dwelling Chinese Older Adults: A Randomized Controlled Trial. *The American Journal of Geriatric Psychiatry : Official Journal of the American Association for Geriatric Psychiatry*, *24*(1), 60–69.

Collado A., Calderon M., MacPherson L., & Lejuez C. (2016). The Efficacy of Behavioral Activation Treatment among Depressed Spanish-Speaking Latinos. *Journal of Consulting and Clinical Psychology*, *84*(7), 651–657. https://doi.org/10.1037/ccp0000103

Collado, A., Castillo, S. D., Maero, F., Lejuez, C. W., & Macpherson, L. (2014). Pilot of the brief behavioral activation treatment for depression in latinos with limited english proficiency: Preliminary evaluation of efficacy and acceptability. *Behavior Therapy*, *45*(1), 102–115.

Collado A., Lim A.C., & MacPherson L. (2016). A systematic review of depression psychotherapies among Latinos. *Clinical Psychology Review*, *45*((Collado) Emory University, Department of Psychiatry and Behavioral Sciences, 12 Executive Park Drive NE, suite 300, Atlanta, GA 30329, United States), 193–209. https://doi.org/10.1016/j.cpr.2016.04.001

Comas-Díaz, L. (1981). Effects of cognitive and behavioral group treatment on the depressive symptomatology of Puerto Rican women. *Journal of Consulting and Clinical Psychology*, *49*(5), 627‐632.

Cooper, L. A., Ghods Dinoso, B. K., Ford, D. E., Roter, D. L., Primm, A. B., Larson, S. M., … Wang, N.-Y. (2013). Comparative effectiveness of standard versus patient-centered collaborative care interventions for depression among African Americans in primary care settings: The BRIDGE Study. *Health Services Research*, *48*(1), 150–174.

Dahne, J., Collado, A., Lejuez, C. W., Risco, C. M., Diaz, V. A., Coles, L., … Carpenter, M. J. (2019). Pilot randomized controlled trial of a Spanish-language Behavioral Activation mobile app (Aptivate!) for the treatment of depressive symptoms among united states Latinx adults with limited English proficiency. *Journal of Affective Disorders*, *250*, 210–217.

de Graaff, A. M., Cuijpers, P., McDaid, D., Park, A., Woodward, A., Bryant, R. A., … Sijbrandij, M. (2020). Peer-provided Problem Management Plus (PM+) for adult Syrian refugees: A pilot randomised controlled trial on effectiveness and cost-effectiveness. *Epidemiology and Psychiatric Sciences*, *29*. https://doi.org/10.1017/S2045796020000724

Degnan, A., Baker, S., Edge, D., Nottidge, W., Noke, M., Press, C. J., … Drake, R. J. (2018). The nature and efficacy of culturally-adapted psychosocial interventions for schizophrenia: A systematic review and meta-analysis. *Psychological Medicine*, *48*(5), 714–727.

Drozdek, B., Kamperman, A. M., Bolwerk, N., Tol, W. A., & Kleber, R. J. (2012). Group therapy with male asylum seekers and refugees with posttraumatic stress disorder: A controlled comparison cohort study of three day-treatment programs. *The Journal of Nervous and Mental Disease*, *200*(9), 758–765.

Dwight-Johnson, M., Aisenberg, E., Golinelli, D., Hong, S., O’Brien, M., & Ludman, E. (2011). Telephone-based cognitive-behavioral therapy for Latino patients living in rural areas: A randomized pilot study. *Psychiatric Services (Washington, D.C.)*, *62*(8), 936–942.

Escobar, K. M., & Gorey, K. M. (2018). Cognitive behavioral interventions for depression among Hispanic people: Promising meta-analytic evidence for deep cultural adaptations. *Social Work in Mental Health*, *16*(6), 746–758.

Feldman, J. M., Matte, L., Interian, A., Lehrer, P. M., Lu, S. E., Scheckner, B., … al, et. (2016). Psychological treatment of comorbid asthma and panic disorder in Latino adults: Results from a randomized controlled trial. *Behaviour Research and Therapy*, *87*, 142‐154.

Gallagher-Thompson, D., Gray, H. L., Tang, P. C., Pu, C. Y., Leung, L. Y., Wang, P. C., … al, et. (2007). Impact of in-home behavioral management versus telephone support to reduce depressive symptoms and perceived stress in Chinese caregivers: Results of a pilot study. *American Journal of Geriatric Psychiatry*, *15*(5), 425‐434.

Gallagher-Thompson, D., Wang, P. C., Liu, W., Cheung, V., Peng, R., China, D., & Thompson, L. W. (2010). Effectiveness of a psychoeducational skill training DVD program to reduce stress in Chinese American dementia caregivers: Results of a preliminary study. *Aging & Mental Health*, *14*(3), 263–273.

Gallagher-Thompson, Dolores, Gray, H. L., Dupart, T., Jimenez, D., & Thompson, L. W. (2008). Effectiveness of cognitive/behavioral small group intervention for reduction of depression and stress in non-Hispanic White and Hispanic/Latino women dementia family caregivers: Outcomes and mediators of change. *Special Issue: Psychotherapy with Older People*, *26*(4), 286–303.

Gearing, R. E., Schwalbe, C. S., MacKenzie, M. J., Brewer, K. B., Ibrahim, R. W., Olimat, H. S., … Al-Krenawi, A. (2013). Adaptation and translation of mental health interventions in Middle Eastern Arab countries: A systematic review of barriers to and strategies for effective treatment implementation. *International Journal of Social Psychiatry*, *59*(7), 671–681.

Glueckauf, R. L., Davis, W. S., Willis, F., Sharma, D., Gustafson, D. J., Hayes, J., … al, et. (2012). Telephone-based, cognitive-behavioral therapy for African American dementia caregivers with depression: Initial findings. *Rehabilitation Psychology*, *57*(2), 124‐139.

Gonyea, J. G., Lopez, L. M., & Velasquez, E. H. (2016). The Effectiveness of a Culturally Sensitive Cognitive Behavioral Group Intervention for Latino Alzheimer’s Caregivers. *The Gerontologist*, *56*(2), 292–302.

Griner, D., & Smith, T. B. (2006). Culturally adapted mental health intervention: A meta-analytic review. *Special Issue: Culture, Race, and Ethnicity in Psychotherapy*, *43*(4), 531–548.

Grote, N. K., Swartz, H. A., Geibel, S. L., Zuckoff, A., Houck, P. R., & Frank, E. (2009). A randomized controlled trial of culturally relevant, brief interpersonal psychotherapy for perinatal depression. *Psychiatric Services*, *60*(3), 313–321.

Habib, N., Dawood, S., Kingdon, D., & Naeem, F. (2015). Preliminary evaluation of culturally adapted CBT for psychosis (CA-CBTp): Findings from developing culturally-sensitive CBT project (DCCP). *Behavioural & Cognitive Psychotherapy*, *43*(2), 200–208.

Hahm, H. C., Hsi, J. H., Petersen, J. M., Xu, J., Lee, E. A., Chen, S. H., & Liu, C. H. (2020). Preliminary efficacy of AWARE in college health service centers: A group psychotherapy intervention for Asian American women. *Journal of American College Health*, *0*(0), 1–5. https://doi.org/10.1080/07448481.2020.1777135

Hahm, H. C., Zhou, L., Lee, C., Maru, M., Petersen, J. M., & Kolaczyk, E. D. (2019). Feasibility, preliminary efficacy, and safety of a randomized clinical trial for Asian Women’s Action for Resilience and Empowerment (AWARE) intervention. *American Journal of Orthopsychiatry*, *89*(4), 462–474.

Hall, G. C. N., Ibaraki, A. Y., Huang, E. R., Marti, C. N., & Stice, E. (2016). A Meta-Analysis of Cultural Adaptations of Psychological Interventions. *Behavior Therapy*, *47*(6), 993–1014.

Hankerson, S. H., & Weissman, M. M. (2012). Church-based health programs for mental disorders among African Americans: A review. *Psychiatric Services (Washington, D.C.)*, *63*(3), 243–249.

Healey, P., Stager, M. L., Woodmass, K., Dettlaff, A. J., Vergara, A., Janke, R., & Wells, S. J. (2017). Cultural adaptations to augment health and mental health services: A systematic review. *BMC Health Services Research*, *17*, 1–26.

Heilemann, M. V., Pieters, H. C., Kehoe, P., & Yang, Q. (2011). Schema therapy, motivational interviewing, and collaborative-mapping as treatment for depression among low income, second generation Latinas. *Journal of Behavior Therapy and Experimental Psychiatry*, *42*(4), 473–480.

Hendriks, T., Schotanus-Dijkstra, M., Hassankhan, A., Sardjo, W., Graafsma, T., Bohlmeijer, E., & de Jong, J. (2020). Resilience and well-being in the Caribbean: Findings from a randomized controlled trial of a culturally adapted multi-component positive psychology intervention. *Journal of Positive Psychology*, *15*(2), 238–253.

Himelhoch, S., Mohr, D., Maxfield, J., Clayton, S., Weber, E., Medoff, D., & Dixon, L. (2011). Feasibility of telephone-based cognitive behavioral therapy targeting major depression among urban dwelling African-American people with co-occurring HIV. *Psychology, Health & Medicine*, *16*(2), 156–165.

Hinton, D. E., Chhean, D., Pich, V., Safren, S. A., Hofmann, S. G., & Pollack, M. H. (2005). A randomized controlled trial of cognitive-behavior therapy for Cambodian refugees with treatment-resistant PTSD and panic attacks: A cross-over design. *Journal of Traumatic Stress*, *18*(6), 617–629.

Hinton, D. E., Hofmann, S. G., Rivera, E., Otto, M. W., & Pollack, M. H. (2011). Culturally adapted CBT (CA-CBT) for Latino women with treatment-resistant PTSD: a pilot study comparing CA-CBT to applied muscle relaxation. *Behaviour Research and Therapy*, *49*(4), 275–280.

Hinton, D. E., Pham, T., Tran, M., Safren, S. A., Otto, M. W., & Pollack, M. H. (2004). CBT for Vietnamese refugees with treatment-resistant PTSD and panic attacks: A pilot study. *Journal of Traumatic Stress*, *17*(5), 429–433. https://doi.org/10.1023/B:JOTS.0000048956.03529.fa

Hovey, J. D., Hurtado, G., & Seligman, L. D. (2014). Findings for a CBT Support Group for Latina Migrant Farmworkers in Western Colorado. *Current Psychology*, *33*(3), 271–281. https://doi.org/10.1007/s12144-014-9212-y

Huey Jr, S. J., & Pan, D. (2006). Culture-responsive one-session treatment for phobic Asian Americans: A pilot study. Special issue: Culture, race, and ethnicity in psychotherapy. *Psychotherapy*, *43*(4), 549–554.

Huey, S. J., & Tilley, J. L. (2018). Effects of mental health interventions with Asian Americans: A review and meta-analysis. *Journal of Consulting and Clinical Psychology*, *86*(11), 915–930.

Hwang, W. C., Myers, H. F., Chiu, E., Mak, E., Butner, J. E., Fujimoto, K., … Miranda, J. (2015). Culturally adapted cognitive-behavioral therapy for Chinese Americans with depression: A randomized controlled trial. *Psychiatric Services*, *66*(10), 1035–1042.

Interian, A., Allen, L. A., Gara, M. A., & Escobar, J. I. (2008). A Pilot Study of Culturally Adapted Cognitive Behavior Therapy for Hispanics with Major Depression. *Cognitive and Behavioral Practice*, *15*(1), 67–75. https://doi.org/10.1016/j.cbpra.2006.12.002

Interian A., Lewis-Fernandez R., & Dixon L.B. (2013). Improving treatment engagement of underserved U.S. racial-ethnic groups: A review of recent interventions. *Psychiatric Services*, *64*(3), 212–222. https://doi.org/10.1176/appi.ps.201100136

Jones, L. V., & Warner, L. A. (2011). Evaluating Culturally Responsive Group Work with Black Women. *Research on Social Work Practice*, *21*(6), 737–746.

Kalibatseva, Z., & Leong, F. T. (2014). A critical review of culturally sensitive treatments for depression: Recommendations for intervention and research. *Psychological Services*, *11*(4), 433–450.

Kaltman, S., de Mendoza, A. H., Serrano, A., & Gonzales, F. A. (2016). A Mental Health Intervention Strategy for Low-Income, Trauma-Exposed Latina Immigrants in Primary Care: A Preliminary Study. *American Journal of Orthopsychiatry*, *86*(3), 345–354.

Kananian, S., Soltani, Y., Hinton, D., & Stangier, U. (2020). Culturally Adapted Cognitive Behavioral Therapy Plus Problem Management (CA‐CBT+) With Afghan Refugees: A Randomized Controlled Pilot Study. *Journal of Traumatic Stress*, *33*(6), 928–938.

Kanter, J. W., Santiago-Rivera, A. L., Rusch, L. C., Busch, A. M., & West, P. (2010). Initial outcomes of a culturally adapted behavioral activation for Latinas diagnosed with depression at a community clinic. *Behavior Modification*, *34*(2), 120–144.

Kanter, J. W., Santiago-Rivera, A. L., Santos, M. M., Nagy, G., Lopez, M., Hurtado, G. D., & West, P. (2015). A randomized hybrid efficacy and effectiveness trial of behavioral activation for Latinos with depression. *Behavior Therapy*, *46*(2), 177–192.

Karasz, A., Raghavan, S., Patel, V., Zaman, M., Akhter, L., & Kabita, M. (2015). ASHA: Using Participatory Methods to Develop an Asset-building Mental Health Intervention for Bangladeshi Immigrant Women. *Progress in Community Health Partnerships : Research, Education, and Action*, *9*(4), 501–512.

Kayrouz, R., Dear, B. F., Johnston, L., Gandy, M., Fogliati, V. J., Sheehan, J., & Titov, N. (2015). A feasibility open trial of guided Internet-delivered cognitive behavioural therapy for anxiety and depression amongst Arab Australians. *Internet Interventions*, *2*(1), 32–38.

Knaevelsrud, C., Brand, J., Lange, A., Ruwaard, J., & Wagner, B. (2015). Web-based psychotherapy for posttraumatic stress disorder in war-traumatized Arab patients: Randomized controlled trial. *Journal of Medical Internet Research*, *17*(3), e71.

Koch, T., Ehring, T., & Liedl, A. (2020). Effectiveness of a transdiagnostic group intervention to enhance emotion regulation in young Afghan refugees: A pilot randomized controlled study. *Behaviour Research and Therapy*, *132*, 103689. https://doi.org/10.1016/j.brat.2020.103689

Kohn, L. P., Oden, T., Muñoz, R. F., Robinson, A., & Leavitt, D. (2002). Adapted cognitive behavioral group therapy for depressed low-income African American women. *Community Mental Health Journal*, *38*(6), 497–504. https://doi.org/10.1023/a:1020884202677

Kruse, J., Joksimovic, L., Cavka, M., Woller, W., & Schmitz, N. (2009). Effects of trauma-focused psychotherapy upon war refugees. *Journal of Traumatic Stress*, *22*(6), 585–592.

Laperriere, A., Antoni, M. H., Grossman, A., Ironson, G. H., Ishii, M., Jones, D., … Pomm, H. (2005). Decreased depression up to one year following CBSM+ intervention in depressed women with AIDS: the smart/EST women’s project. *Journal of Health Psychology*, *10*(2), 223–231.

Leiler, A., Wasteson, E., Holmberg, J., & Bjärtå, A. (2020). A Pilot Study of a Psychoeducational Group Intervention Delivered at Asylum Accommodation Centers-A Mixed Methods Approach. *International Journal of Environmental Research and Public Health*, *17*(23). https://doi.org/10.3390/ijerph17238953

Leske, S., Harris, M. G., Charlson, F. J., Ferrari, A. J., Baxter, A. J., Logan, J. M., … Whiteford, H. (2016). Systematic review of interventions for Indigenous adults with mental and substance use disorders in Australia, Canada, New Zealand and the United States. *Australian & New Zealand Journal of Psychiatry*, *50*(11), 1040–1054.

Lindegaard, T., Brohede, D., Koshnaw, K., Osman, S. S., Johansson, R., & Andersson, G. (2019). Internet-based treatment of depressive symptoms in a Kurdish population: A randomized controlled trial. *Journal of Clinical Psychology*, *75*(6), 985–998.

Lindegaard, T., Seaton, F., Halaj, A., Berg, M., Kashoush, F., Barchini, R., … Andersson, G. (2020). Internet-based cognitive behavioural therapy for depression and anxiety among Arabic-speaking individuals in Sweden: A pilot randomized controlled trial. *Cognitive Behaviour Therapy*, *50*(1), 47–66. https://doi.org/10.1080/16506073.2020.1771414

Lovell, K., Lamb, J., Gask, L., Bower, P., Waheed, W., Chew-Graham, C., … Dowrick, C. (2014). Development and evaluation of culturally sensitive psychosocial interventions for under-served people in primary care. *BMC Psychiatry*, *14*, 217.

Matsumoto, K., Hamatani, S., Nagai, K., Sutoh, C., Nakagawa, A., & Shimizu, E. (2020). Long-Term Effectiveness and Cost-Effectiveness of Videoconference-Delivered Cognitive Behavioral Therapy for Obsessive-Compulsive Disorder, Panic Disorder, and Social Anxiety Disorder in Japan: One-Year Follow-Up of a Single-Arm Trial. *JMIR Mental Health*, *7*(4). Retrieved from https://mental.jmir.org/2020/4/e17157/

Meffert, S. M., Abdo, A. O., Alla, O. A. A., Elmakki, Y. O. M., Omer, A. A., Yousif, S., … Marmar, C. R. (2014). A pilot randomized controlled trial of interpersonal psychotherapy for Sudanese refugees in Cairo, Egypt. *Psychological Trauma: Theory, Research, Practice, and Policy*, *6*(3), 240–249.

Miranda, J., Azocar, F., Organista, K. C., Dwyer, E., & Areane, P. (2003). Treatment of depression among impoverished primary care patients from ethnic minority groups. *Psychiatric Services (Washington, D.C.)*, *54*(2), 219–225.

Muto, T., Hayes, S. C., & Jeffcoat, T. (2011). The effectiveness of acceptance and commitment therapy bibliotherapy for enhancing the psychological health of Japanese college students living abroad. *Behavior Therapy*, *42*(2), 323–335.

Naeem, Faooq, Sarhandi, I., Gul, M., Khalid, M., Aslam, M., Anbrin, A., … Ayub, M. (2014). A multicentre randomised controlled trial of a carer supervised culturally adapted CBT (CaCBT) based self-help for depression in Pakistan. *Journal of Affective Disorders*, *156*, 224–227. https://doi.org/10.1016/j.jad.2013.10.051

Naeem, Farooq, Phiri, P., Munshi, T., Rathod, S., Ayub, M., Gobbi, M., & Kingdon, D. (2015). Using cognitive behaviour therapy with South Asian Muslims: Findings from the culturally sensitive CBT project. *International Review of Psychiatry*, *27*(3), 233–246. https://doi.org/10.3109/09540261.2015.1067598

Neuner, F., Onyut, P. L., Ertl, V., Odenwald, M., Schauer, E., & Elbert, T. (2008). Treatment of posttraumatic stress disorder by trained lay counselors in an African refugee settlement: A randomized controlled trial. *Journal of Consulting and Clinical Psychology*, *76*(4), 686–694.

Palic, S., & Elklit, A. (2011). Psychosocial treatment of posttraumatic stress disorder in adult refugees: A systematic review of prospective treatment outcome studies and a critique. *Journal of Affective Disorders*, *131*(1), 8–23.

Pan, D., Huey, Jr., S. J., & Hernandez, D. (2011). Culturally adapted versus standard exposure treatment for phobic Asian Americans: Treatment efficacy, moderators, and predictors. *Cultural Diversity & Ethnic Minority Psychology*, *17*(1), 11–22.

Piedra, L. M., & Byoun, S.-J. (2011). Vida Alegre: Preliminary Findings of a Depression Intervention for Immigrant Latino Mothers. *Research on Social Work Practice*, *22*(2), 138–150. https://doi.org/10.1177/1049731511424168

Pineros-Leano, M., Liechty, J. M., & Piedra, L. M. (2017). Latino immigrants, depressive symptoms, and cognitive behavioral therapy: A systematic review. *Journal of Affective Disorders*, *208*, 567–576.

Rathod, S., Phiri, P., Harris, S., Underwood, C., Thagadur, M., Padmanabi, U., & Kingdon, D. (2013). Cognitive behaviour therapy for psychosis can be adapted for minority ethnic groups: A randomised controlled trial. *Schizophrenia Research*, *143*(2), 319–326.

Razali, S. M., Aminah, K., & Khan, U. A. (2002). Religious-cultural psychotherapy in the management of anxiety patients. *Transcultural Psychiatry*, *39*(1), 130–136.

Razali, S. M., Hasanah, C. I., Aminah, K., & Subramaniam, M. (1998). Religious–sociocultural psychotherapy in patients with anxiety and depression. *The Australian and New Zealand Journal of Psychiatry*, *32*(6), 867–872.

Rosmarin, D. H., Pargament, K. I., Pirutinsky, S., & Mahoney, A. (2010). A randomized controlled evaluation of a spiritually integrated treatment for subclinical anxiety in the Jewish community, delivered via the Internet. *Journal of Anxiety Disorders*, *24*(7), 799–808.

Ryan, D., Maurer, S., Lengua, L., Duran, B., & Ornelas, I. J. (2018). Amigas Latinas Motivando el Alma (ALMA): An Evaluation of a Mindfulness Intervention to Promote Mental Health among Latina Immigrant Mothers. *The Journal of Behavioral Health Services & Research*, *45*(2), 280–291.

Sander, R., Laugesen, H., Skammeritz, S., Mortensen, E. L., & Carlsson, J. (2019). Interpreter-mediated psychotherapy with trauma-affected refugees—A retrospective cohort study. *Psychiatry Research*, *271*, 684–692.

Scogin, F., Morthland, M., Kaufman, A., Burgio, L., Chaplin, W., & Kong, G. (2007). Improving Quality of Life in Diverse Rural Older Adults: A Randomized Trial of a Psychological Treatment. *Psychology and Aging*, *22*(4), 657–665.

Shaw, S. A., Ward, K. P., Pillai, V., & Hinton, D. E. (2018). A Group Mental Health Randomized Controlled Trial for Female Refugees in Malaysia. *American Journal of Orthopsychiatry*.

Shin, S. K., & Lukens, E. P. (2002). Effects of psychoeducation for Korean Americans with chronic mental illness. *Psychiatric Services*, *53*(9), 1125–1131.

So, S. H.-W., Chan, A. P., Chong, C. S.-Y., Wong, M. H.-M., Lo, W. T.-L., Chung, D. W.-S., & Chan, S. S. (2015). Metacognitive training for delusions (MCTd): Effectiveness on data-gathering and belief flexibility in a Chinese sample. *Frontiers in Psychology*, *6*. Retrieved from http://ovidsp.ovid.com/ovidweb.cgi?T=JS&PAGE=reference&D=psyc12&NEWS=N&AN=2015-55172-001

Sonderegger, R., Rombouts, S., Ocen, B., & McKeever, R. S. (2011). Trauma rehabilitation for war-affected persons in northern Uganda: A pilot evaluation of the EMPOWER programme. *The British Journal of Clinical Psychology*, *50*(3), 234–249.

Tol, W. A., Leku, M. R., Lakin, D. P., Carswell, K., Augustinavicius, J., Adaku, A., … van Ommeren, M. (2020). Guided self-help to reduce psychological distress in South Sudanese female refugees in Uganda: A cluster randomised trial. *The Lancet Global Health*, *8*(2), e254–e263.

Van Loon A., Van Schaik A., Dekker J., & Beekman A. (2013). Bridging the gap for ethnic minority adult outpatients with depression and anxiety disorders by culturally adapted treatments. *Journal of Affective Disorders*, *147*(1–3), 9–16. https://doi.org/10.1016/j.jad.2012.12.014

Ward, E. C., & Brown, R. L. (2015). A culturally adapted depression intervention for African American adults experiencing depression: Oh Happy Day. *The American Journal of Orthopsychiatry*, *85*(1), 11–22.
